# Supplementary material for: Polyporenic Acids from the Mushroom Buglossoporus quercinus Possess Chemosensitizing and Efflux Pump Inhibitory Activities on Colo 320 Adenocarcinoma Cells
Source: J Fungi (Basel). 2023 Sep 13;9(9):923. doi: 10.3390/jof9090923 (PMC10532983; doi:10.3390/jof9090923)
Supplement: Supplementary file 1 [file jof-09-00923-s001.zip › jof-2601181-supplementary.pdf]

Polyporenic Acids from The Mushroom *Buglossoporus quercinus* Possess Chemosensitizing and Efflux Pump Inhibitory Activities on Colo 320 Adenocarcinoma Cells

Kristóf Felegyi[1], Zsófia Garádi[1, 2], Bálint Rácz[3], Gábor Tóth[3,4], Viktor Papp[5], Imre Boldizsár [1,6], András Dancsó[2], Gabriella Spengler [3], Szabolcs Béni[1,7] and Attila Ványolós[1,]*

[1] Department of Pharmacognosy, Semmelweis University, 1085 Budapest, Hungary; felegyi.kristof@pharma.semmelweis-univ.hu (K.F.); garadi.zsofia@pharma.semmelweis-univ.hu (Z.G.); boldizsar.imre@semmelweis.hu (I.B.); beni.szabolcs@semmelweis.hu (S.B.)

[2] Directorate of Drug Substance Development, Egis Pharmaceuticals Plc., 1475 Budapest, Hungary; dancso.andras@egis.hu

[3] Albert Szent-Györgyi Health Center, Department of Medical Microbiology, Albert Szent-Györgyi Medical School, University of Szeged, 6725 Szeged, Hungary; racz.balint@med.u-szeged.hu (B.R.); toth.gabor.1@med.u-szeged.hu (G.T.); spengler.gabriella@med.u-szeged.hu (G.S.)

[4] ELKH-USZ Biologically Active Natural Products Research Group, University of Szeged, 6720 Szeged, Hungary

[5] Department of Botany, Hungarian University of Agriculture and Life Sciences, 1118 Budapest, Hungary; papp.viktor@uni-mate.hu

[6] Department of Plant Anatomy, Institute of Biology, Eötvös Loránd University, 1117 Budapest, Hungary

[7] Department of Analytical Chemistry, Institute of Chemistry, Eötvös Loránd University, 1117 Budapest, Hungary

* Correspondence: vanyolos.attila@semmelweis.hu

TABLE OF CONTENTS

Figure S1. HR-ESI-MS spectrum of compound **1** 6

Figure S2. MS-MS spectrum of compound **1** 7

Figure S3. ^{1}H spectrum of compound **1** (600 MHz, THF-d_8, 295 K) 7

Figure S4. ^{13}C spectrum of compound **1** (150 MHz, THF- d_8, 295 K) 8

Figure S5. COSY spectrum of compound **1** (600 MHz, THF- d_8, 295 K) 8

Figure S6. DEPT-edited HSQC spectrum of compound **1** (600 MHz, THF- d_8, 295 K) 9

Figure S7. HMBC spectrum of compound **1** (600 MHz, THF- d_8, 295 K) 10

Figure S8. NOESY spectrum of compound **1** (600 MHz, THF- d_8, 295 K) 11

Figure S9. Selected region of the NOESY spectrum of compound **1** (600 MHz, THF- d_8, 295 K) 11

Figure S10. HR-ESI-MS spectrum of compound **2** 12

Figure S11. MS-MS spectrum of compound **2** 13

Figure S12. ^{1}H spectrum of compound **2** (600 MHz, THF- d_8, 295 K) 13

Figure S13. ^{13}C spectrum of compound **2** (150 MHz, THF- d_8, 295 K) 14

Figure S14. COSY spectrum of compound **2** (600 MHz, THF- d_8, 295 K) 14

Figure S15. DEPT-edited HSQC spectrum of compound **2** (600 MHz, THF- d_8, 295 K) 15

Figure S16. HMBC spectrum of compound **2** (600 MHz, THF- d_8, 295 K) 16

Figure S17. NOESY spectrum of compound **2** (600 MHz, THF- d_8, 295 K) 17

Figure S18. Selected region of the NOESY spectrum of compound **2** (600 MHz, THF- d_8, 295 K) 17

Figure S19. HR-ESI-MS spectrum of compound **3** 18

Figure S20. MS-MS spectrum of compound **3** 19

Figure S21. ^{1}H spectrum of compound **3** (600 MHz, $CDCl_3$, 295 K) 19

Figure S22. ^{13}C spectrum of compound **3** (150 MHz, $CDCl_3$, 295 K) 20

Figure S23. COSY spectrum of compound **3** (600 MHz, $CDCl_3$, 295 K) 20

Figure S24. DEPT-edited HSQC spectrum of compound **3** (600 MHz, $CDCl_3$, 295 K) 21

Figure S25. HMBC spectrum of compound **3** (600 MHz, $CDCl_3$, 295 K) 21

Figure S26. NOESY spectrum of compound **3** (600 MHz, $CDCl_3$, 295 K) 22

Figure S27. HR-ESI-MS spectrum of compund **4** 23

Figure S28. MS-MS spectrum of compound **4** 23

Figure S29. ^{1}H spectrum of compound **4** (600 MHz, $CDCl_3$, 295 K) 24

Figure S30. ^{13}C spectrum of compound **4** (150 MHz, $CDCl_3$, 295 K) 24

Figure S31. COSY spectrum of compound **4** (600 MHz, $CDCl_3$, 295 K) 25

Figure S32. DEPT-edited HSQC spectrum of compound **4** (600 MHz, $CDCl_3$, 295 K) 25

Figure S33. HMBC spectrum of compound **4** (600 MHz, $CDCl_3$, 295 K) 26

Figure S34. Selective gradient NOESY spectrum of compound **4** (600 MHz, $CDCl_3$, 295 K) 26

Figure S35. HR-ESI-MS spectrum of compound **5** 27

Figure S36. MS-MS spectrum of compound **5** 28

Figure S37. ^{1}H spectrum of compound **5** (600 MHz, pyridine-d_5, 295 K) 28

Figure S38. ^{13}C spectrum of compound **5** (150 MHz, pyridine- d_5, 295 K) 29

Figure S39. COSY spectrum of compound **5** (600 MHz, pyridine- d_5, 295 K) 29

Figure S40. DEPT-edited HSQC spectrum of compound **5** (600 MHz, pyridine- d_5, 295 K) 30

Figure S41. HMBC spectrum of compound **5** (600 MHz, pyridine- d_5, 295 K) 30

Figure S42. DEPT-edited HSQC spectrum of compound **5** (600 MHz, CD_3OD : pyridine- d_5, 295 K) 31

Figure S43. HMBC spectrum of compound **5** (600 MHz, CD_3OD : pyridine- d_5 (19:1), 295 K) 31

Figure S44. ROESY spectrum of compound **5** (600 MHz, CD_3OD : pyridine- d_5 (19:1), 295 K) 32

Figure S45. HR-ESI-MS spectrum of compound **6** 33

Figure S46. MS-MS spectrum of compound **6** 33

Figure S47. ^{1}H spectrum of compound **6** (150 MHz, CD_3OD, 295 K) 34

Figure S48. ^{13}C spectrum of compound **6** (600 MHz, CD_3OD, 295 K) 34

Figure S49. COSY spectrum of compound **6** (600 MHz, CD_3OD, 295 K) 35

Figure S50. DEPT-edited HSQC spectrum of compound **6** (600 MHz CD_3OD, 295 K) 35

Figure S51. HMBC spectrum of compound **6** (600 MHz, CD_3OD, 295 K) 36

Figure S52. NOESY spectrum of compound **6** (600 MHz, CD_3OD, 295 K) 36

Figure S53. ^{1}H spectrum of compound **6** (600 MHz, $CDCl_3$, 295 K) 37

Figure S54. DEPT-edited HSQC spectrum of compound **6** (600 MHz, $CDCl_3$, 295 K) 37

Figure S55. HMBC spectrum of compound **6** (600 MHz, $CDCl_3$, 295 K) 38

Figure S56. HR-ESI-MS spectrum of compound **7** 39

Figure S57. MS-MS spectrum of compound **7** 40

Figure S58. ^{1}H spectrum of compound **7** (600 MHz, $CDCl_3$, 295 K) 40

Figure S59. ^{13}C spectrum of compound **7** (150 MHz, $CDCl_3$, 295 K), 41

Figure S60. COSY spectrum of compound **7** (600 MHz, $CDCl_3$, 295 K) 41

Figure S61. DEPT-edited HSQC spectrum of compound **7** (600 MHz, $CDCl_3$, 295 K) 42

Figure S62. HMBC spectrum of compound **7** (600 MHz, $CDCl_3$, 295 K) 42

Figure S63. ROESY spectrum of compound **7** (600 MHz, $CDCl_3$, 295 K) 43

Figure S64. HR-ESI-MS spectrum of compound **8** 44

Figure S65. MS-MS spectrum of compound **8** 45

Figure S66. ^{1}H spectrum of compound **8** (600 MHz, $CDCl_3$, 295 K) 45

Figure S67. ^{13}C spectrum of compound **8** (150 MHz, $CDCl_3$, 295 K) 46

Figure S68. COSY spectrum of compound **8** (600 MHz, $CDCl_3$, 295 K) 46

Figure S69. DEPT-edited HSQC spectrum of compound **8** (600 MHz, $CDCl_3$, 295 K) 47

Figure S70. HMBC spectrum of compound **8** (600 MHz, $CDCl_3$, 295 K) 47

Figure S71. ROESY spectrum of compound **8** (600 MHz, $CDCl_3$, 295 K) 48
Figure S72. HR-ESI-MS spectrum of compound **9** 49
Figure S73. MS-MS spectrum of compound **9** 50
Figure S74. ^{1}H spectrum of compound **9** (600 MHz, $CDCl_3$, 295 K) 50
Figure S75. ^{13}C spectrum of compound **9** (150 MHz, $CDCl_3$, 295 K) 51
Figure S76. COSY spectrum of compound **9** (600 MHz, $CDCl_3$, 295 K) 51
Figure S77. DEPT-edited HSQC spectrum of compound **9** (600 MHz, $CDCl_3$, 295 K) 52
Figure S78. HMBC spectrum of compound **9** (600 MHz, $CDCl_3$, 295 K) 52
Figure S79. NOESY spectrum of compound **9** (600 MHz, $CDCl_3$, 295 K) 53
Figure S80. NOESY spectrum of compound **9** (600 MHz, $CDCl_3$, 295 K) 53
Figure S81. HR-ESI-MS spectrum of compound **10** 54
Figure S82. MS-MS spectrum of compound **10** 55
Figure S83. ^{1}H spectrum of compound **10** (600 MHz, pyridine-d_5, 295 K) 55
Figure S84. ^{13}C spectrum of compound **10** (150 MHz, pyridine-d_5, 295 K) 56
Figure S85. COSY spectrum of compound **10** (600 MHz, pyridine-d_5, 295 K) 56
Figure S86. DEPT-edited HSQC spectrum of compound **10** (600 MHz, pyridine-d_5, 295 K) 57
Figure S87. HMBC spectrum of compound **10** (600 MHz, pyridine-d_5, 295 K) 57
Figure S88. NOESY spectrum of compound **10** (600 MHz, pyridine-d_5, 295 K) 58
Figure S89. HR-ESI-MS spectrum of compound **11** 59
Figure S90. MS-MS spectrum of compound **11** 60
Figure S91. ^{1}H spectrum of compound **11** (600 MHz, $CDCl_3$, 295 K) 60
Figure S92. ^{13}C spectrum of compound **11** (150 MHz, $CDCl_3$, 295 K) 61
Figure S93. COSY spectrum of compound **11** (600 MHz, $CDCl_3$, 295 K) 61
Figure S94. DEPT-edited HSQC spectrum of compound **11** (600 MHz, $CDCl_3$, 295 K) 62
Figure S95. HMBC spectrum of compound **11** (600 MHz, $CDCl_3$, 295 K) 62
Figure S96. NOESY spectrum of compound **11** (600 MHz, $CDCl_3$, 295 K) 63
Figure S97. HR-ESI-MS spectrum of compound **12** 64
Figure S98. MS-MS spectrum of compound **12** 65
Figure S99. ^{1}H spectrum of compound **12** (600 MHz, pyridine-d_5, 295 K) 65
Figure S100. ^{13}C spectrum of compound **12** (150 MHz, pyridine-d_5, 295 K) 66
Figure S101. COSY spectrum of compound **12** (600 MHz, pyridine-d_5, 295 K) 66
Figure S102. DEPT-edited HSQC spectrum of compound **12** (600 MHz, pyridine-d_5, 295 K) 67
Figure S103. HMBC spectrum of compound **12** (600 MHz, pyridine-d_5, 295 K) 67
Figure S104. NOESY spectrum of compound **12** (600 MHz, pyridine-d_5, 295 K) 68
Table S1. NMR Spectroscopic Data (600 MHz, $CDCl_3$[a], pyridine-*d*$_5$[b], tetrahydrofuran-*d*$_8$[c], or methanol-*d*:pyridine-*d*$_5$ 19:1[d], methanol-*d*[e]) for compounds **6-9** 69

Table S2. NMR Spectroscopic Data (600 MHz, $CDCl_3$[a], pyridine-d_5[b], tetrahydrofuran-d_8[c], or methanol-*d*:pyridine-d_5 19:1[d], methanol-*d*[e]) for compounds **10-12** 70

Table S3. Chemosensitizing Activity of Compounds **3-4**, and **7**-**12** on Colo 320 Adenocarcinoma Cells 71

Table S4. P-gp Efflux Pump Inhibitory Activity of Compounds **1**−**12** on MDR Colo 320 Colon Adenocarcinoma Cell Line 73

Spectra and spectral data on compound 1

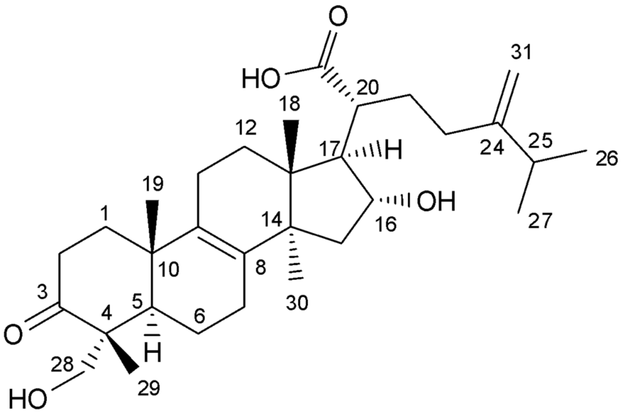

HR-ESI-MS (+) *m/z* 501.3568 [M + H]$^+$ (501.3575 calcd. for $C_{31}H_{49}O_5$; *Δ* -1.3 ppm); HR-ESI-MSMS (CID = 15%, 30%, 45%) 483.3460, 465.3365, 453.3355

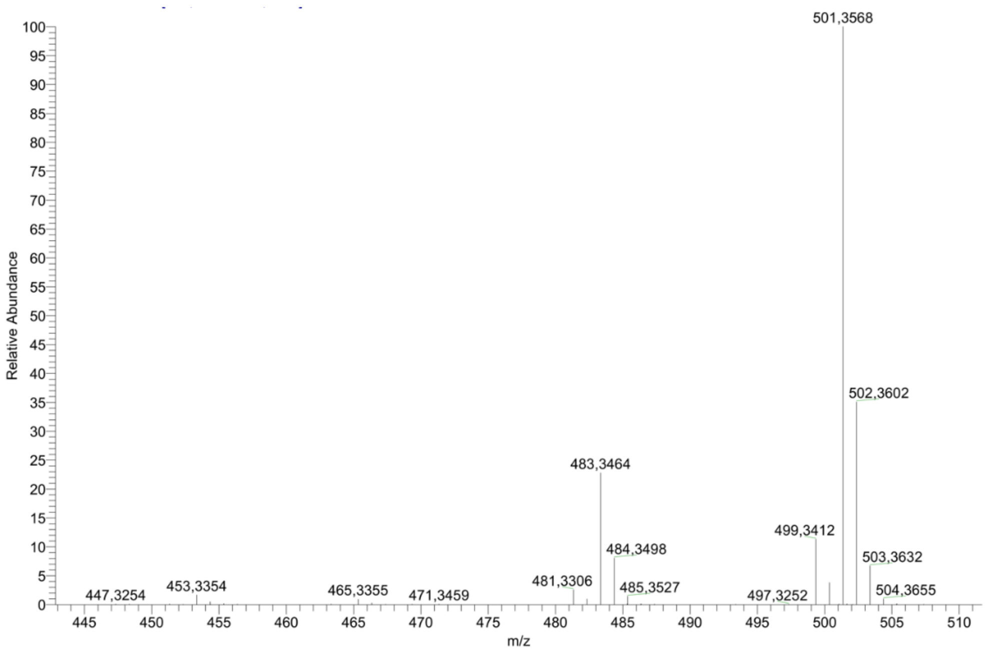

Figure S1. HR-ESI-MS spectrum of compound **1**

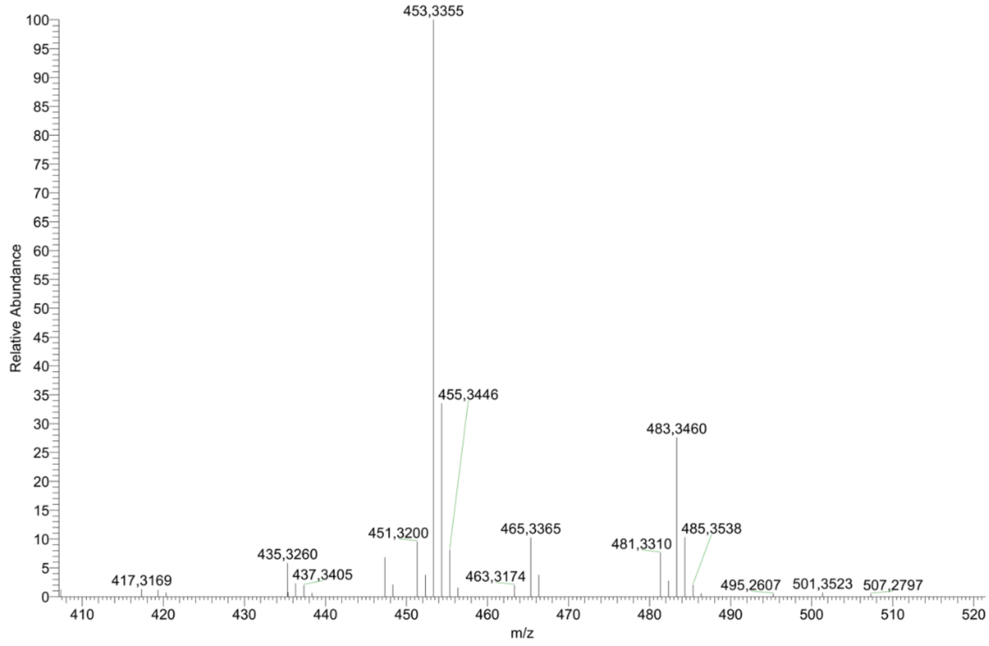

Figure S2. MS-MS spectrum of compound **1**

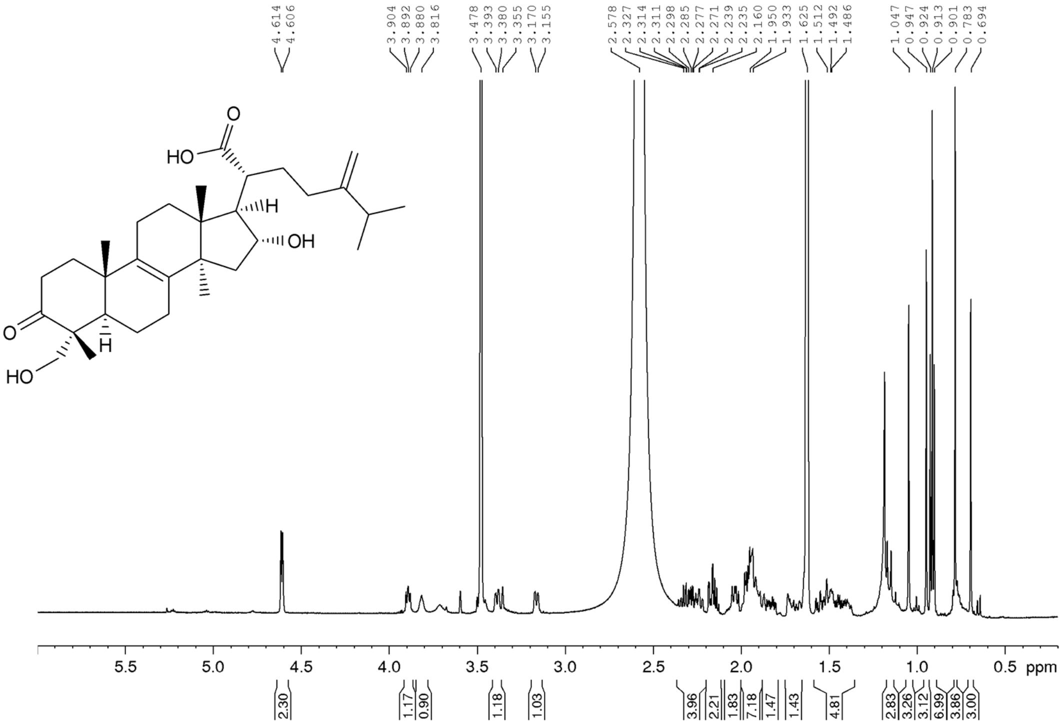

Figure S3. ^{1}H spectrum of compound **1** (600 MHz, THF-d_8, 295 K)

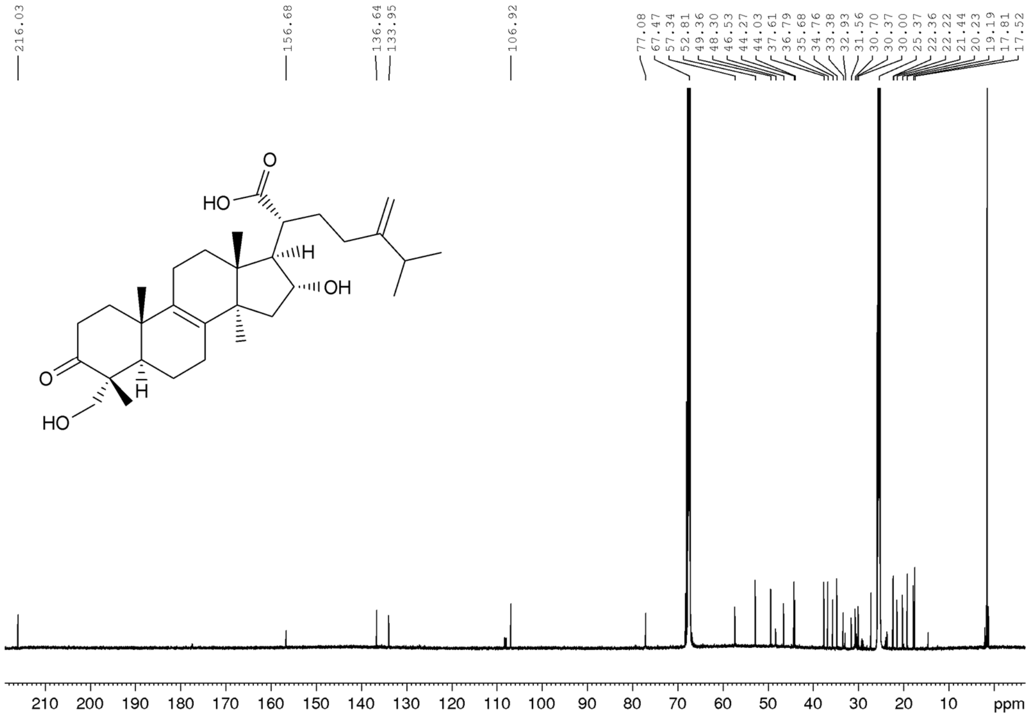

Figure S4. ^{13}C spectrum of compound **1** (150 MHz, THF-d_8, 295 K)

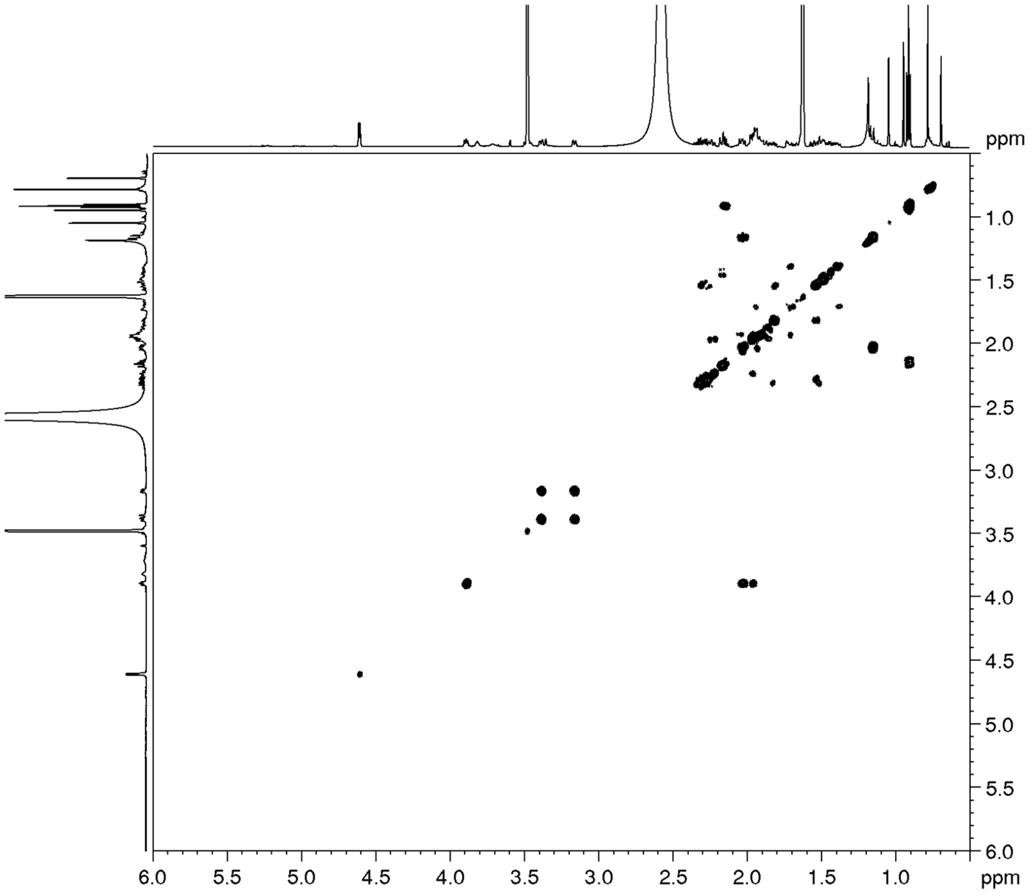

Figure S5. COSY spectrum of compound **1** (600 MHz, THF-d_8, 295 K)

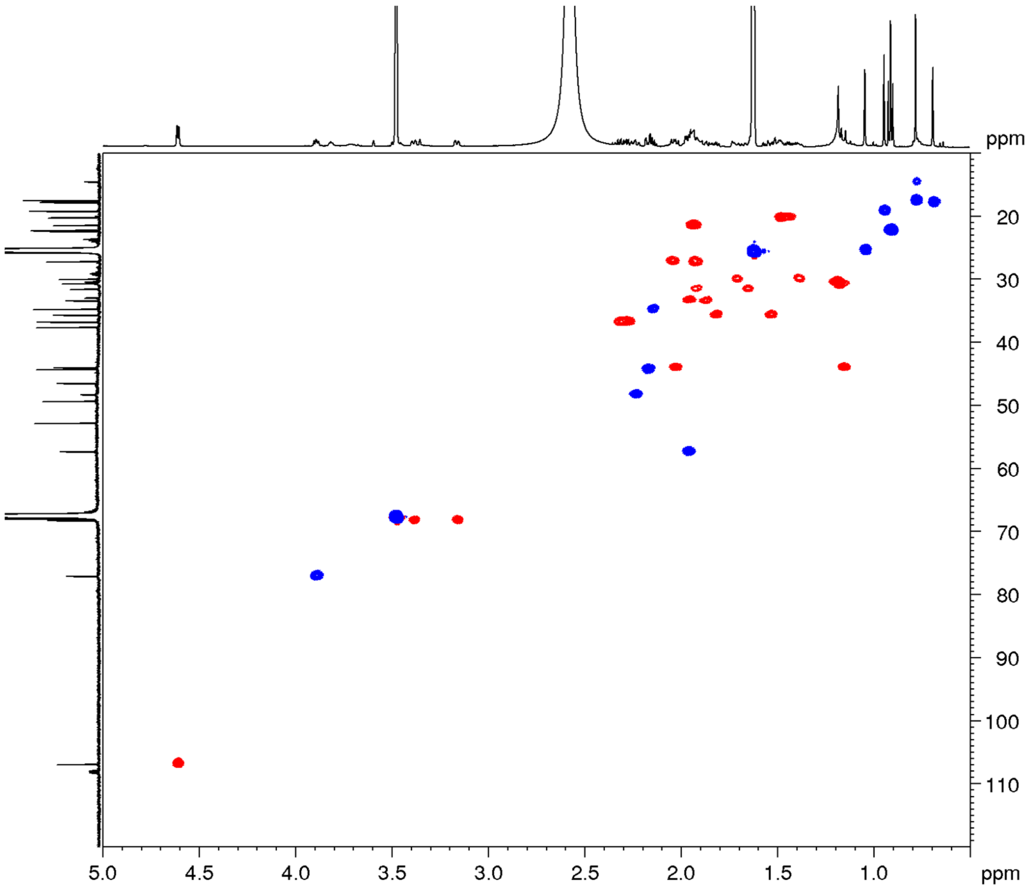

Figure S6. DEPT-edited HSQC spectrum of compound **1** (600 MHz, THF-d_8, 295 K)

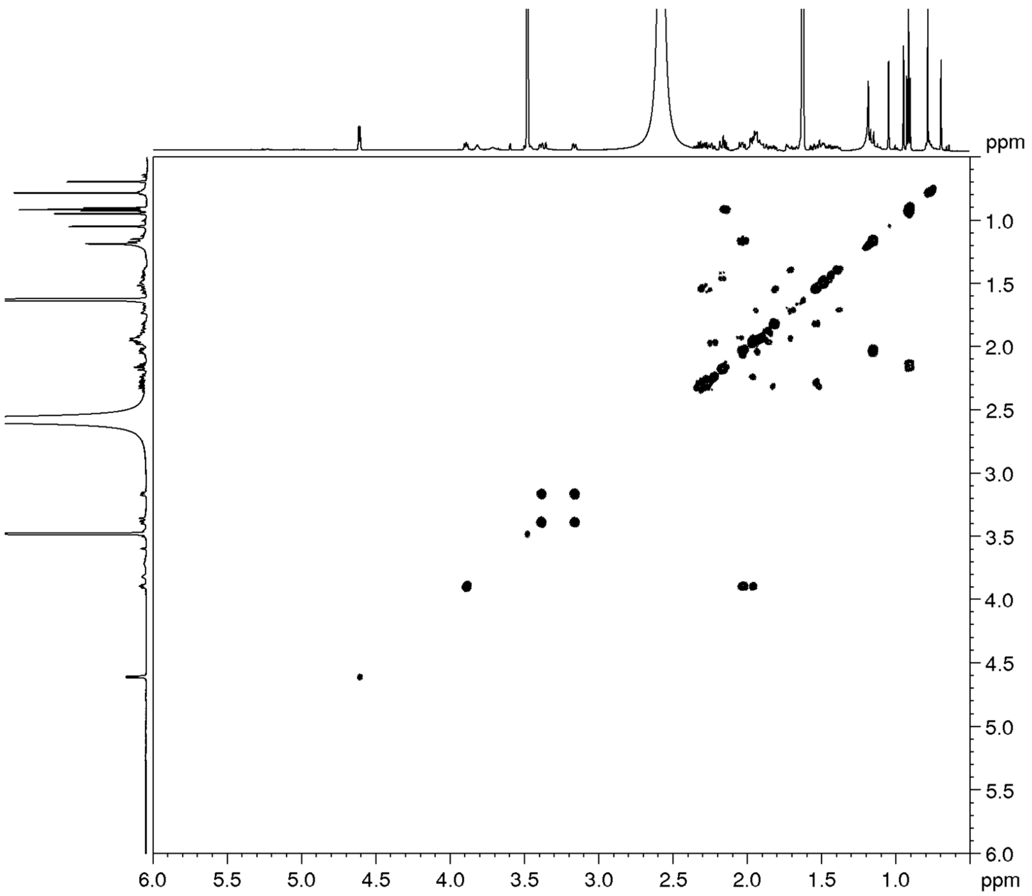

Figure S7. HMBC spectrum of compound **1** (600 MHz, THF-d_8, 295 K)

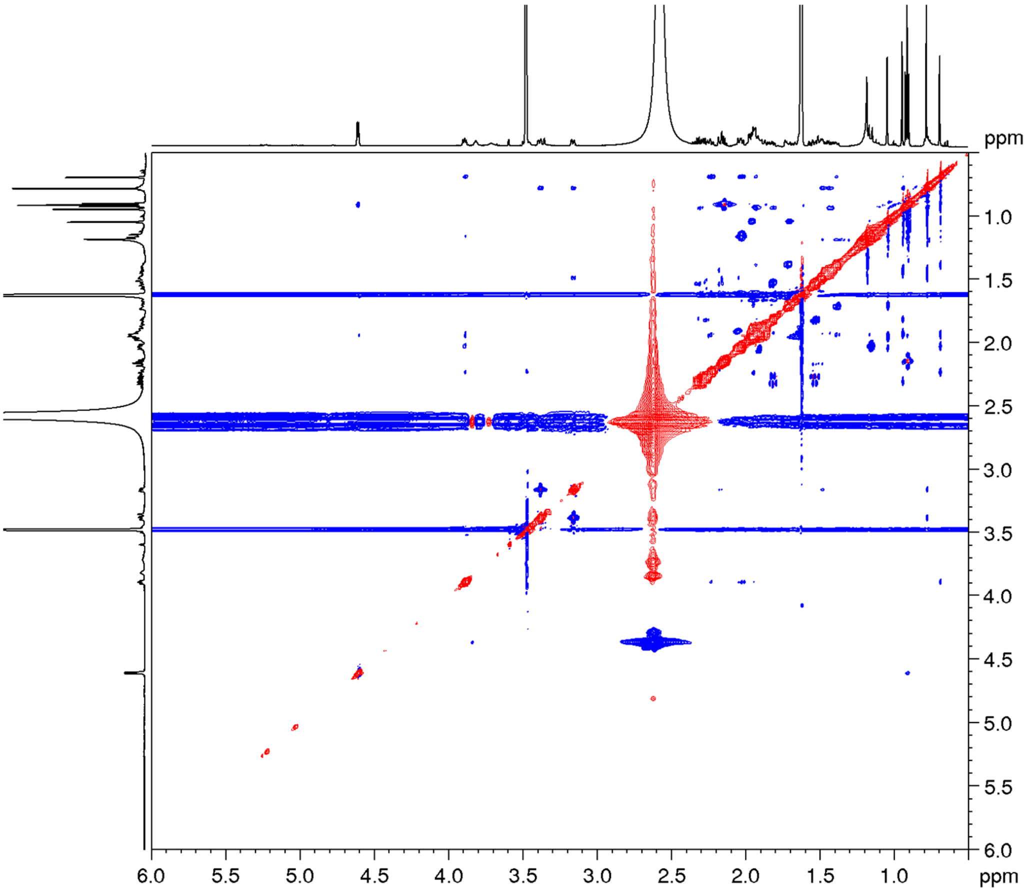

Figure S8. NOESY spectrum of compound **1** (600 MHz, THF-d_8, 295 K)

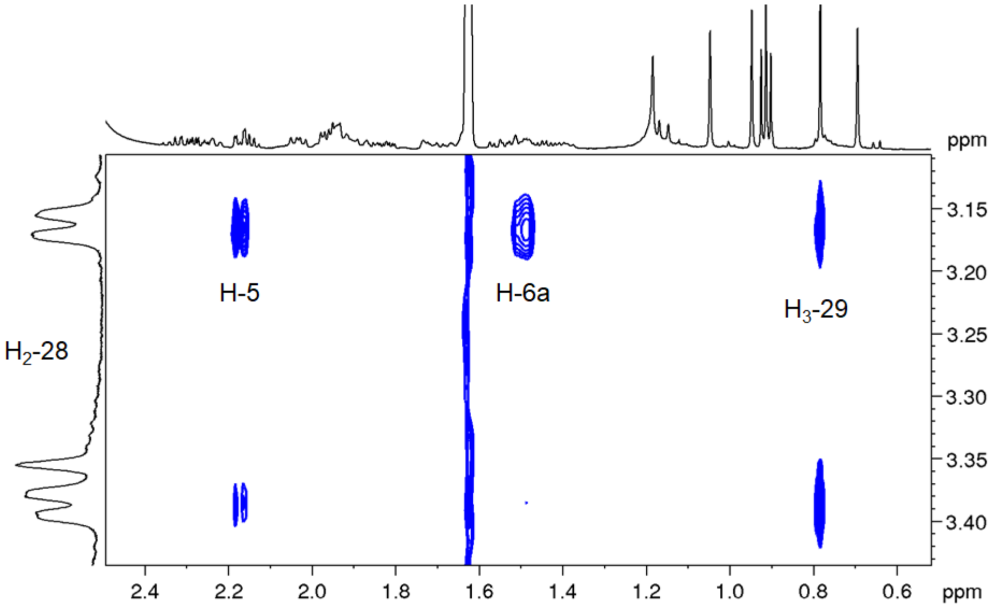

Figure S9. Selected region of the NOESY spectrum of compound **1** (600 MHz, THF-d_8, 295 K)

Spectra and spectral data on compound 2

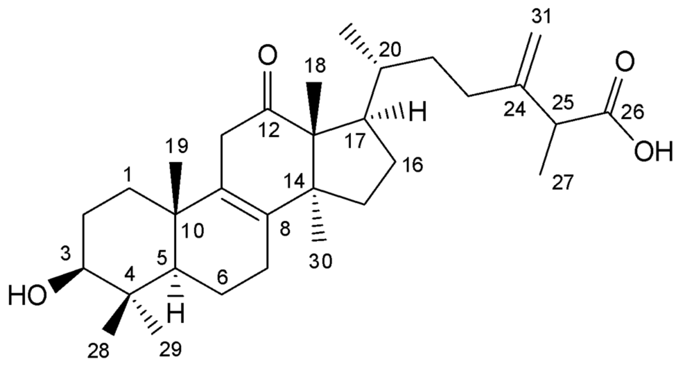

HR-ESI-MS (+) *m/z* 485.3620 $[M + H]^+$ (485.3625 calcd. for $C_{31}H_{49}O_4$; Δ -1.0 ppm); HR-ESI-MSMS (CID = 15%, 30%, 45%) 468.9432, 454.8377

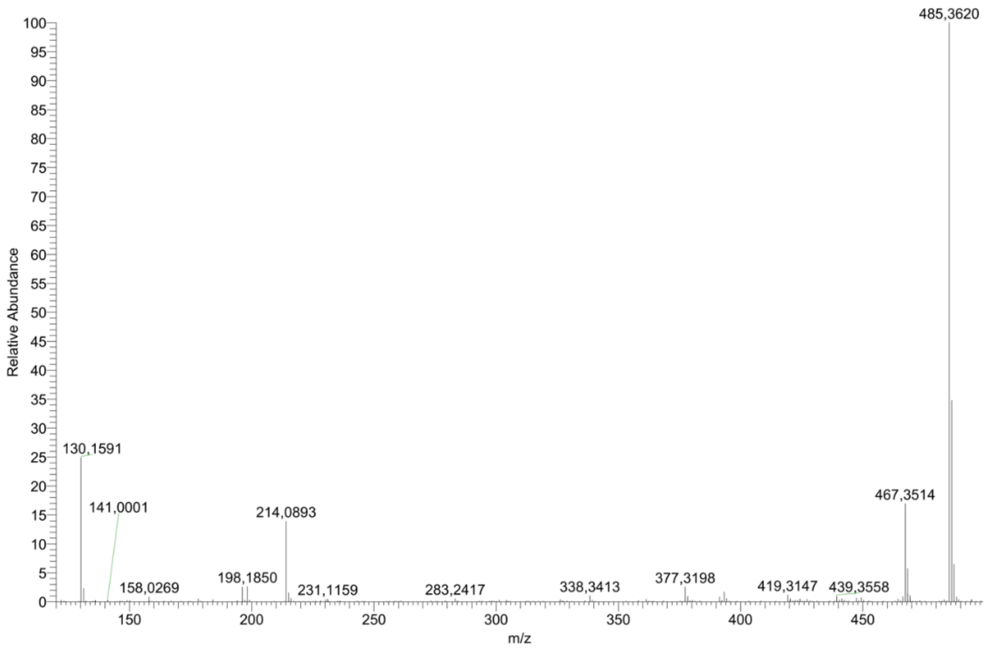

Figure S10. HR-ESI-MS spectrum of compound **2**

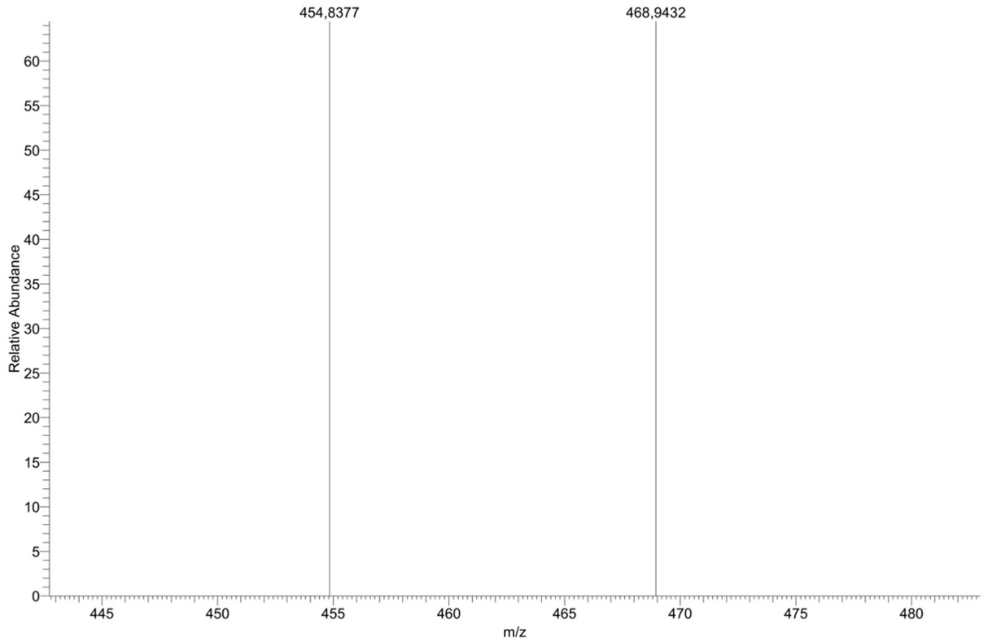

Figure S11. MS-MS spectrum of compound **2**

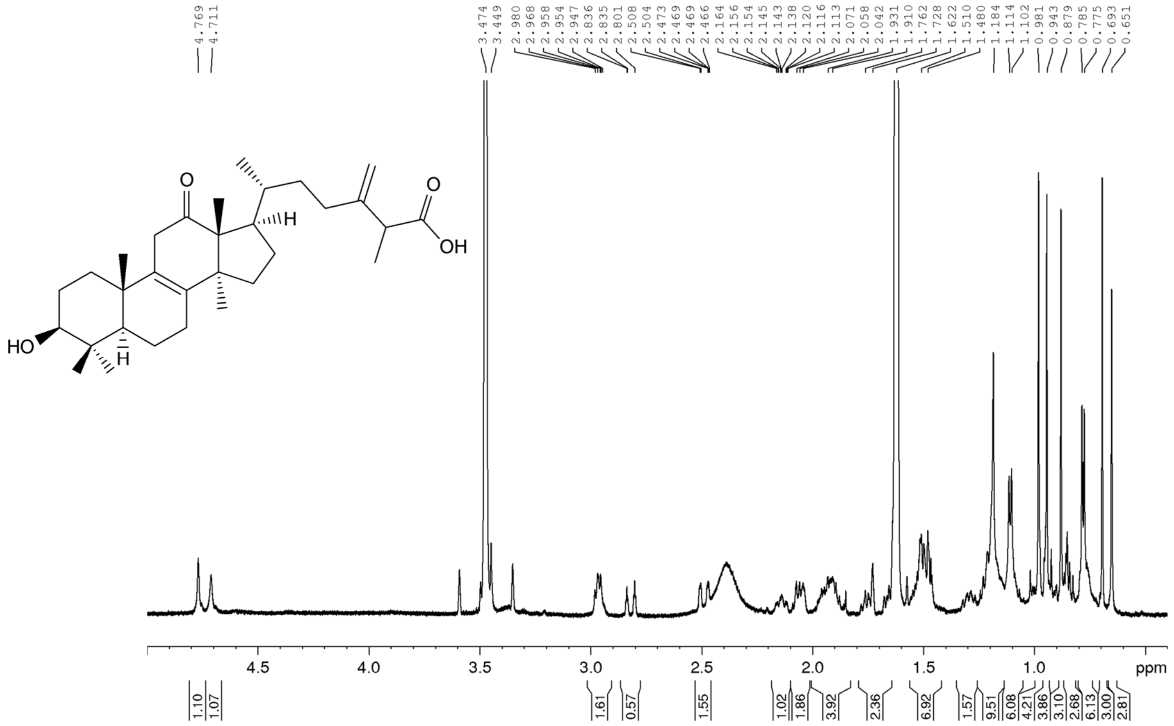

Figure S12. ^{1}H spectrum of compound **2** (600 MHz, THF- d_8, 295 K)

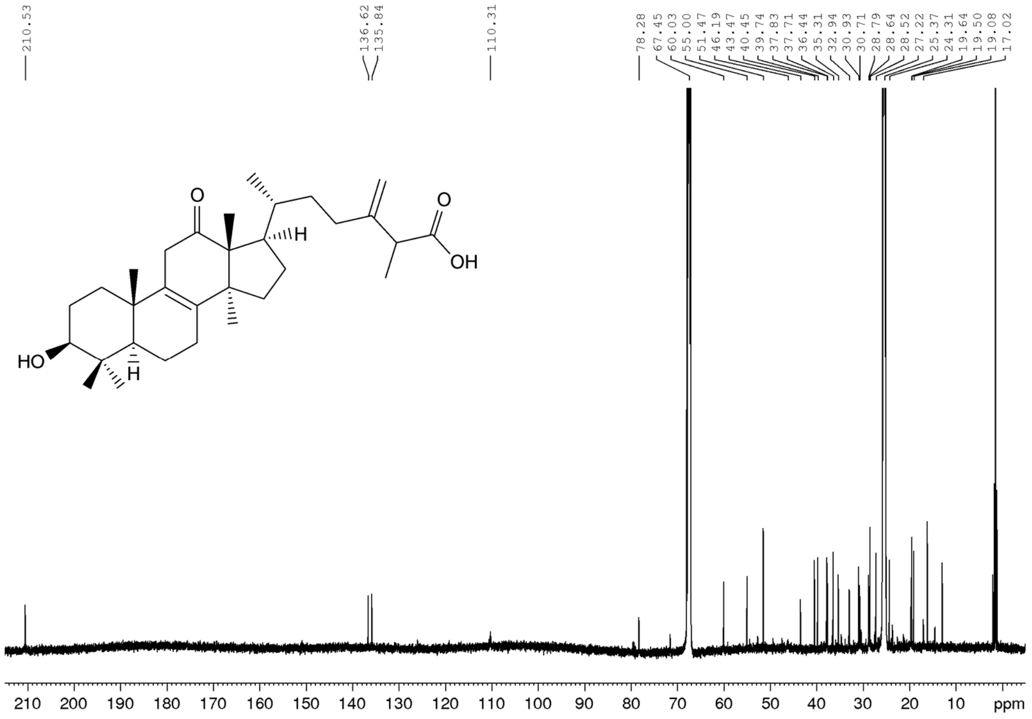

Figure S13. ^{13}C spectrum of compound **2** (150 MHz, THF- d_8, 295 K)

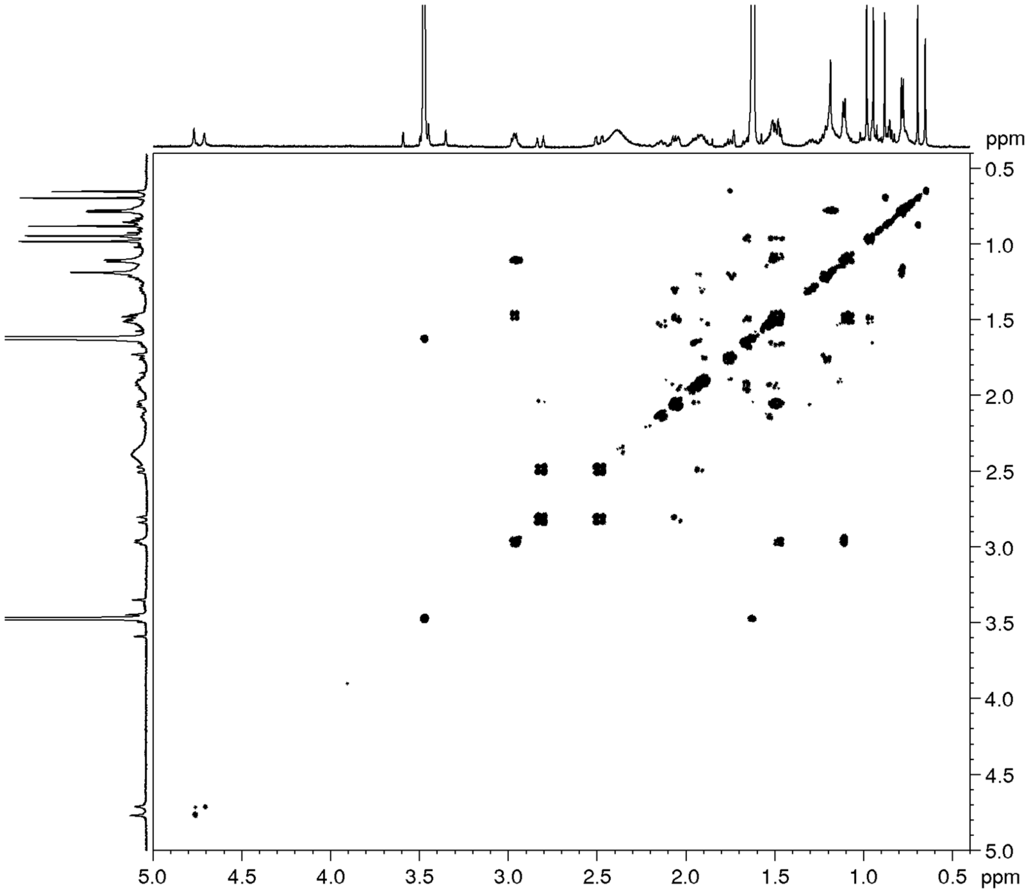

Figure S14. COSY spectrum of compound **2** (600 MHz, THF-d_8, 295 K)

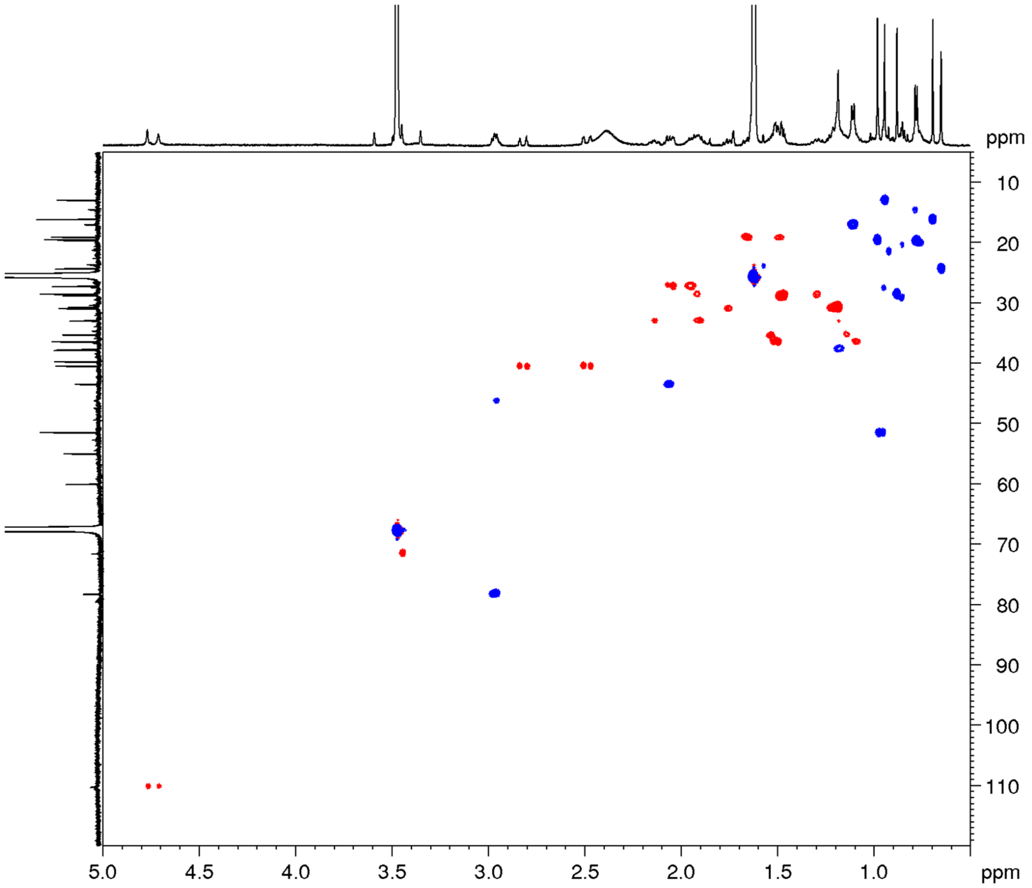

Figure S15. DEPT-edited HSQC spectrum of compound **2** (600 MHz, THF-d_8, 295 K)

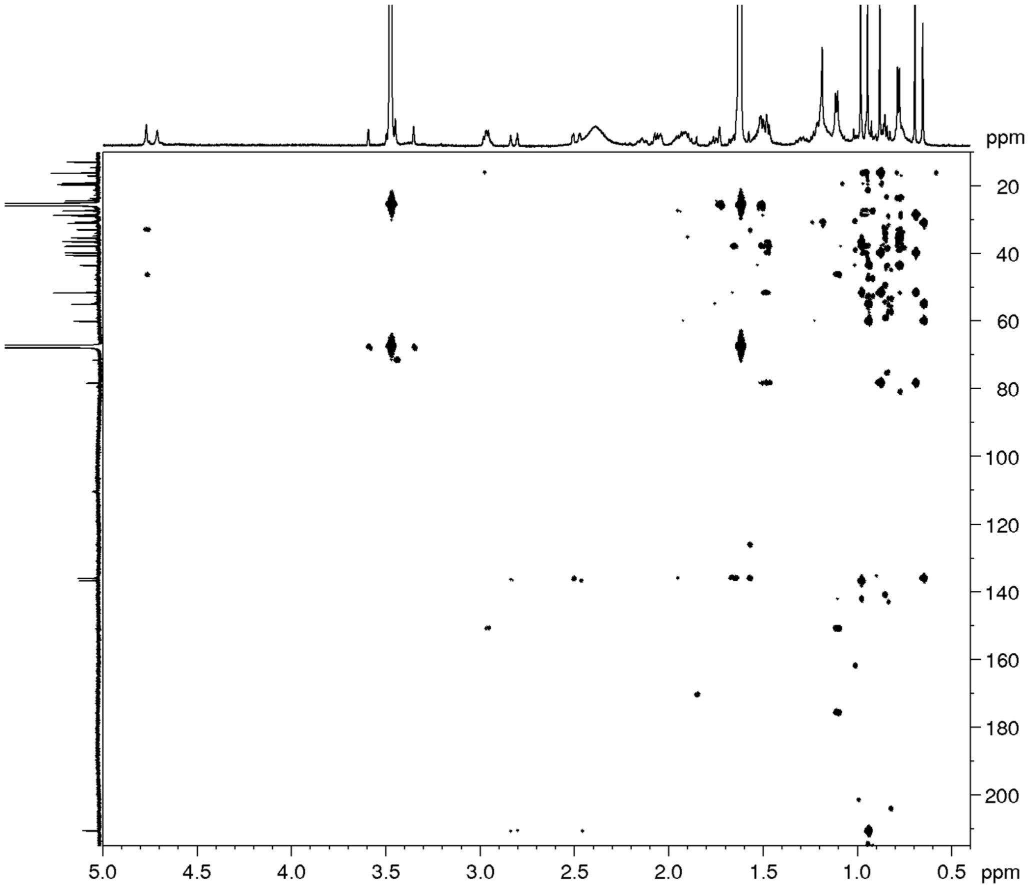

Figure S16. HMBC spectrum of compound **2** (600 MHz, THF-d_8, 295 K)

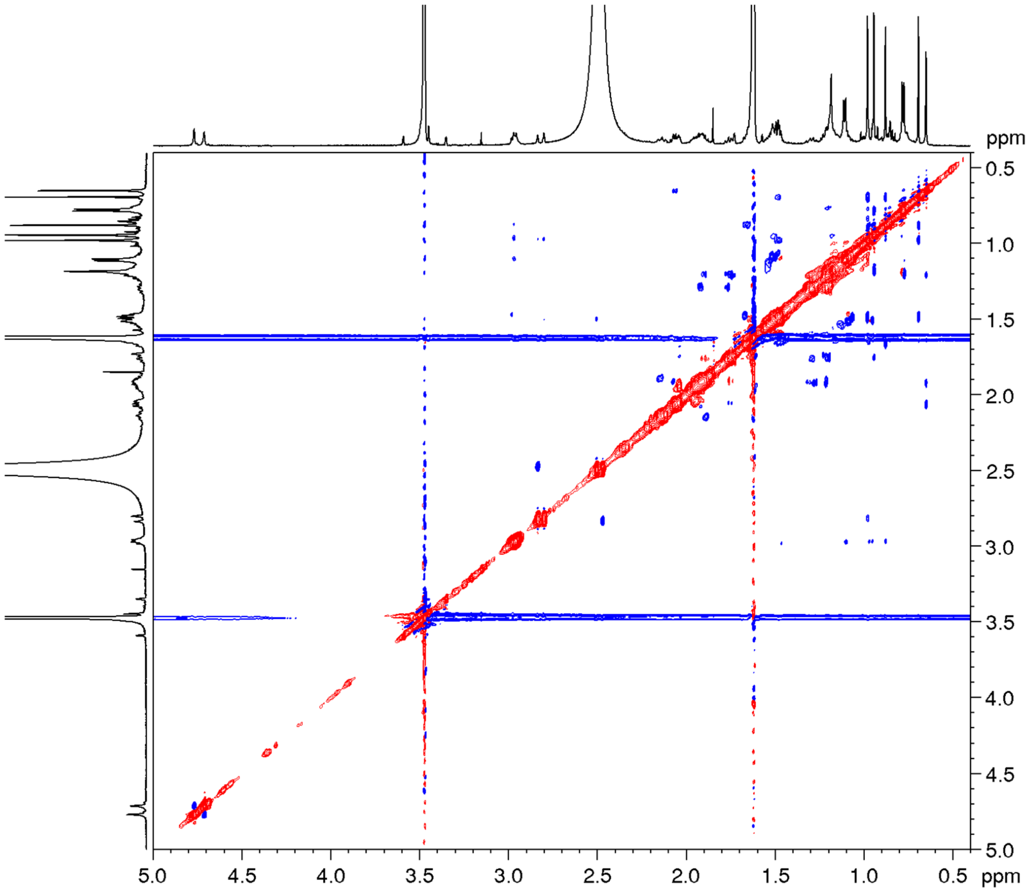

Figure S17. NOESY spectrum of compound **2** (600 MHz, THF-d_8, 295 K)

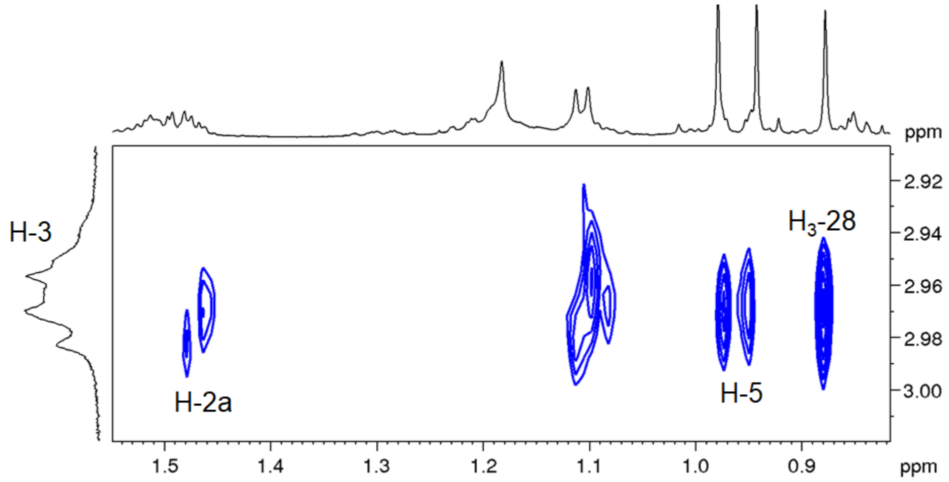

Figure S18. Selected region of the NOESY spectrum of compound **2** (600 MHz, THF-d_8, 295 K)

Spectra and spectral data on compound 3

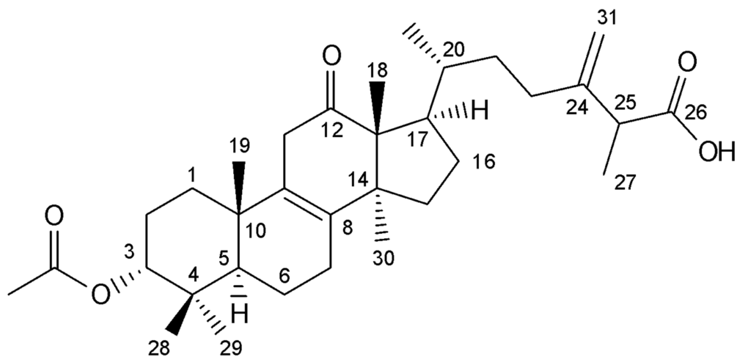

HR-ESI-MS (+) *m/z* 527.3721 [M + H]$^+$ (527.3731 calcd. for $C_{33}H_{51}O_5$; *Δ* -2.0 ppm); HR-ESI-MSMS (CID = 15%, 30%, 45%) 509.3611, 481.3671, 467.3504, 449.3400, 421.3456

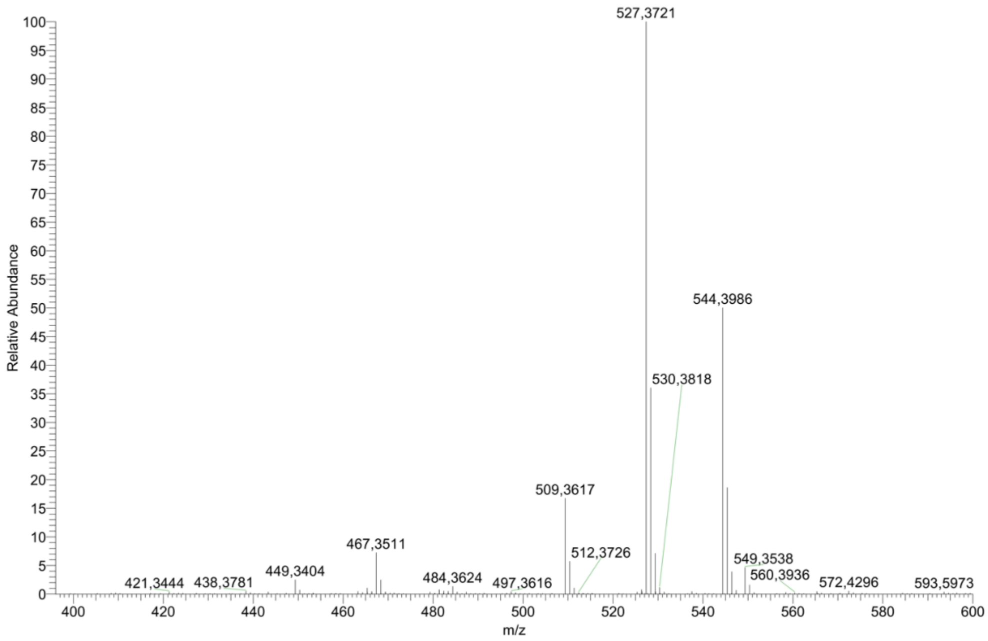

Figure S19. HR-ESI-MS spectrum of compound **3**

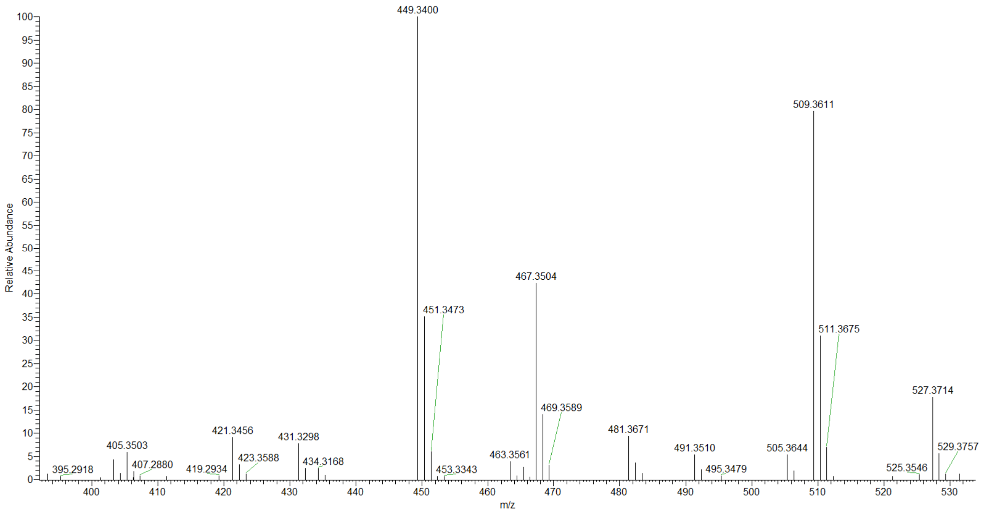

Figure S20. MS-MS spectrum of compound **3**

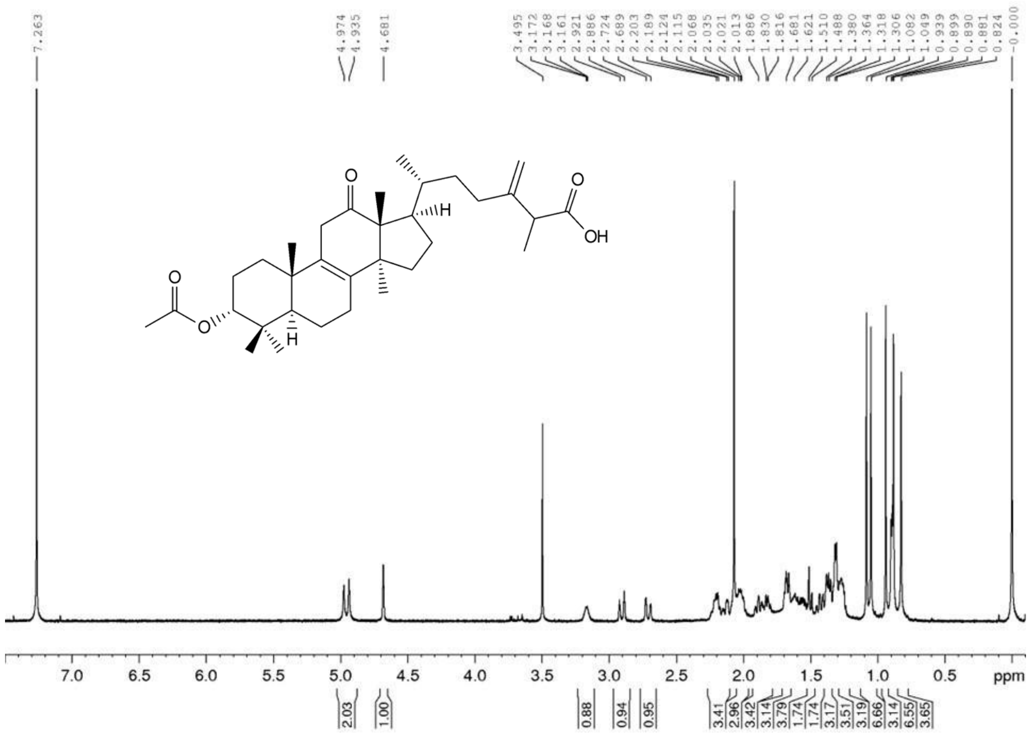

Figure S21. ^{1}H spectrum of compound **3** (600 MHz, $CDCl_3$, 295 K)

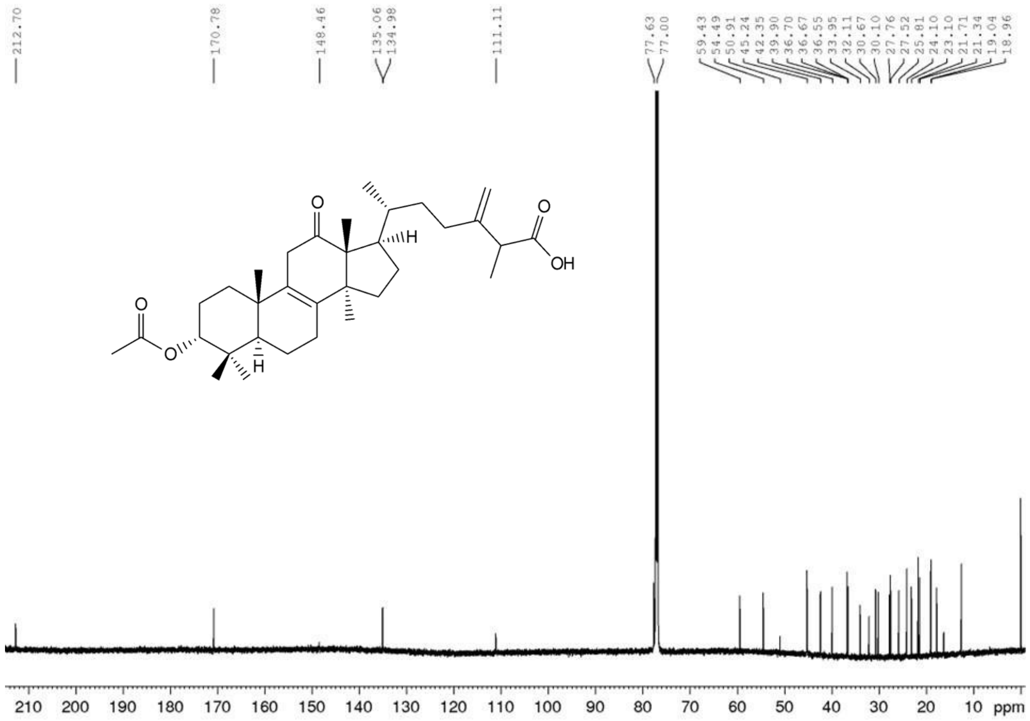

Figure S22. ^{13}C spectrum of compound **3** (150 MHz, $CDCl_3$, 295 K)

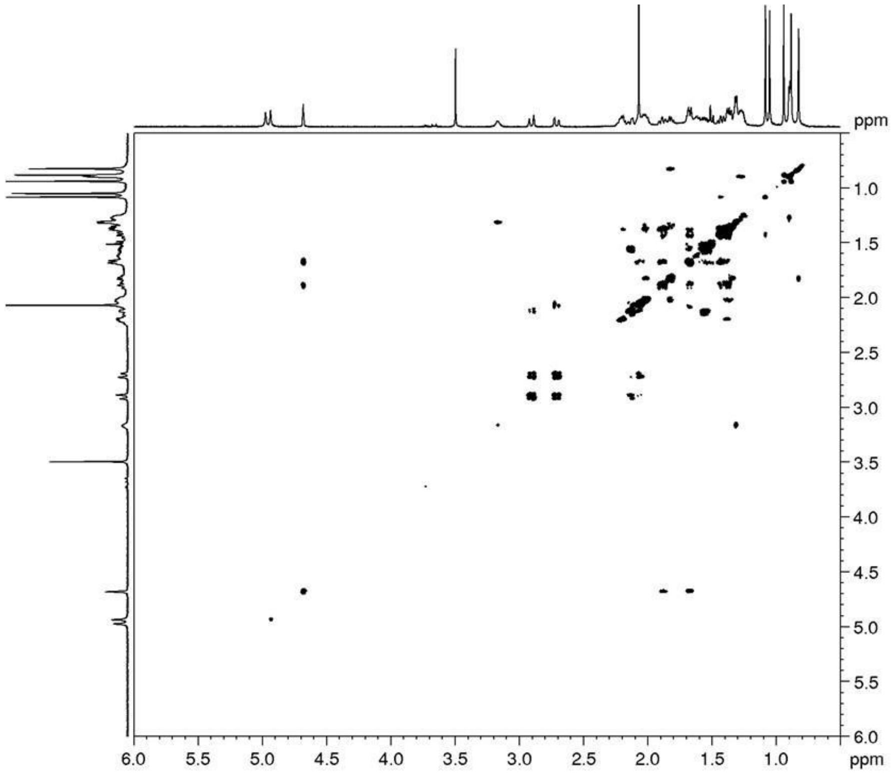

Figure S23. COSY spectrum of compound **3** (600 MHz, $CDCl_3$, 295 K)

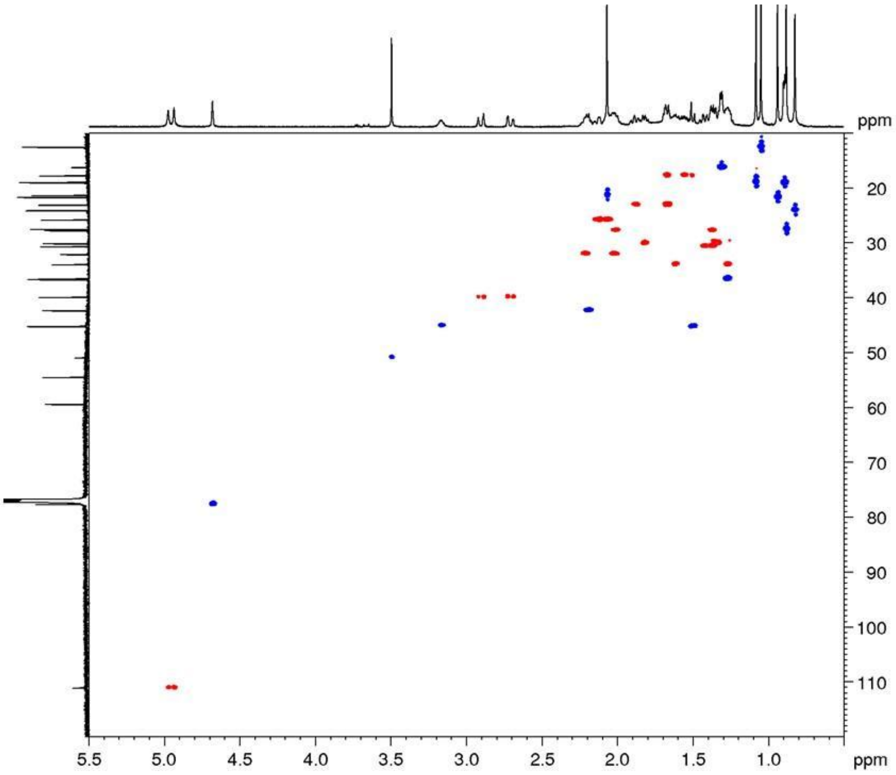

Figure S24. DEPT-edited HSQC spectrum of compound **3** (600 MHz, $CDCl_3$, 295 K)

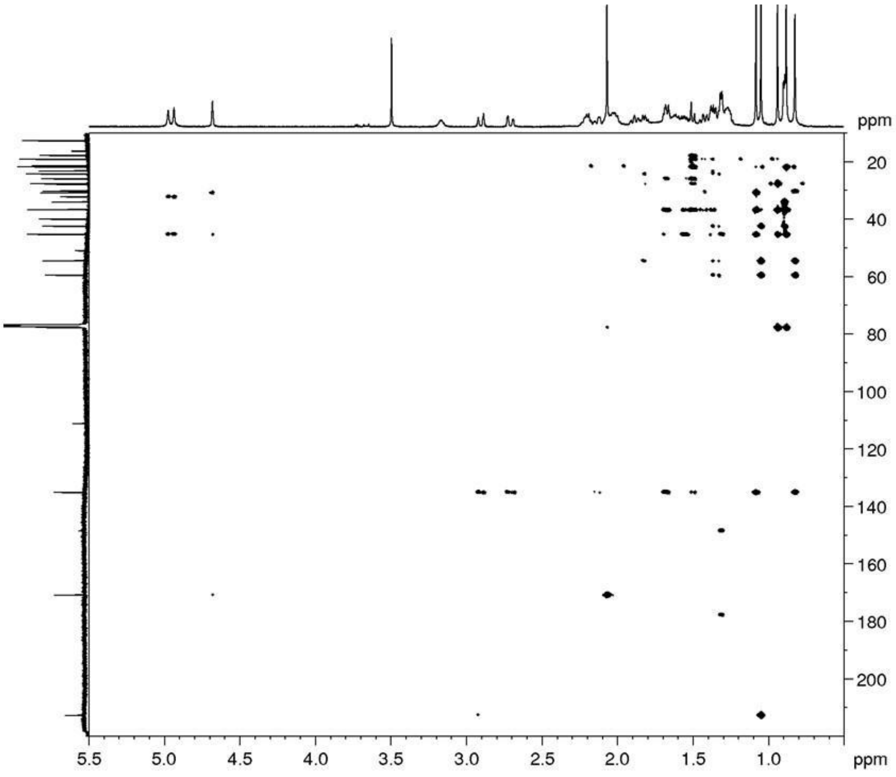

Figure S25. HMBC spectrum of compound **3** (600 MHz, $CDCl_3$, 295 K)

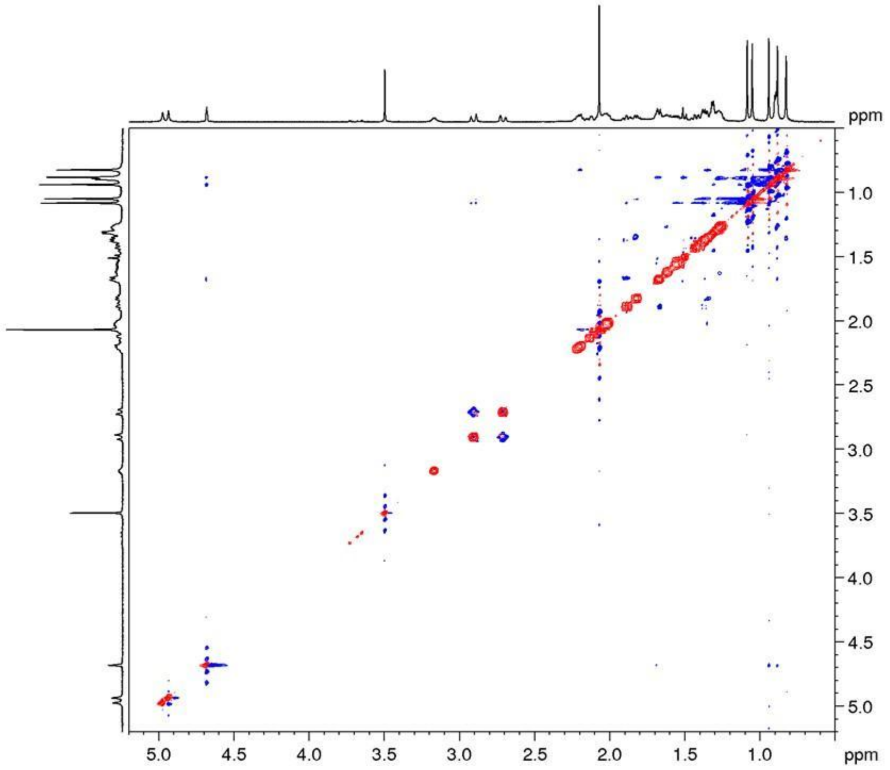

Figure S26. NOESY spectrum of compound **3** (600 MHz, $CDCl_3$, 295 K)

Spectra and spectral data on compound 4

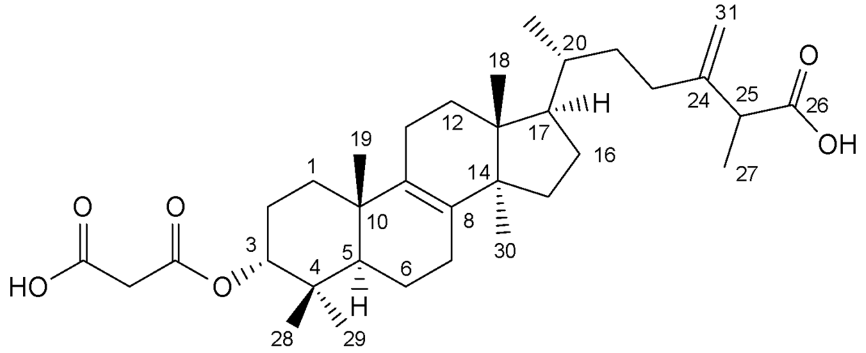

HR-ESI-MS (-) *m/z* 555.3694 [M − H]⁻ (555.3680 calcd. for $C_{34}H_{51}O_6$; *Δ* 2.6 ppm); HR-ESI-MSMS (CID = 15%, 30%, 45%) *m/z* 511.3787

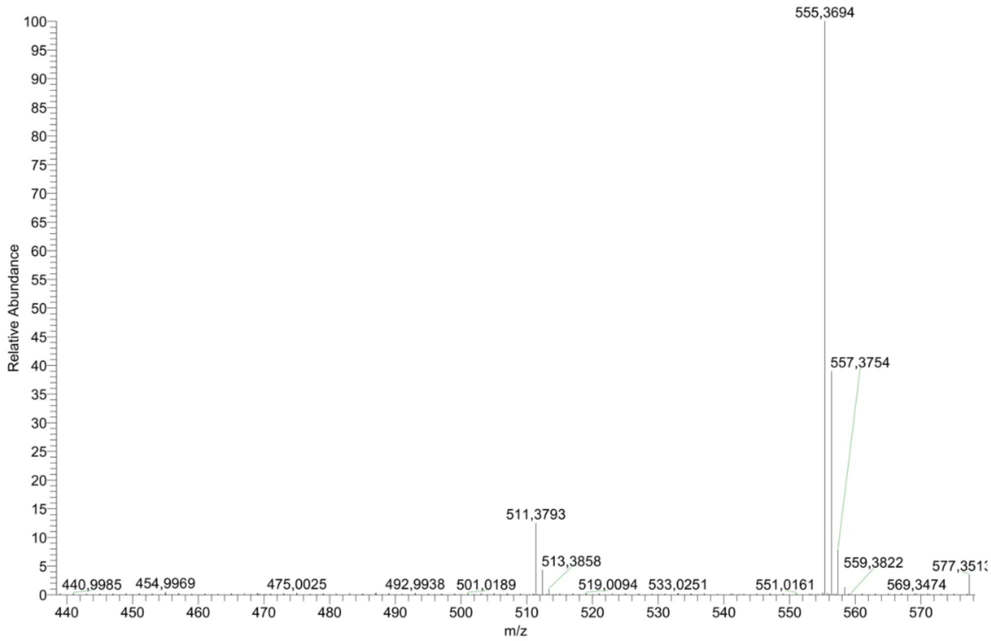

Figure S27. HR-ESI-MS spectrum of compound **4**

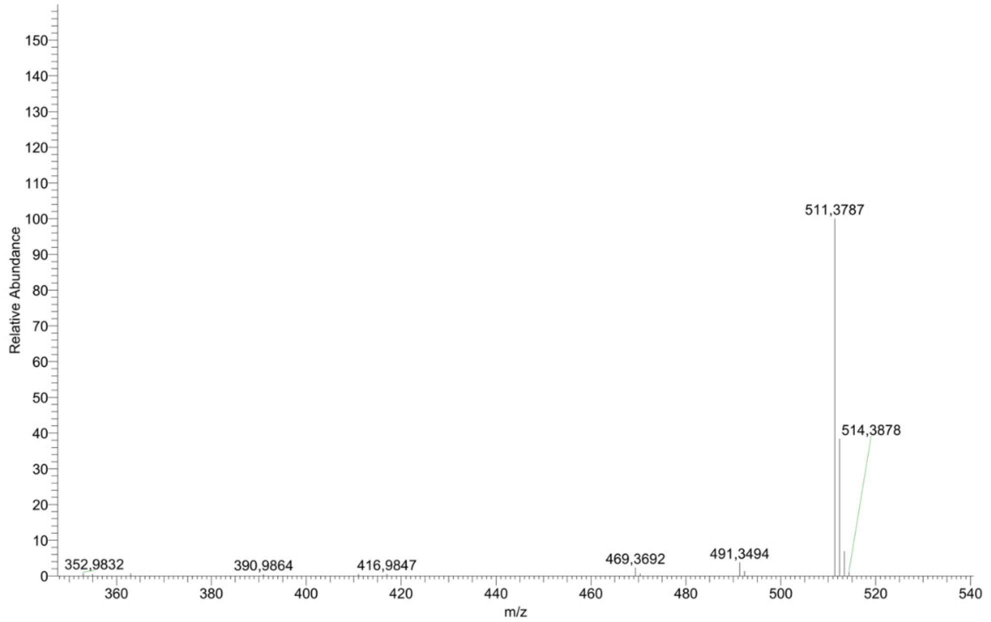

Figure S28. MS-MS spectrum of compound **4**

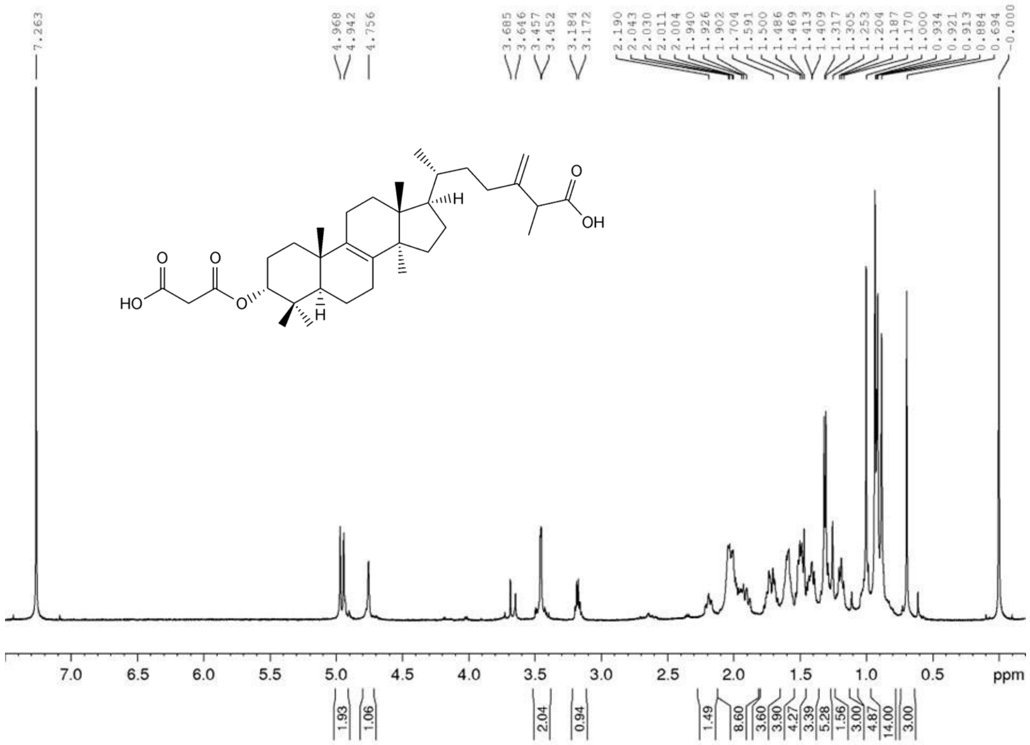

Figure S29. ^{1}H spectrum of compound **4** (600 MHz, $CDCl_3$, 295 K)

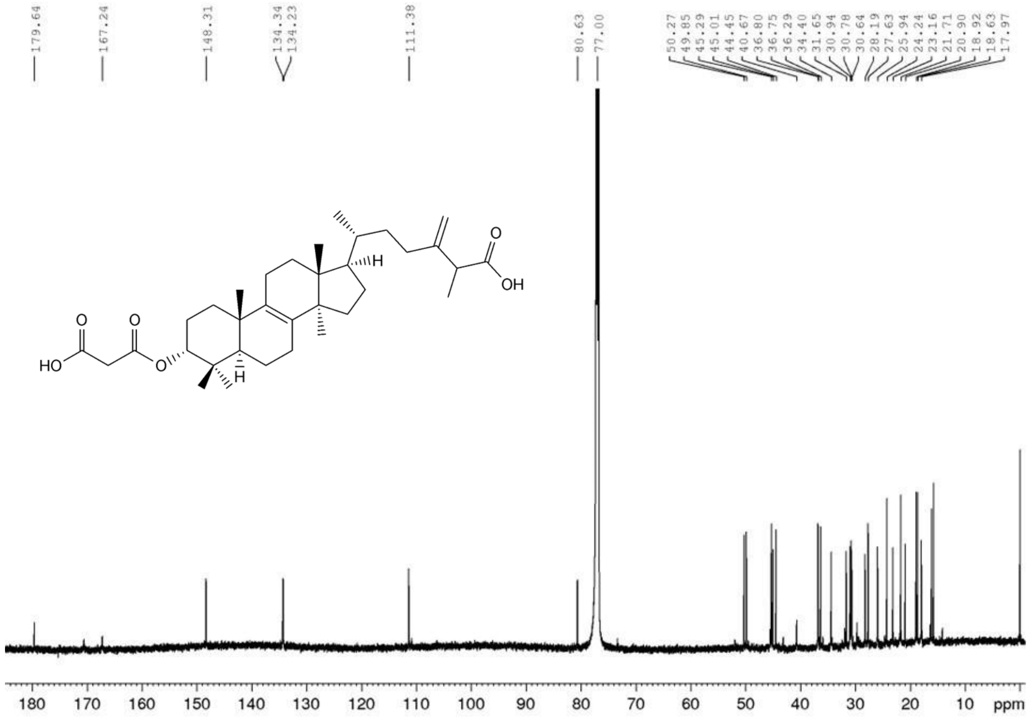

Figure S30. ^{13}C spectrum of compound **4** (150 MHz, $CDCl_3$, 295 K)

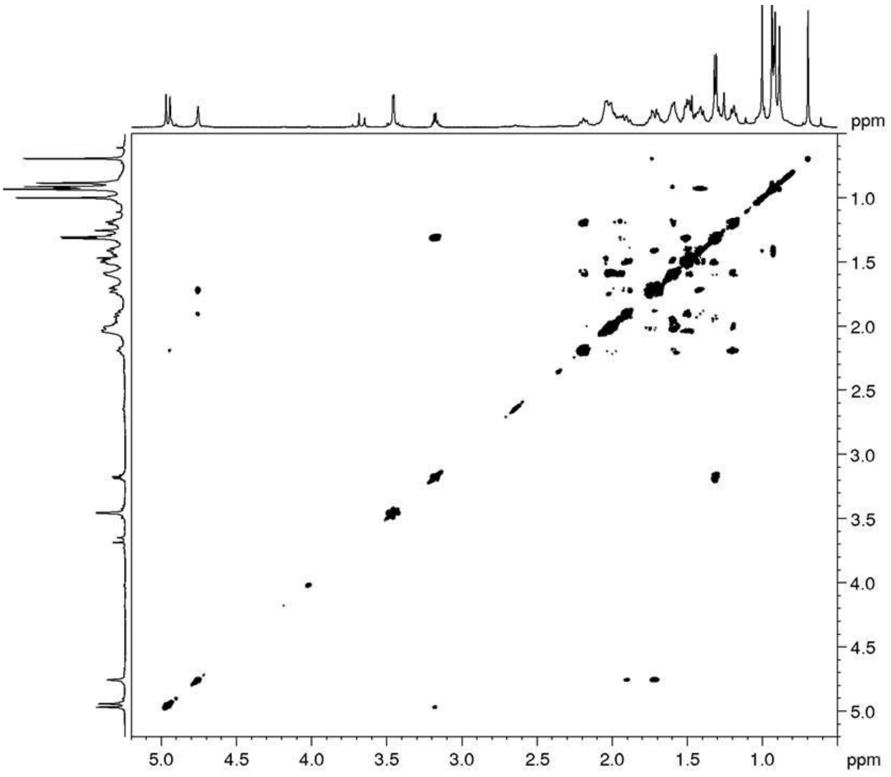

Figure S31. COSY spectrum of compound **4** (600 MHz, $CDCl_3$, 295 K)

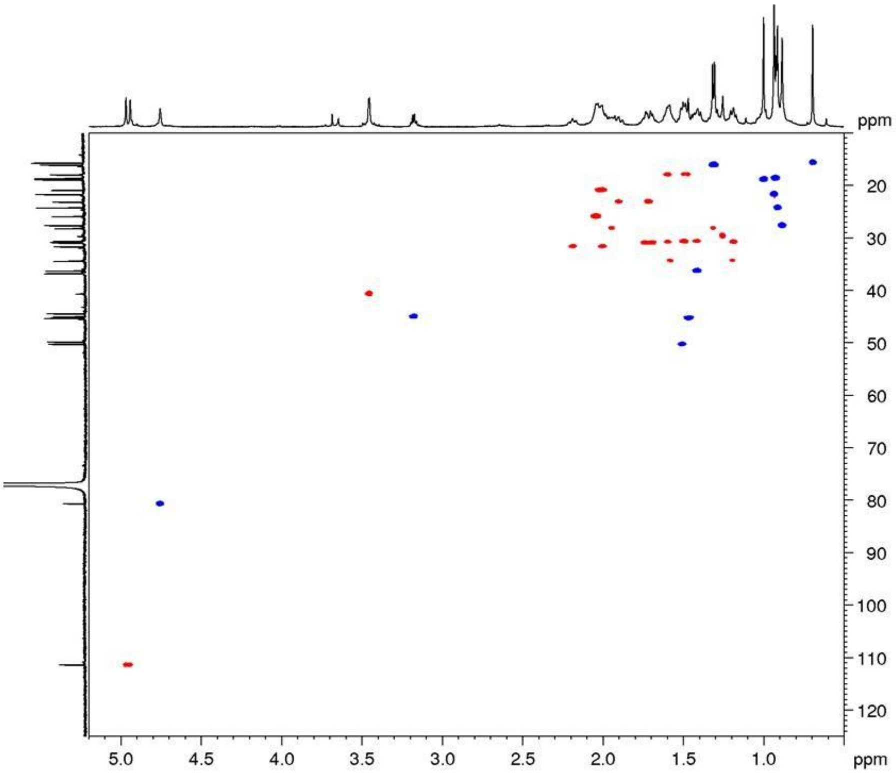

Figure S32. DEPT-edited HSQC spectrum of compound **4** (600 MHz, $CDCl_3$, 295 K)

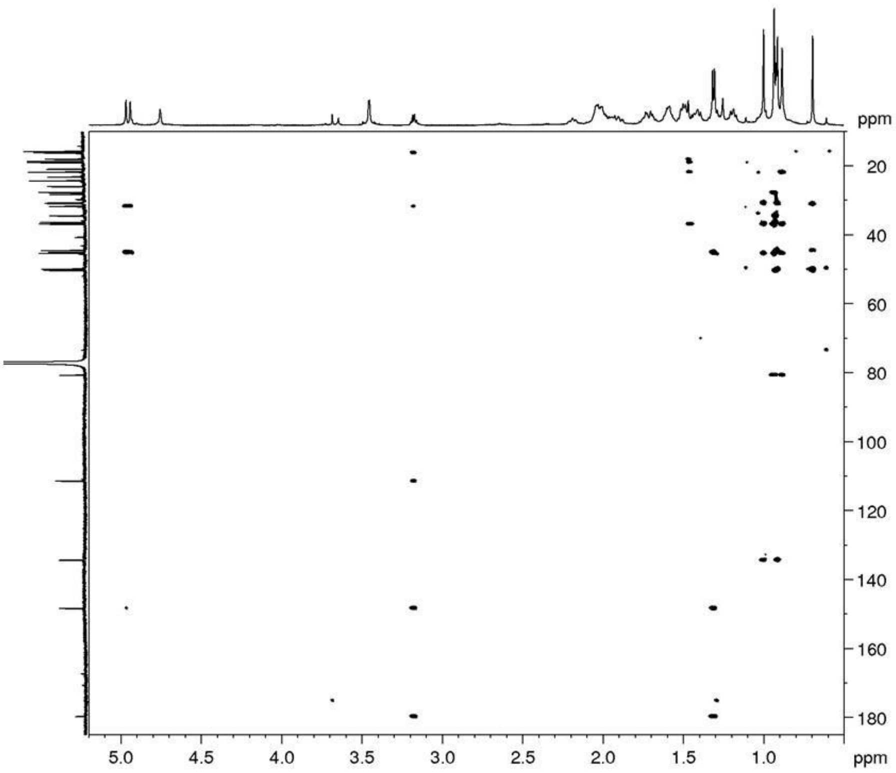

Figure S33. HMBC spectrum of compound **4** (600 MHz, $CDCl_3$, 295 K)

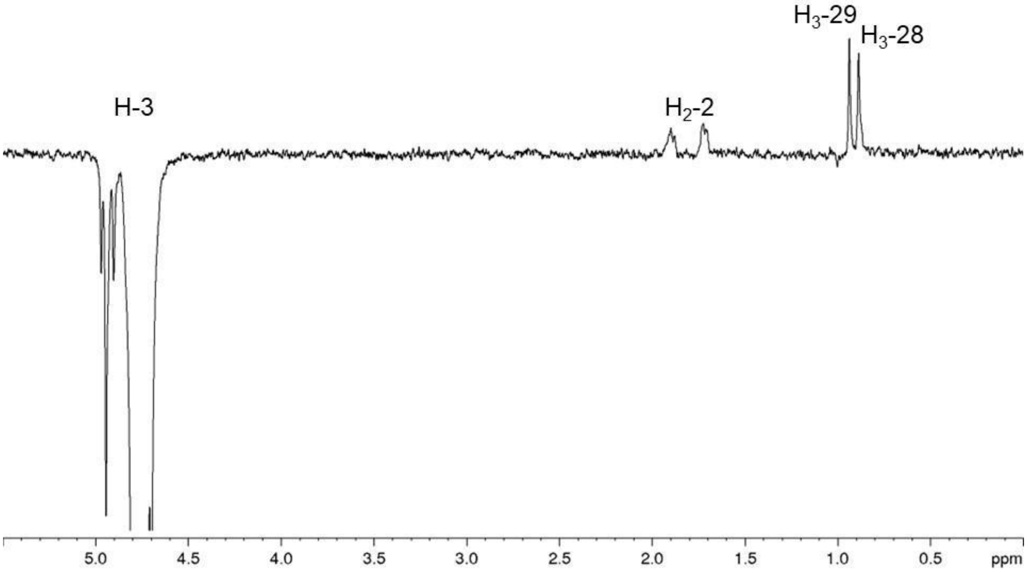

Figure S34. Selective gradient NOESY spectrum of compound **4** (600 MHz, $CDCl_3$, 295 K)

Spectra and spectral data on compound 5

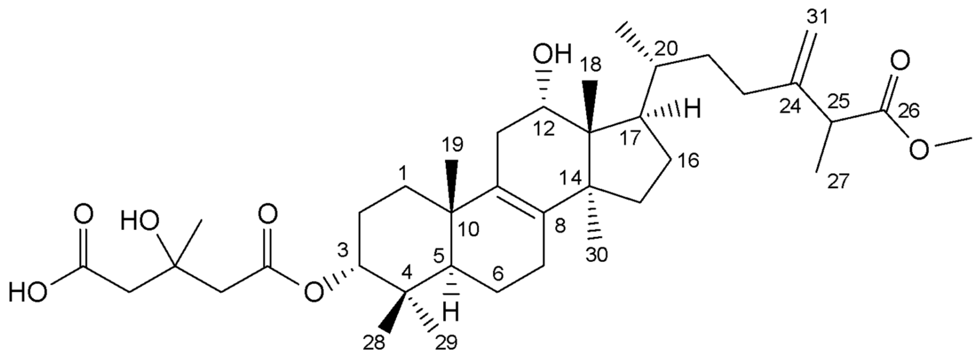

HR-ESI-MS (+) *m/z* 645.4343 [M + H]$^+$ (645.4361 calcd. for $C_{38}H_{61}O_8$; *Δ* 2.8 ppm); HR-ESI-MSMS (CID = 15%, 30%, 45%) 627.4243, 465.3715

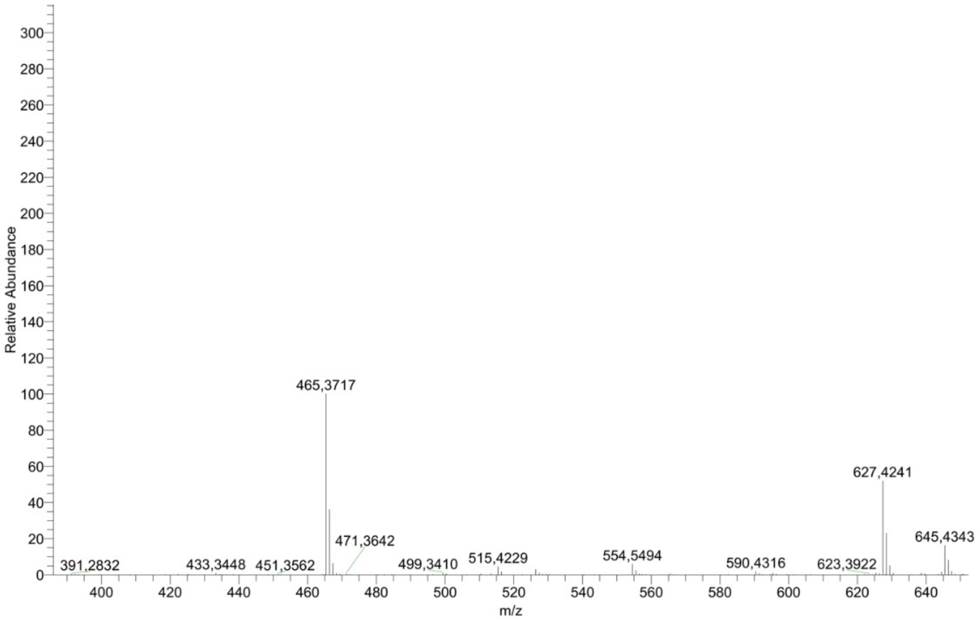

Figure S35. HR-ESI-MS spectrum of compound **5**

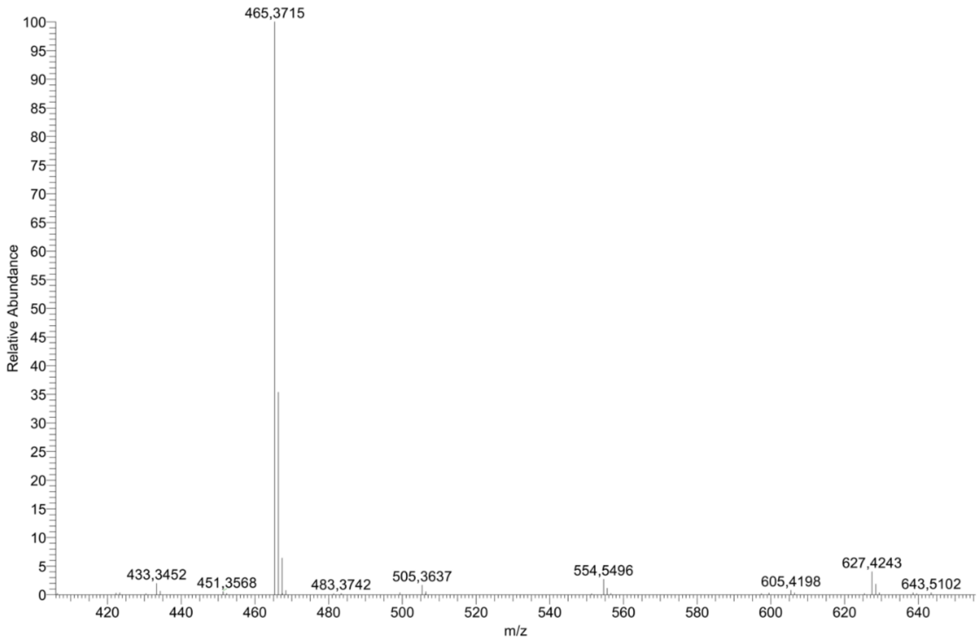

Figure S36. MS-MS spectrum of compound **5**

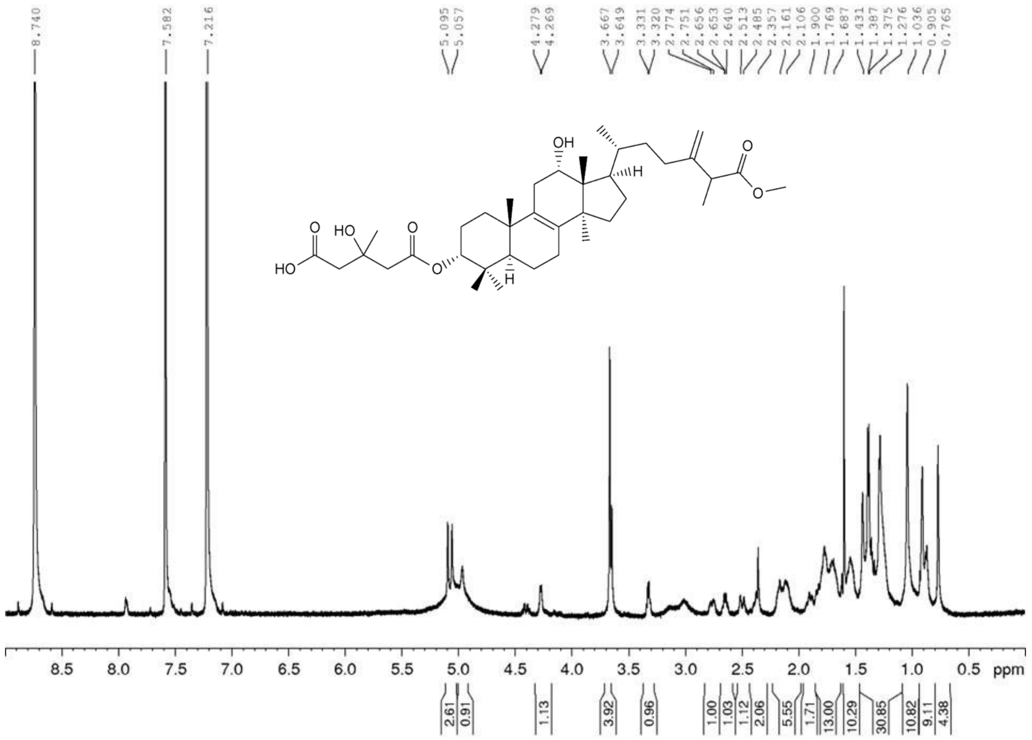

Figure S37. ^{1}H spectrum of compound **5** (600 MHz, pyridine-d_5, 295 K)

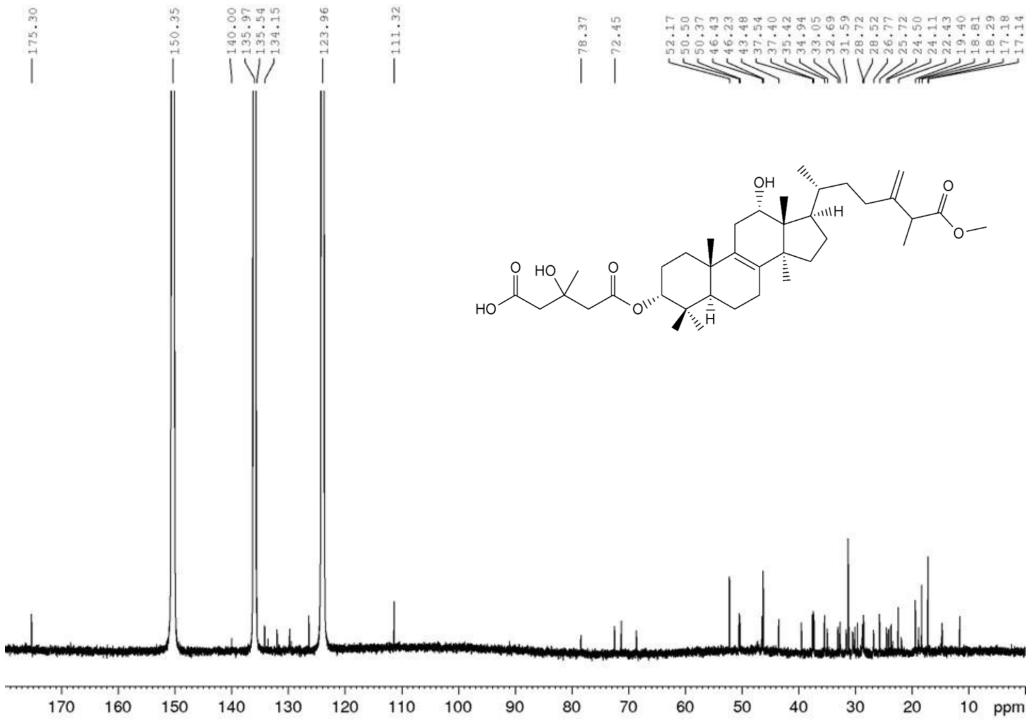

Figure S38. **^{13}C** spectrum of compound **5** (150 MHz, pyridine-d_5, 295 K)

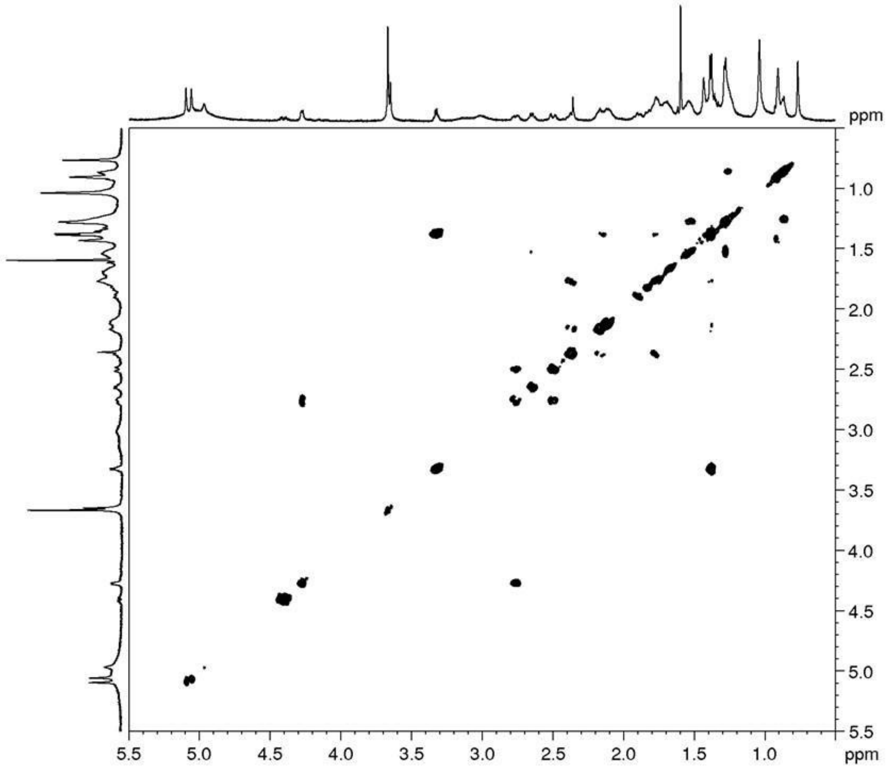

Figure S39. COSY spectrum of compound **5** (600 MHz, pyridine-d_5, 295 K)

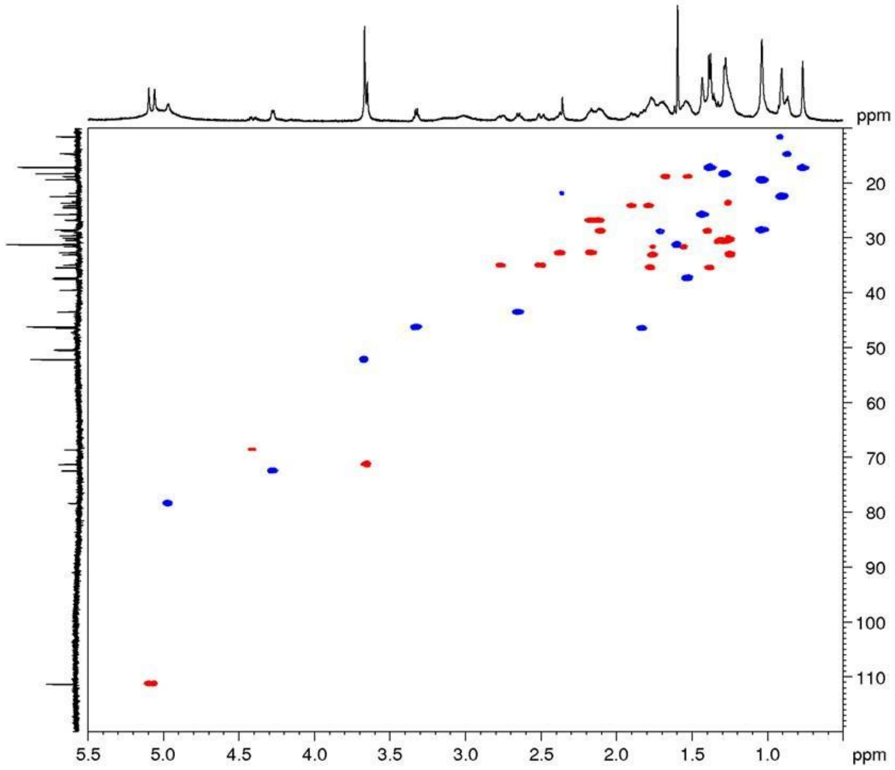

Figure S40. DEPT-edited HSQC spectrum of compound **5** (600 MHz, pyridine-d_5, 295 K)

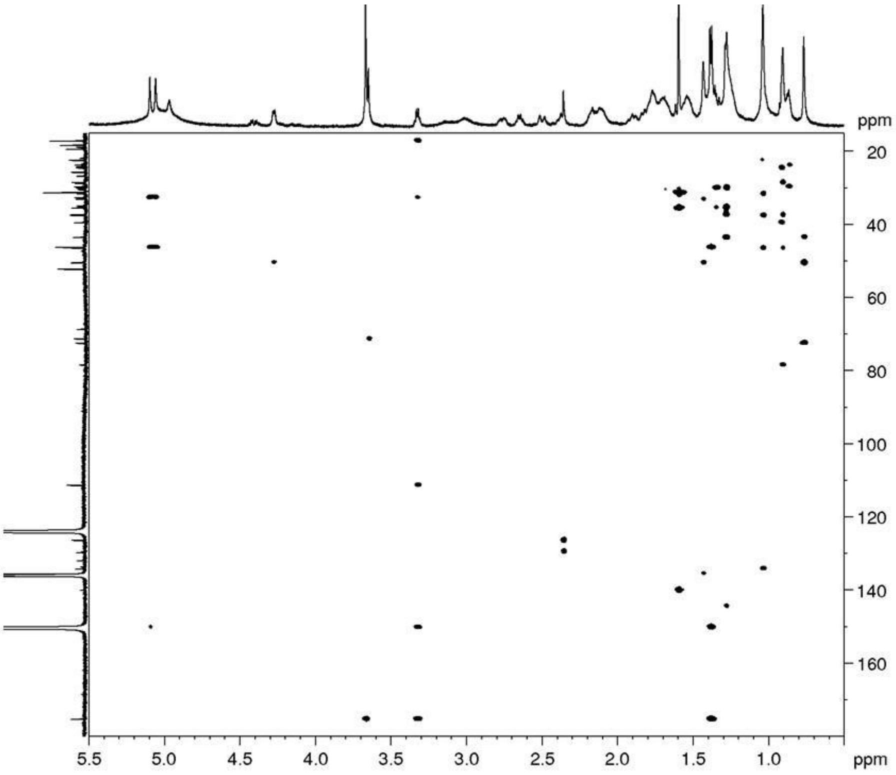

Figure S41. HMBC spectrum of compound **5** (600 MHz, pyridine-d_5, 295 K)

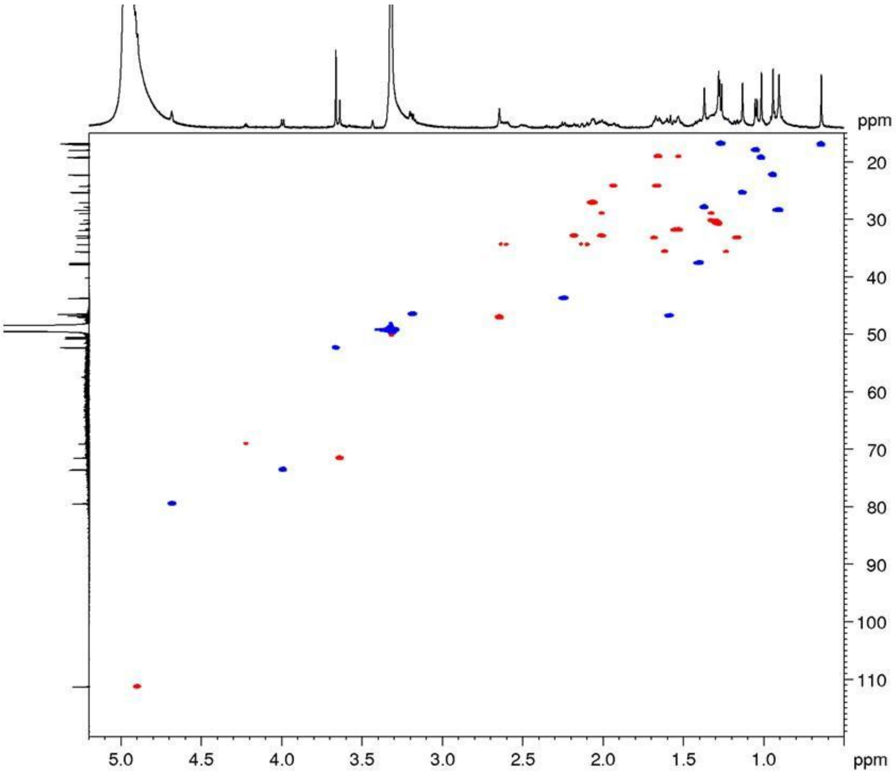

Figure S42. DEPT-edited HSQC spectrum of compound **5** (600 MHz, CD_3OD:pyridine-d_5 (19:1), 295 K)

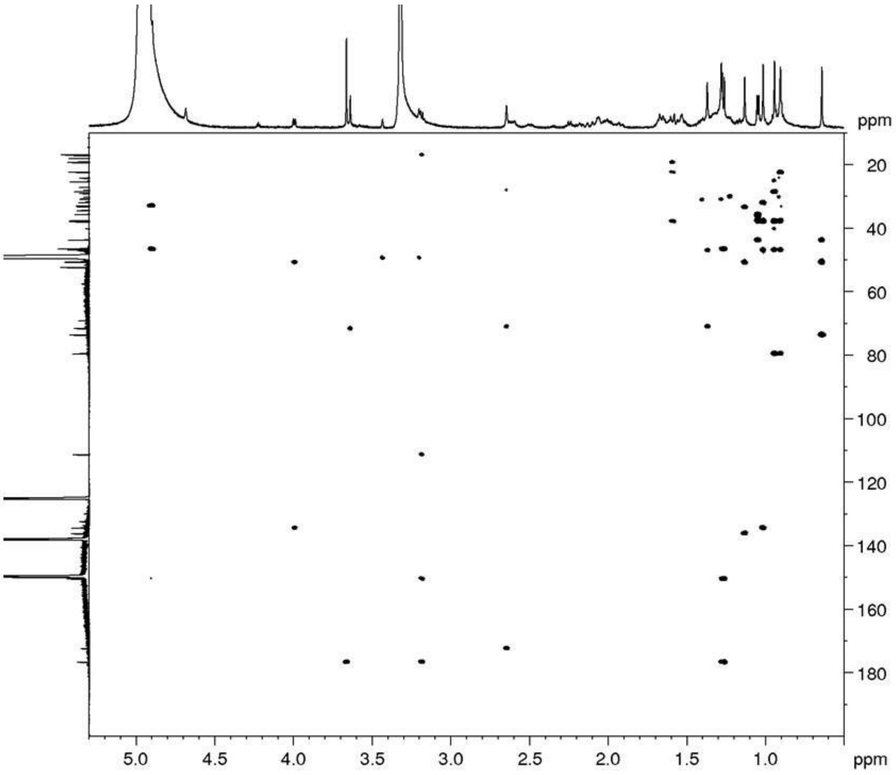

Figure S43. HMBC spectrum of compound **5** (600 MHz, CD_3OD: pyridine-d_5 (19:1), 295 K)

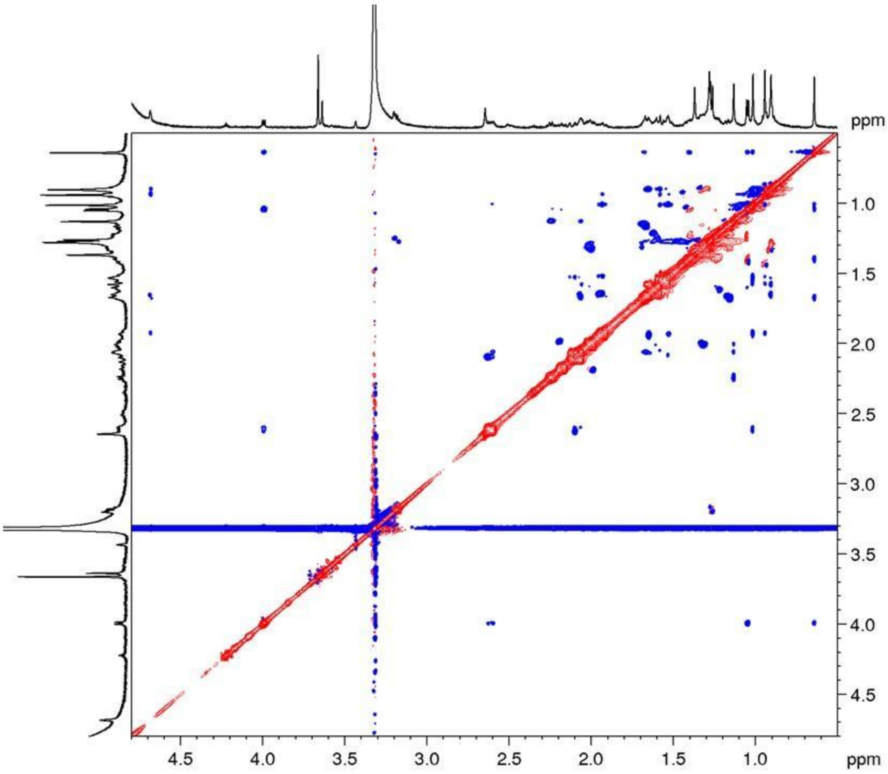

Figure S44. ROESY spectrum of compound **5** (600 MHz, CD_3OD:pyridine-d_5 (19:1), 295 K)

Spectra and spectral data on compound 6

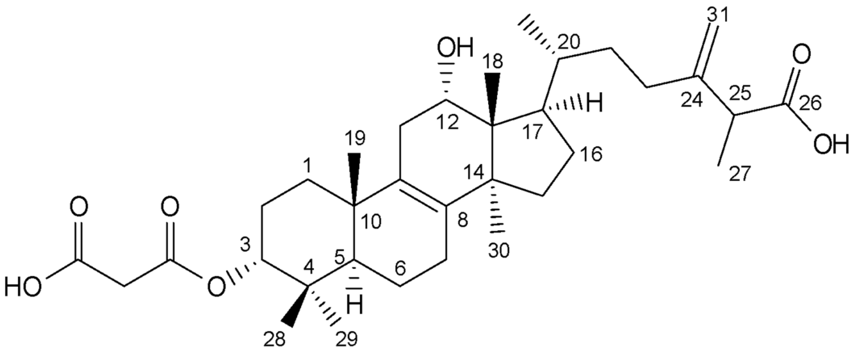

HR-ESI-MS (-) *m/z* 571.3642 [M - H]$^-$ (571.3629 calcd. for $C_{34}H_{51}O_7$; Δ 2.2 ppm;); HR-ESI-MSMS (CID = 15%, 30%, 45%) 527.3740, 483.3840, 441.3737

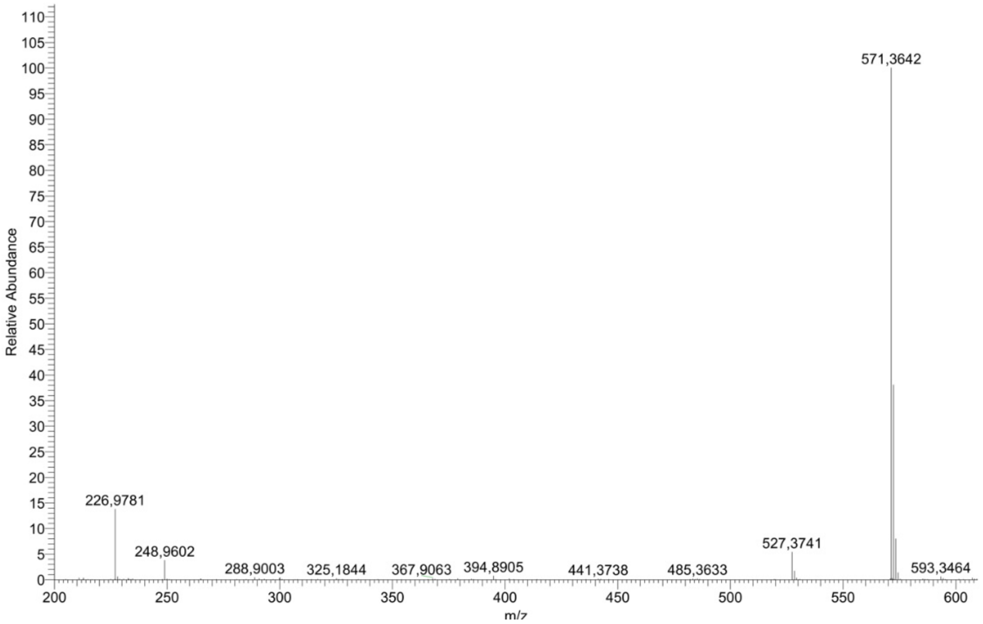

Figure S45. HR-ESI-MS spectrum of compound **6**

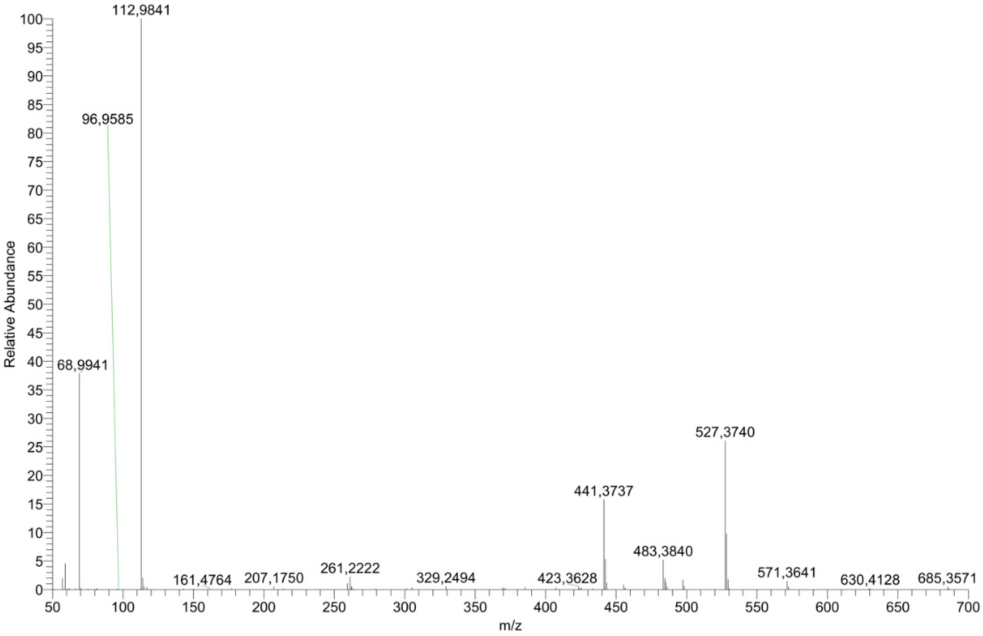

Figure S46. MS-MS spectrum of compound **6**

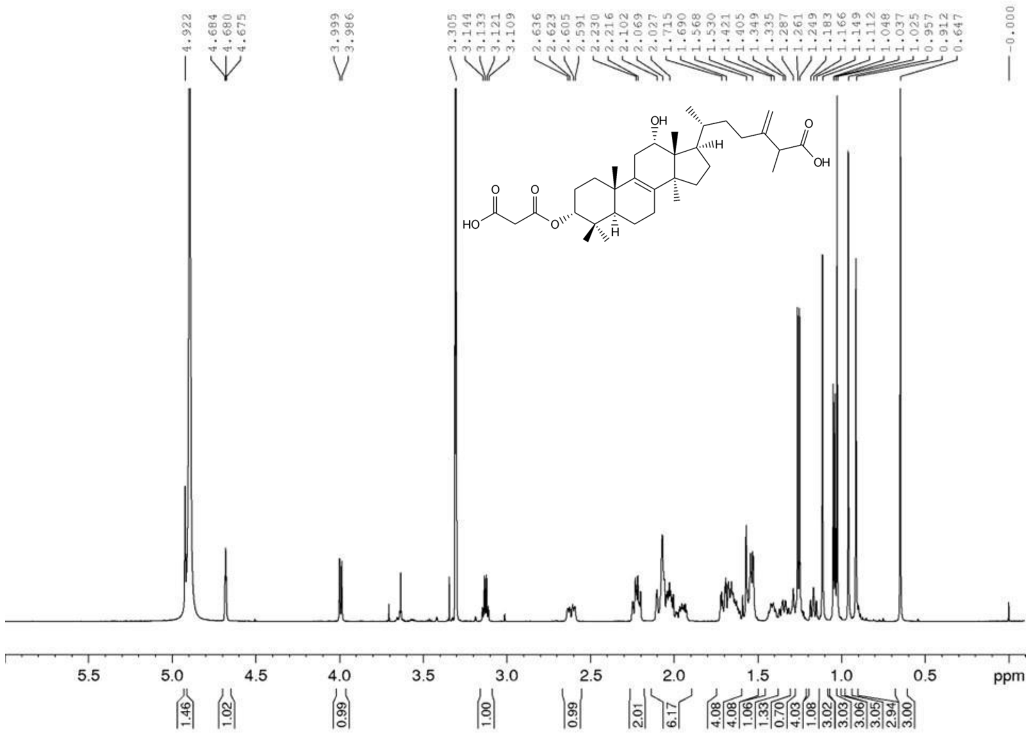

Figure S47. ^{1}H spectrum of compound **6** (150 MHz, CD_3OD, 295 K)

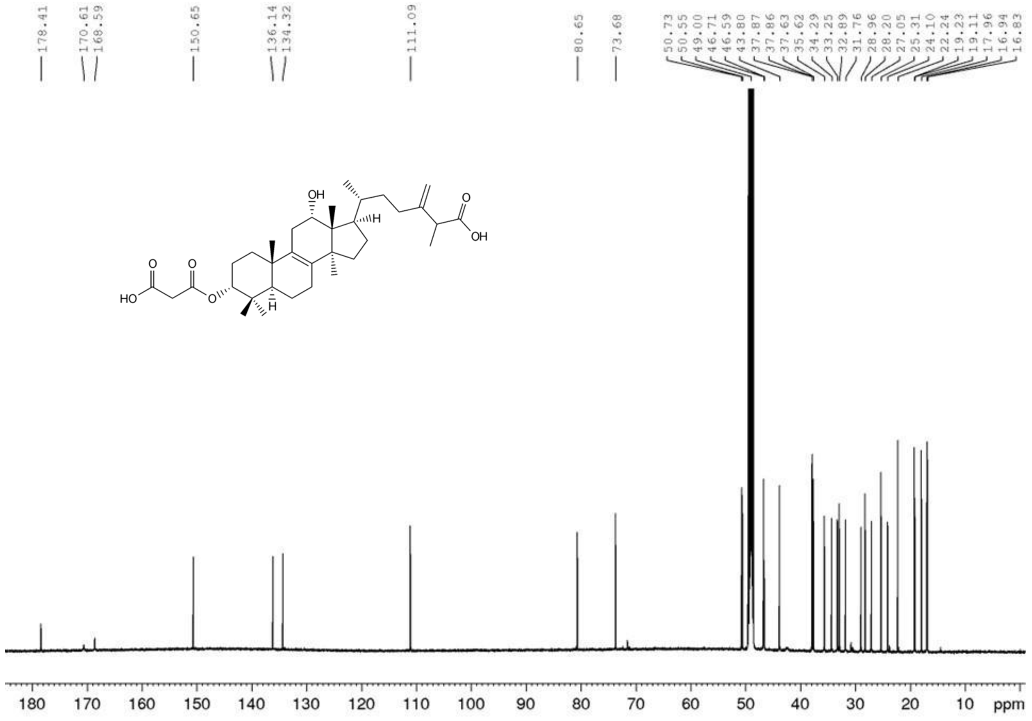

Figure S48. ^{13}C spectrum of compound **6** (600 MHz, CD_3OD, 295 K)

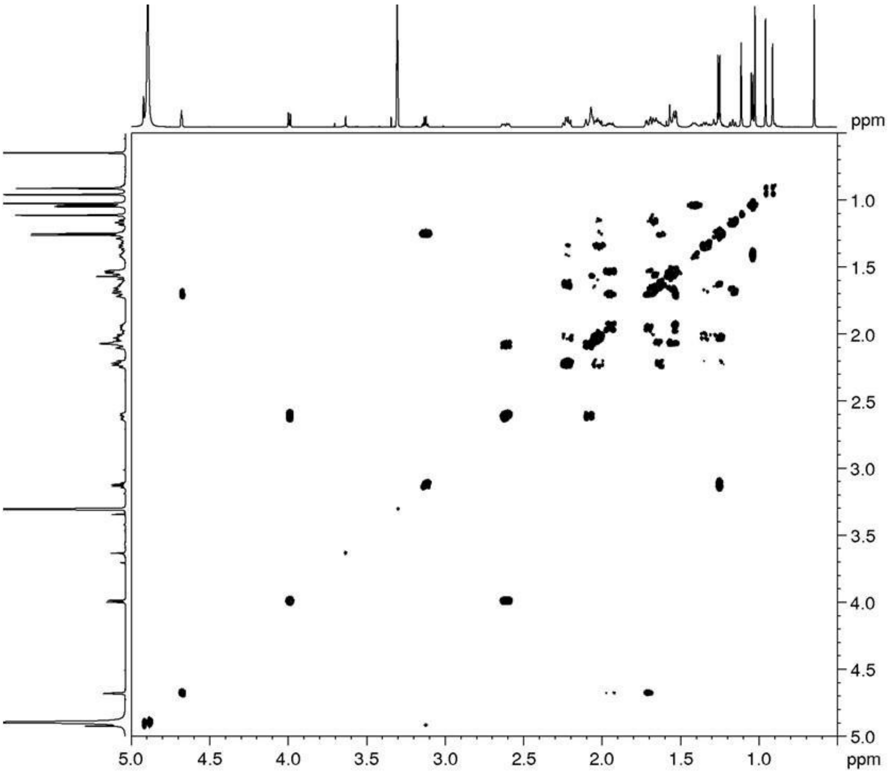

Figure S49. COSY spectrum of compound **6** (600 MHz, CD_3OD, 295 K)

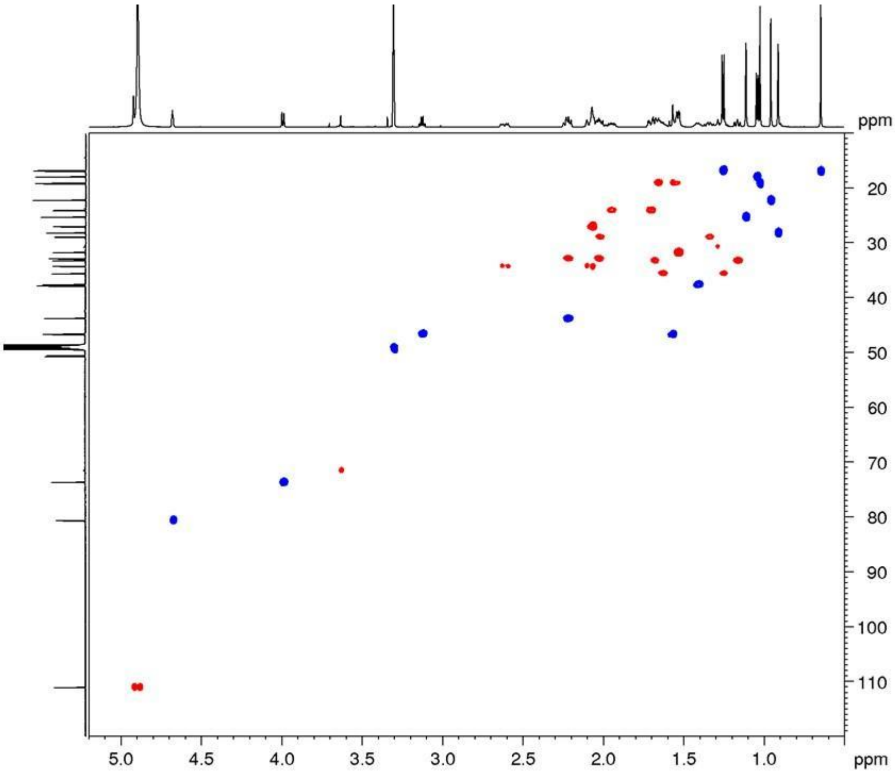

Figure S50. DEPT-edited HSQC spectrum of compound **6** (600 MHz CD_3OD, 295 K)

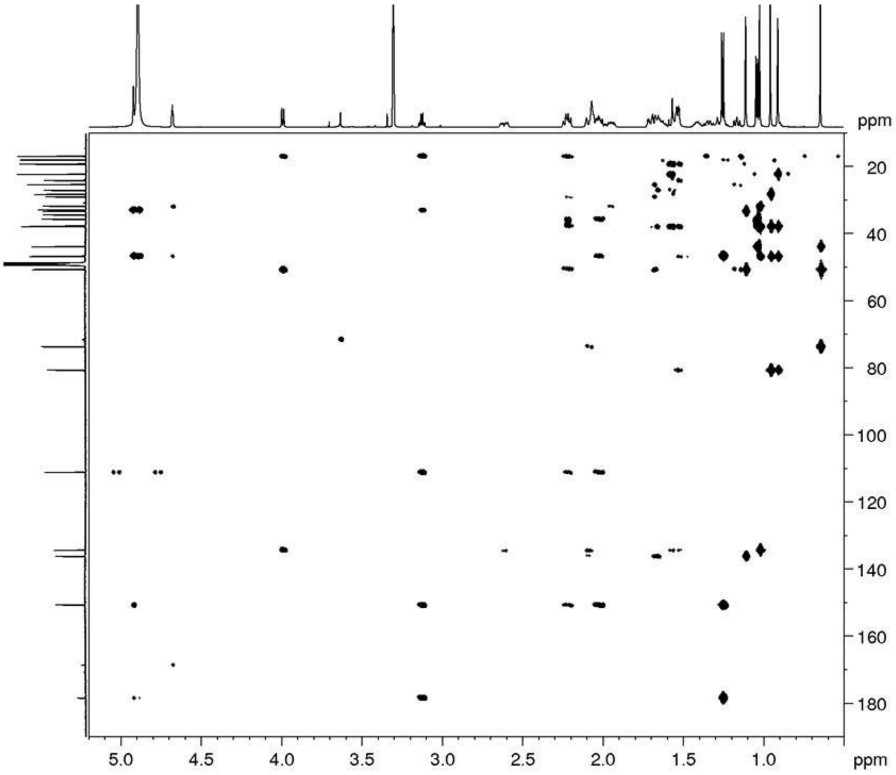

Figure S51. HMBC spectrum of compound **6** (600 MHz, CD_3OD, 295 K)

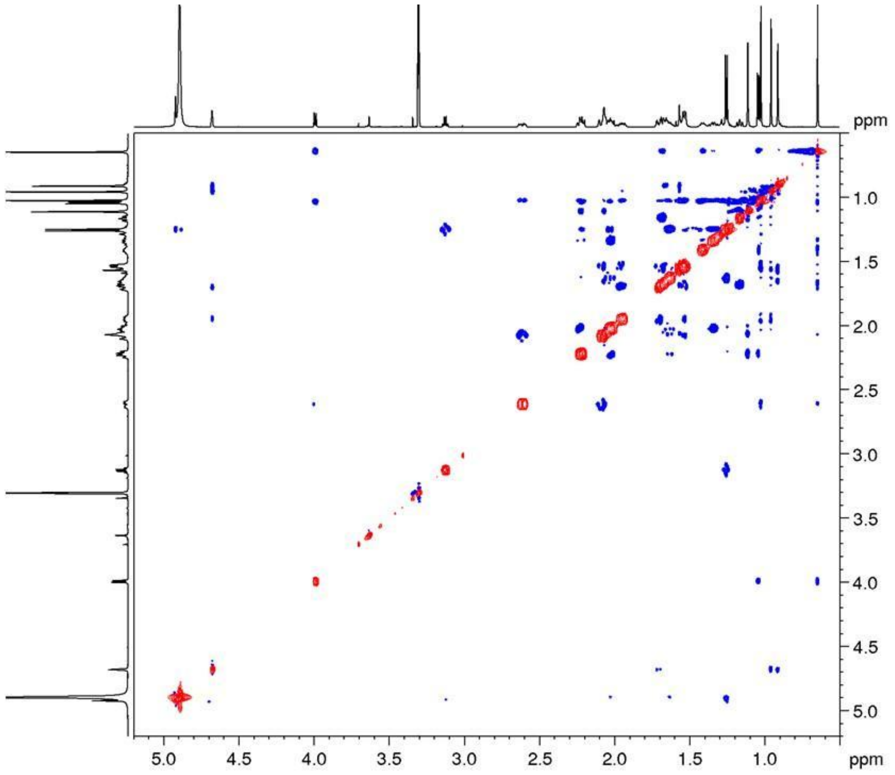

Figure S52. NOESY spectrum of compound **6** (600 MHz, CD_3OD, 295 K)

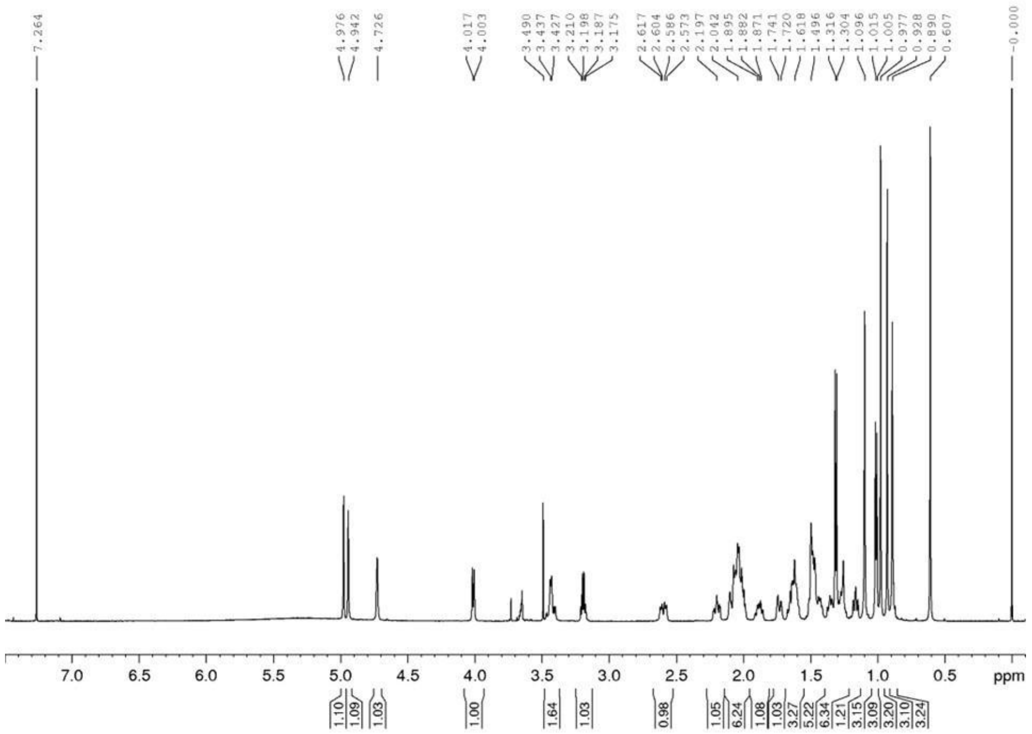

Figure S53. ^{1}H spectrum of compound **6** (600 MHz, $CDCl_3$, 295 K)

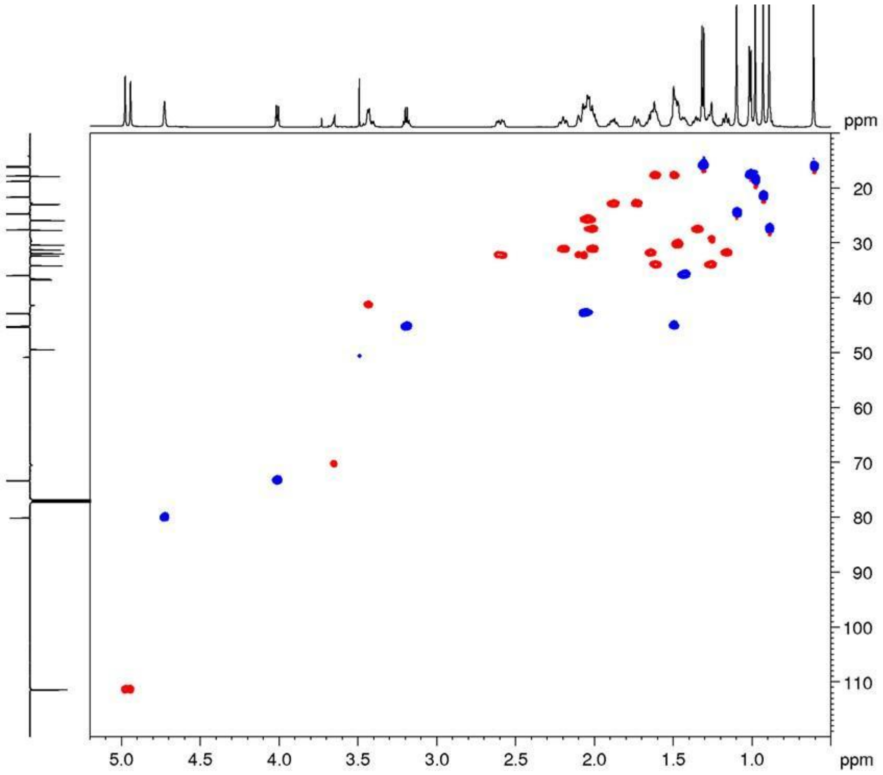

Figure S54. DEPT-edited HSQC spectrum of compound **6** (600 MHz, $CDCl_3$, 295 K)

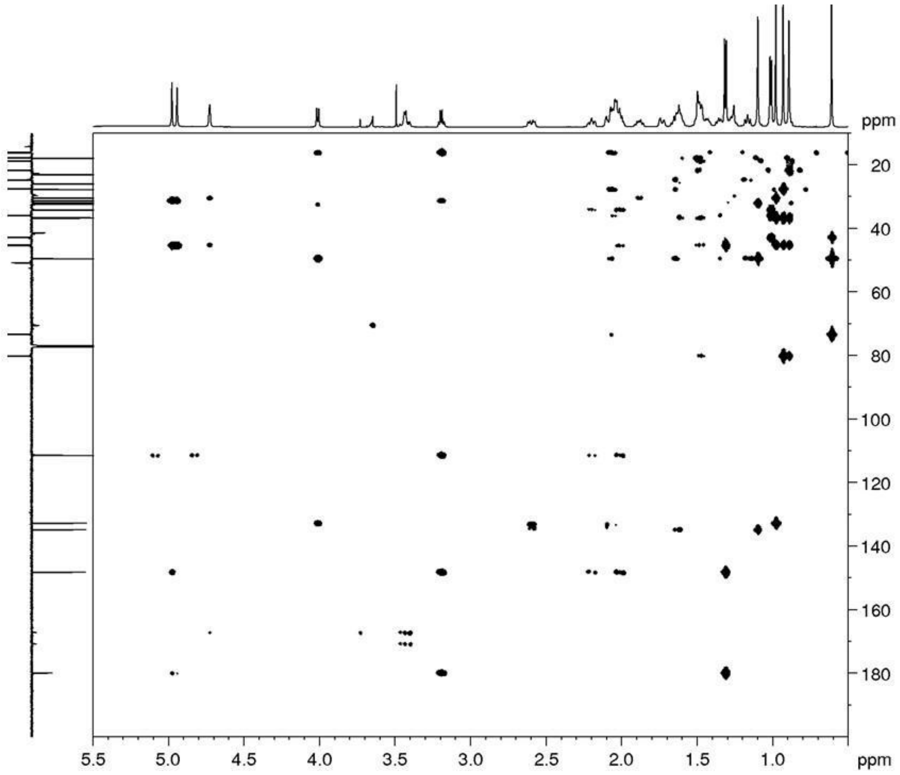

Figure S55. HMBC spectrum of compound **6** (600 MHz, $CDCl_3$, 295 K)

Spectra and spectral data on compound 7

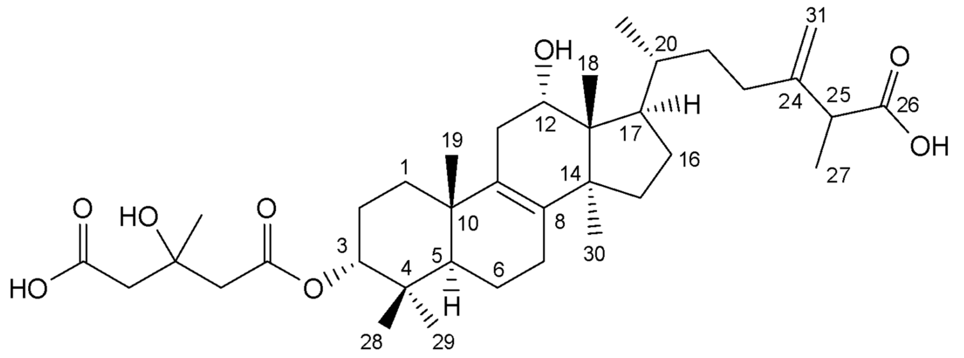

HR-ESI-MS (-) *m/z* 629.4063 [M - H]$^{-}$ (629.4048 calcd. for $C_{37}H_{57}O_8$ Δ 2.3 ppm;); HR-ESI-MSMS (CID = 15%, 30%, 45%) 567.4055, 527.3735, 485.3621, 483 (27), 441.3735

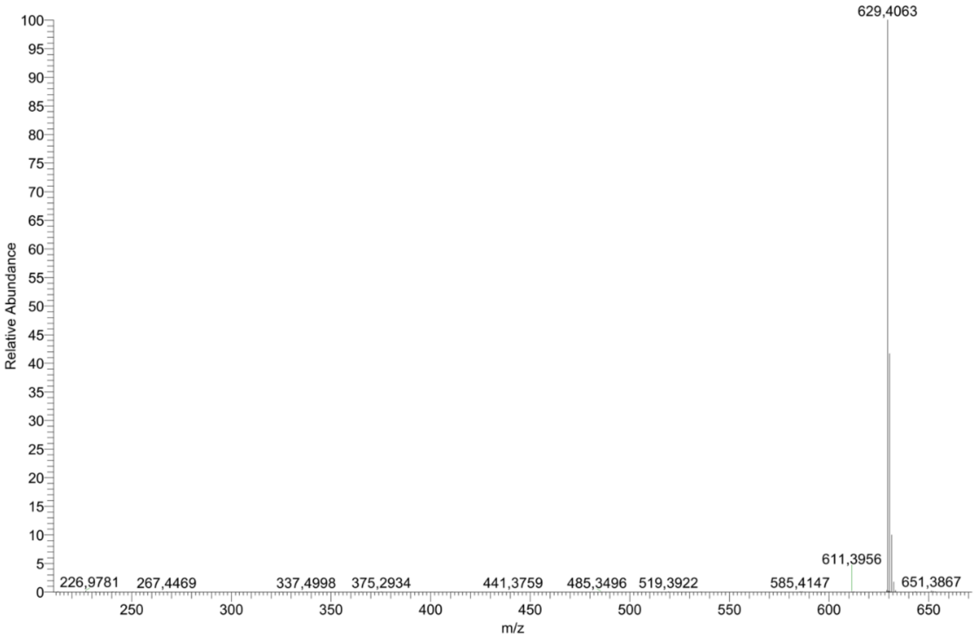

Figure S56. HR-ESI-MS spectrum of compound **7**

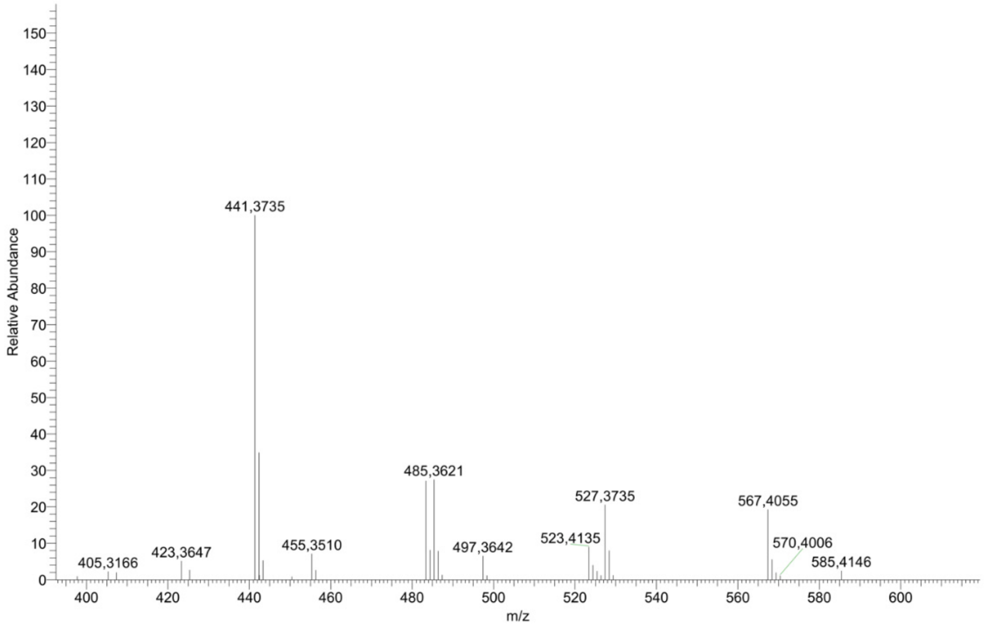

Figure S57. MS-MS spectrum of compound **7**

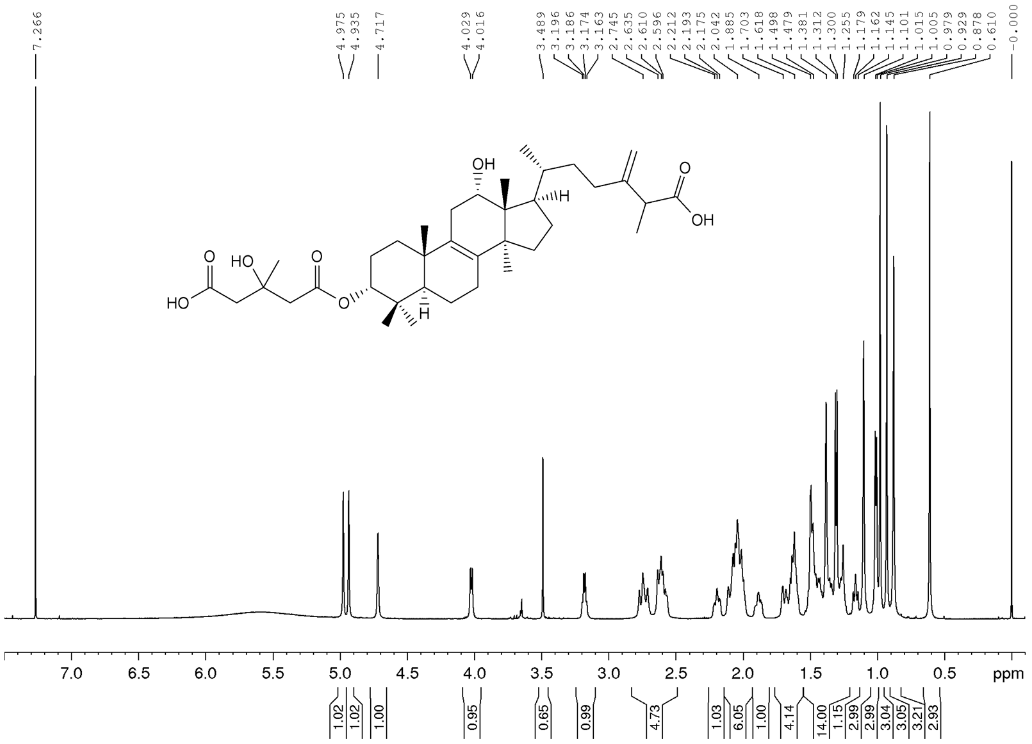

Figure S58. ^{1}H spectrum of compound **7** (600 MHz, $CDCl_3$, 295 K)

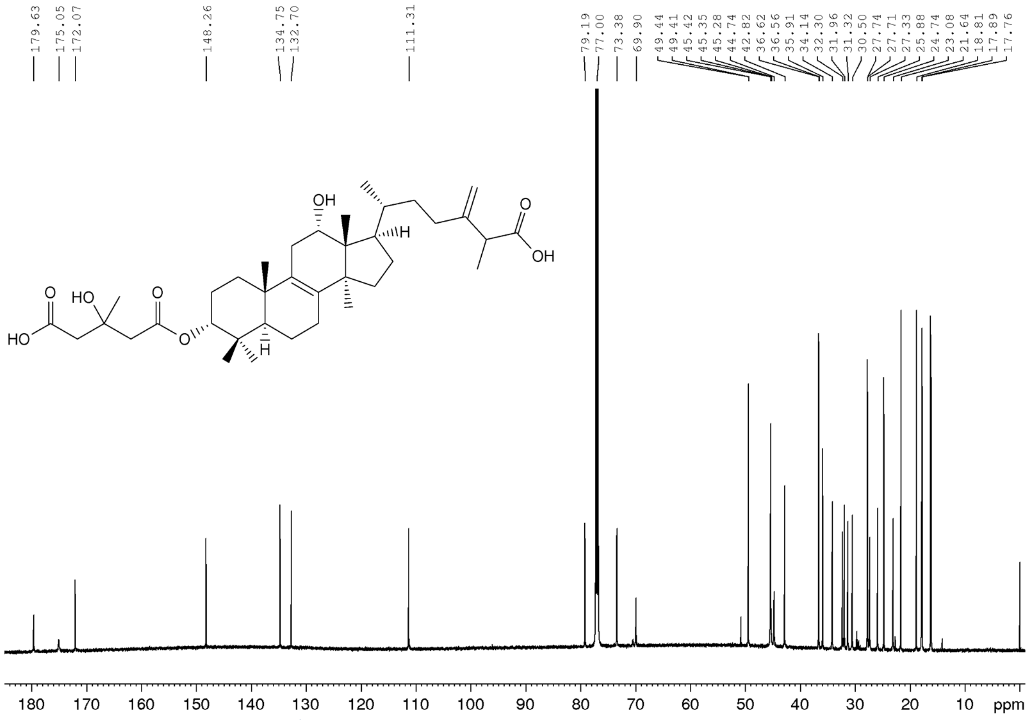

Figure S59. ^{13}C spectrum of compound **7** (150 MHz, $CDCl_3$, 295 K),

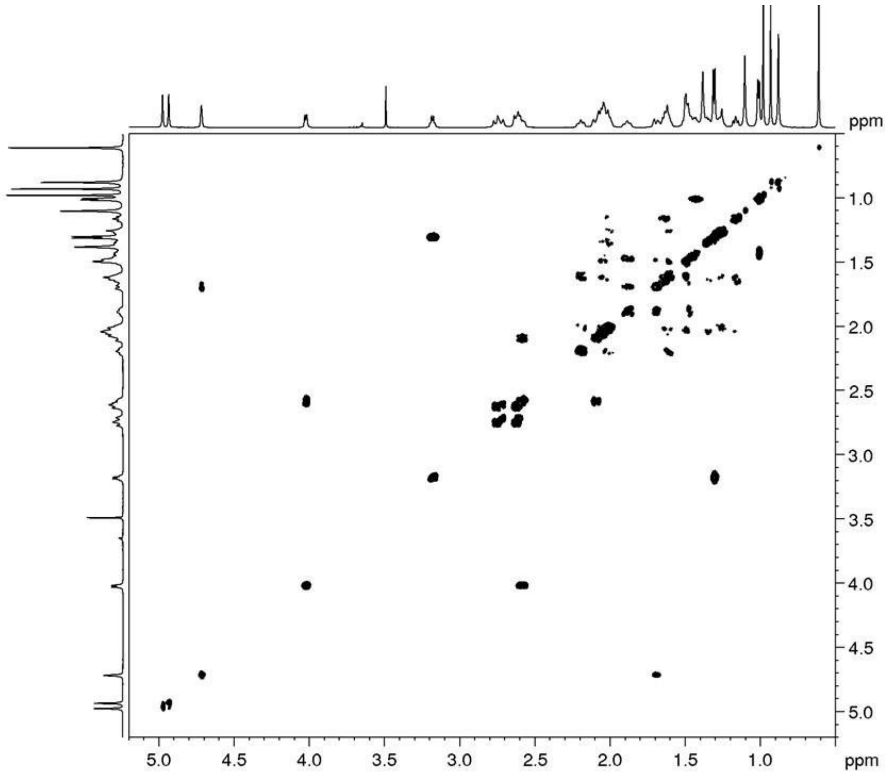

Figure S60. COSY spectrum of compound **7** (600 MHz, $CDCl_3$, 295 K)

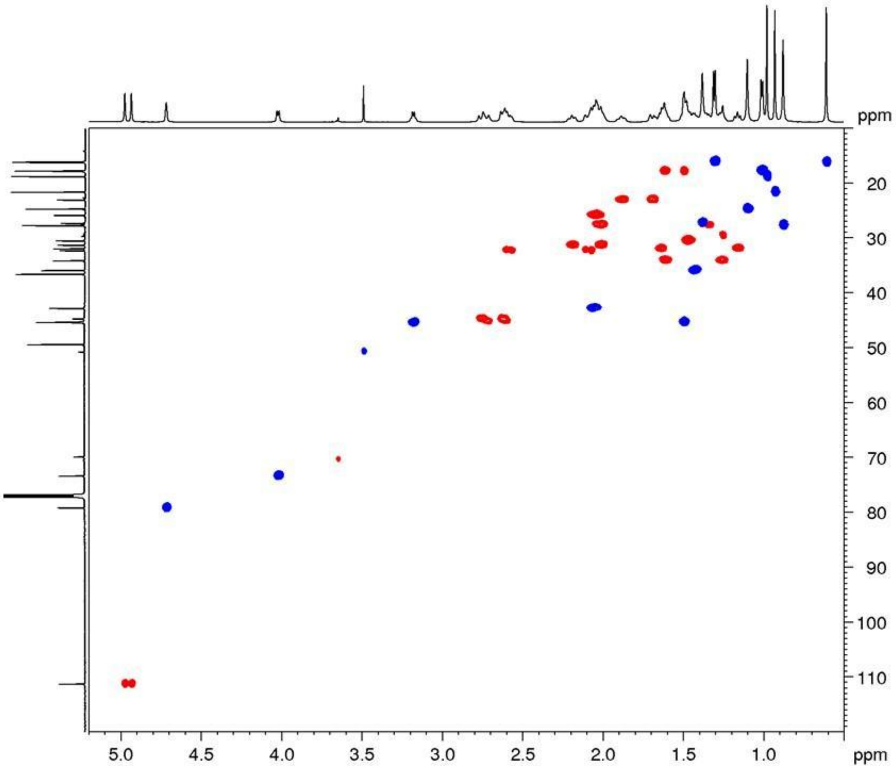

Figure S61. DEPT-edited HSQC spectrum of compound **7** (600 MHz, $CDCl_3$, 295 K)

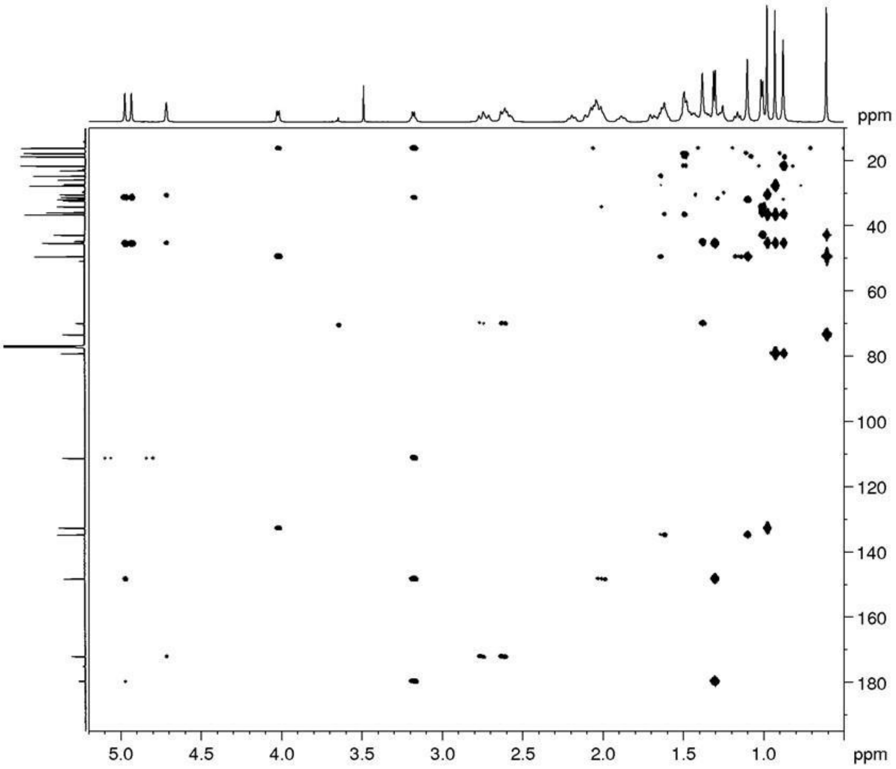

Figure S62. HMBC spectrum of compound **7** (600 MHz, $CDCl_3$, 295 K)

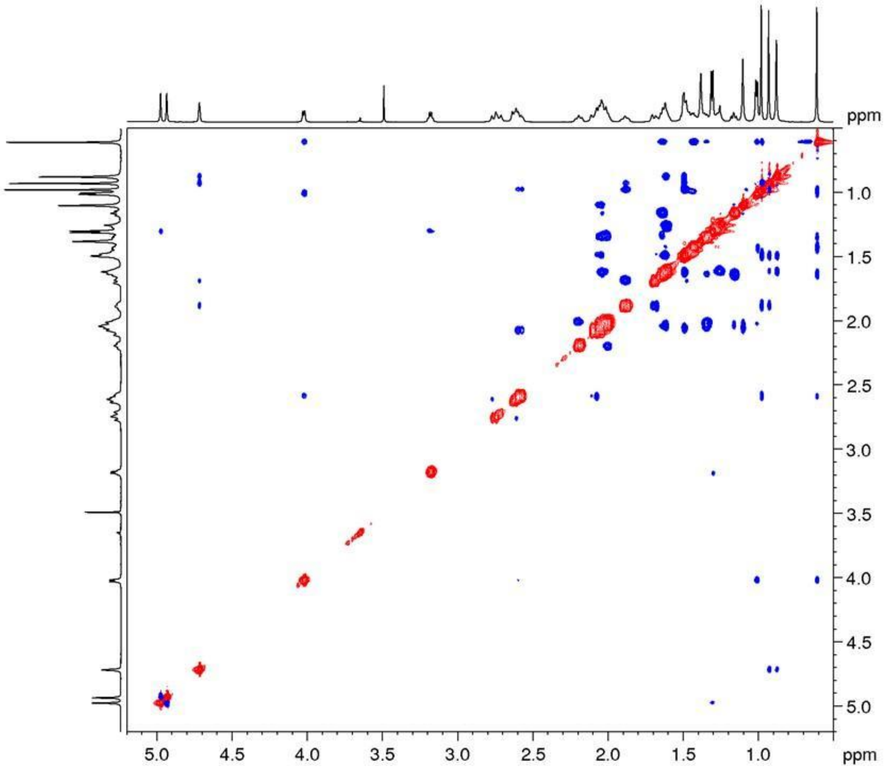

Figure S63. ROESY spectrum of compound **7** (600 MHz, $CDCl_3$, 295 K)

Spectra and spectral data on compound 8

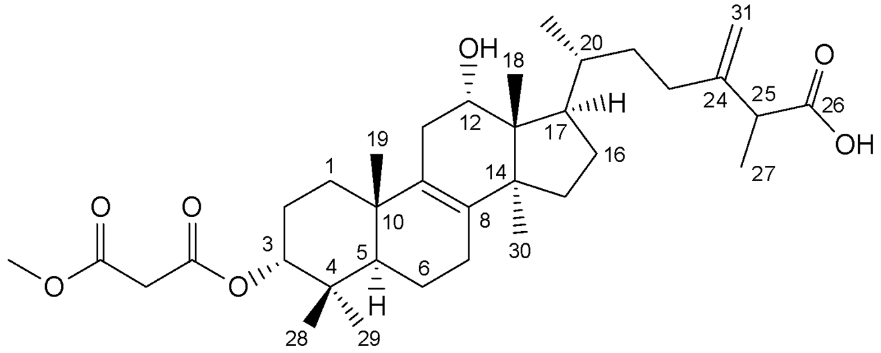

HR-ESI-MS (-) *m/z* 585.3798 [M - H]$^-$ (585.3786 calcd. for $C_{35}H_{53}O_7$; Δ 2.1 ppm); HR-ESI-MSMS (CID = 15%, 30%, 45%) 541.3893, 509.3629, 441.3734

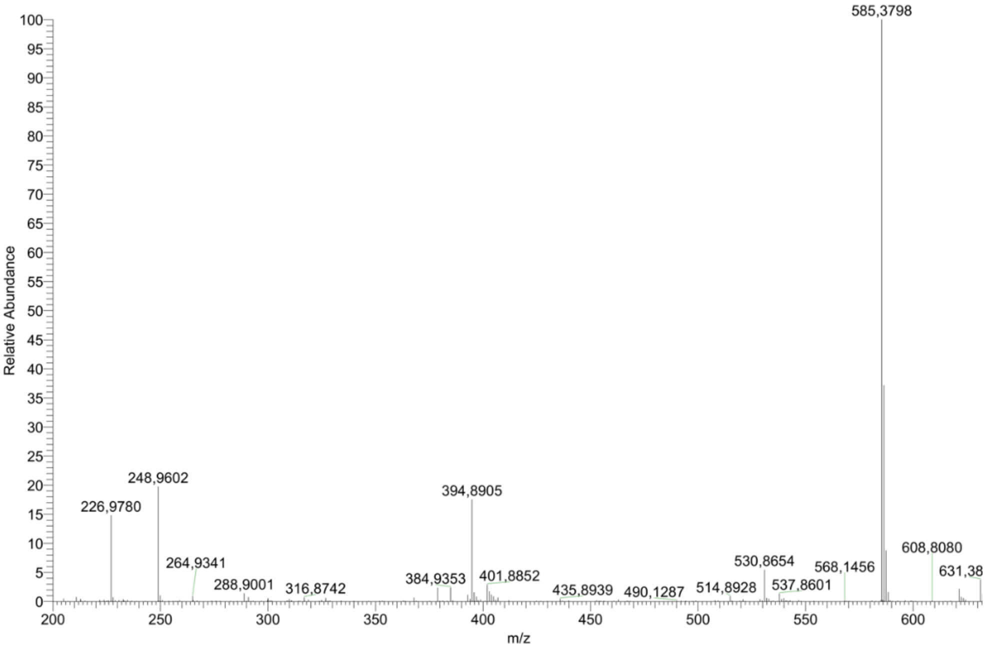

Figure S64. HR-ESI-MS spectrum of compound **8**

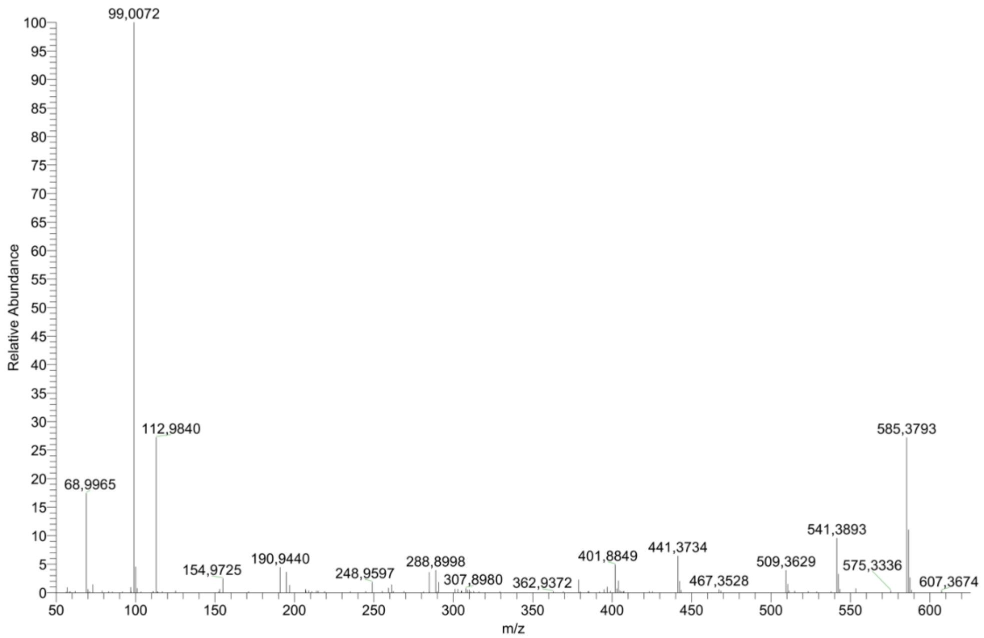

Figure S65. MS-MS spectrum of compound **8**

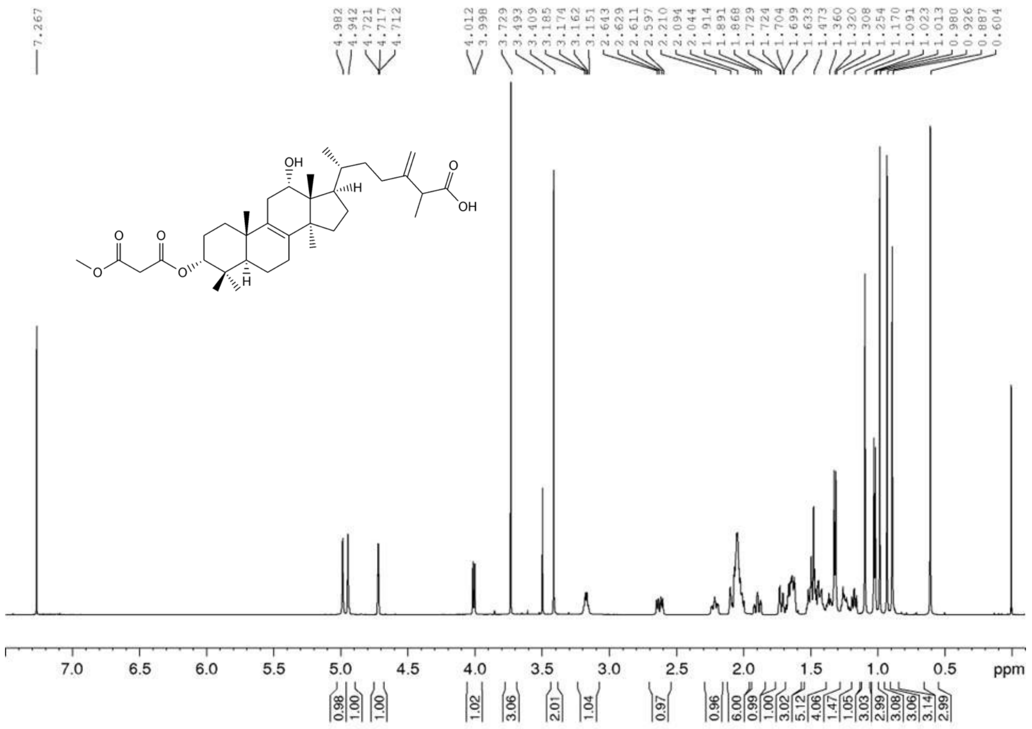

Figure S66. ^{1}H spectrum of compound **8** (600 MHz, $CDCl_3$, 295 K)

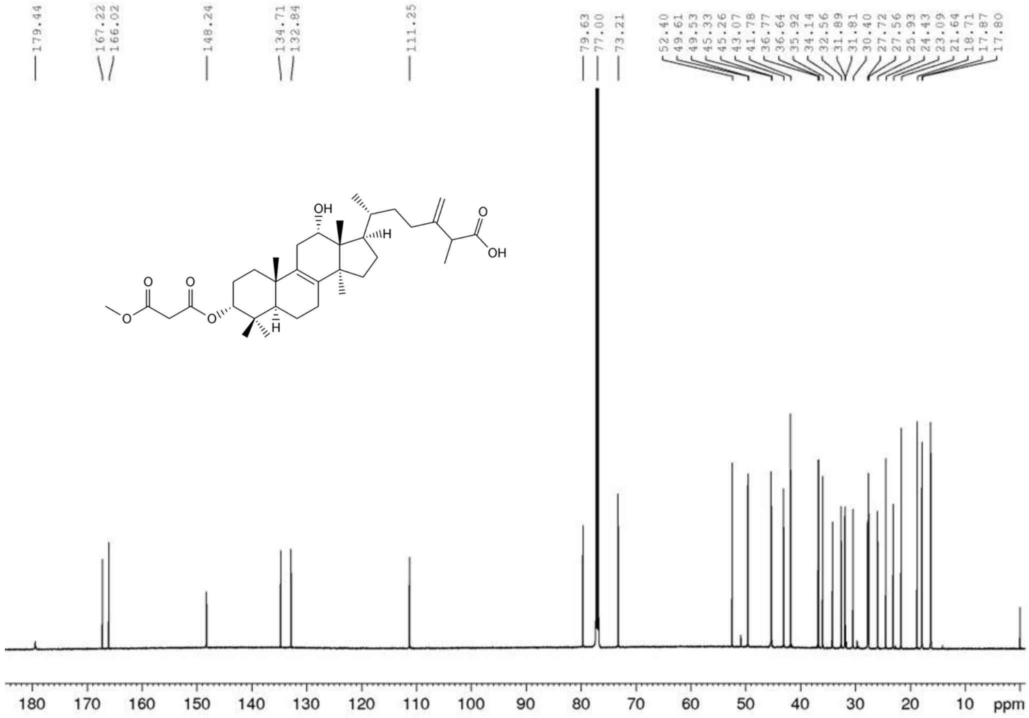

Figure S67. ^{13}C spectrum of compound **8** (150 MHz, $CDCl_3$, 295 K)

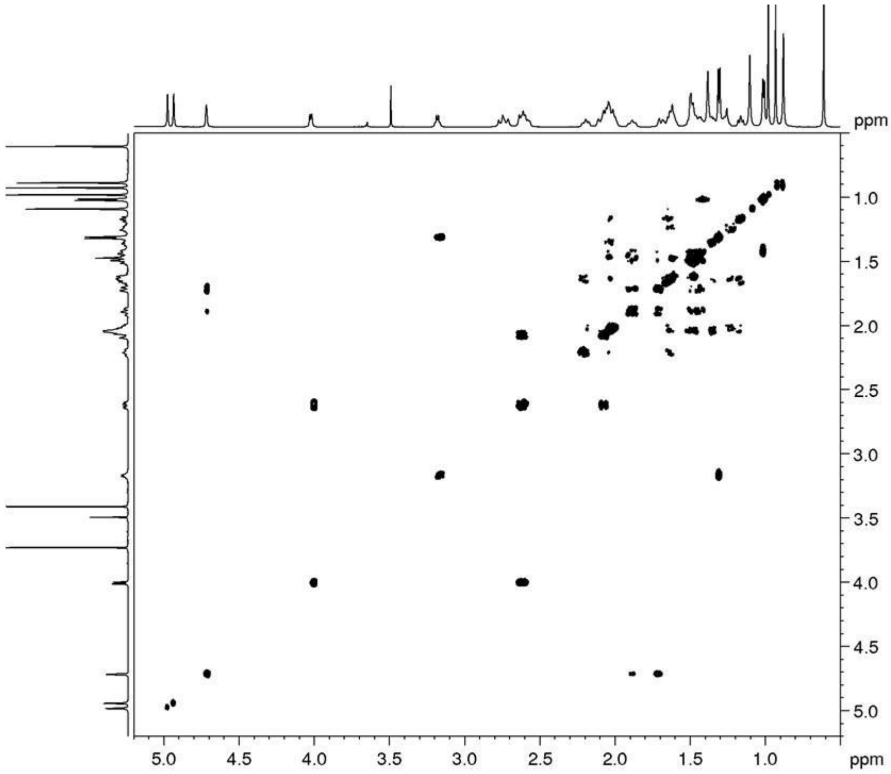

Figure S68. COSY spectrum of compound **8** (600 MHz, $CDCl_3$, 295 K)

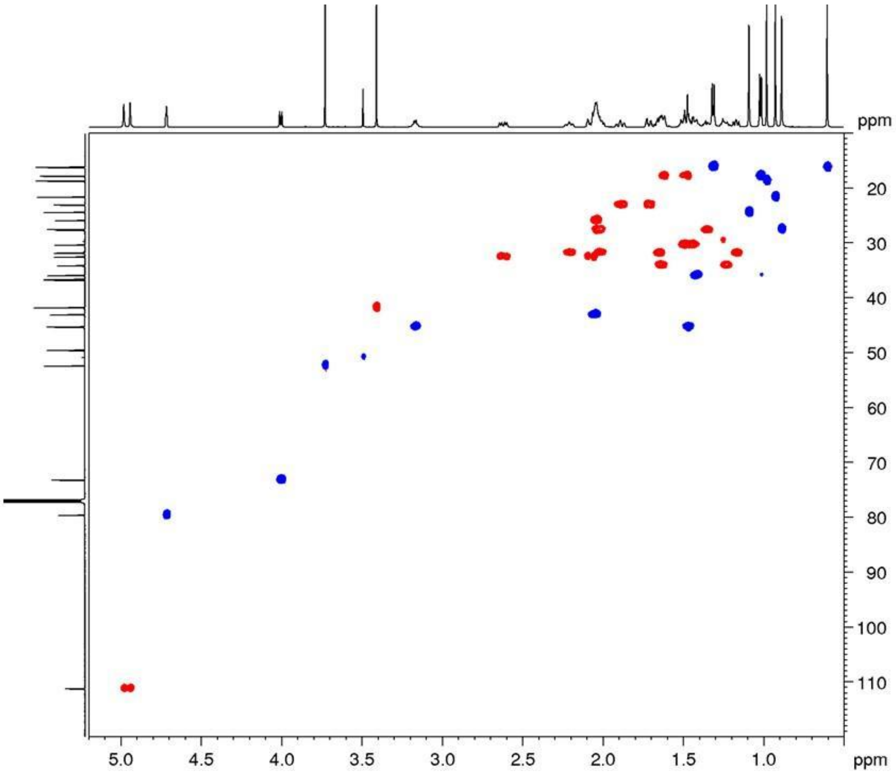

Figure S69. DEPT-edited HSQC spectrum of compound **8** (600 MHz, $CDCl_3$, 295 K)

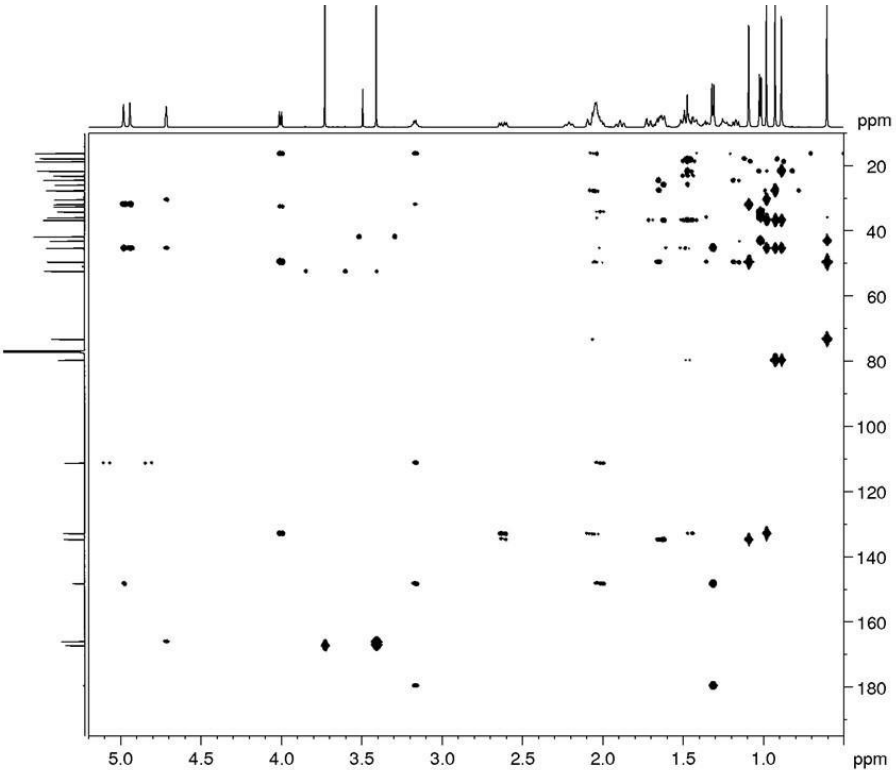

Figure S70. HMBC spectrum of compound **8** (600 MHz, $CDCl_3$, 295 K)

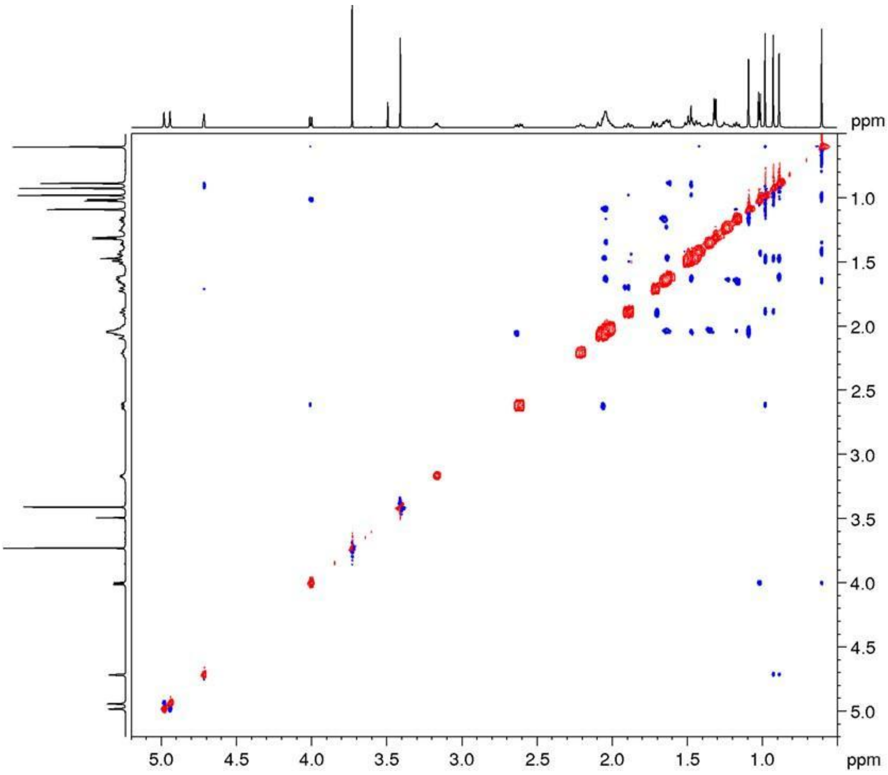

Figure S71. ROESY spectrum of compound **8** (600 MHz, $CDCl_3$, 295 K)

Spectra and spectral data on compound 9

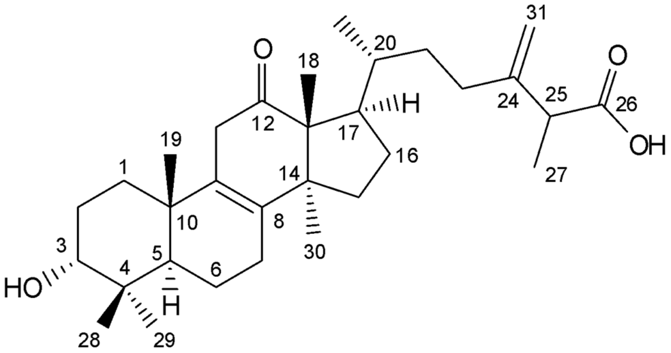

HR-ESI-MS (+) *m/z* 485.3616 [M + H]$^+$ (485.3625 calcd. for $C_{31}H_{49}O_4$; Δ -1.9 ppm); HR-ESI-MSMS (CID = 15%, 30%, 45%) 467.3510, 449.3404

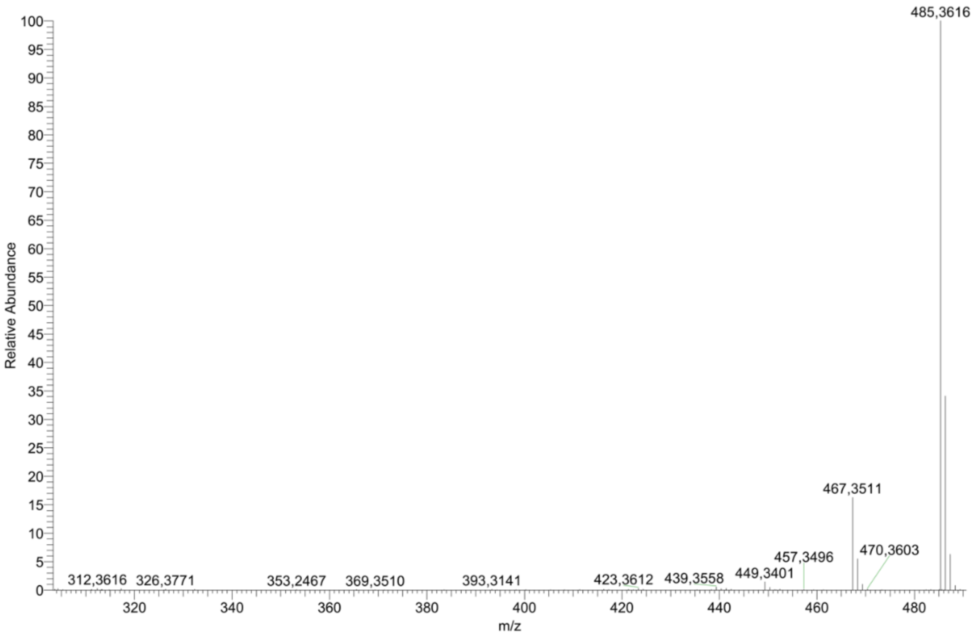

Figure S72. HR-ESI-MS spectrum of compound **9**

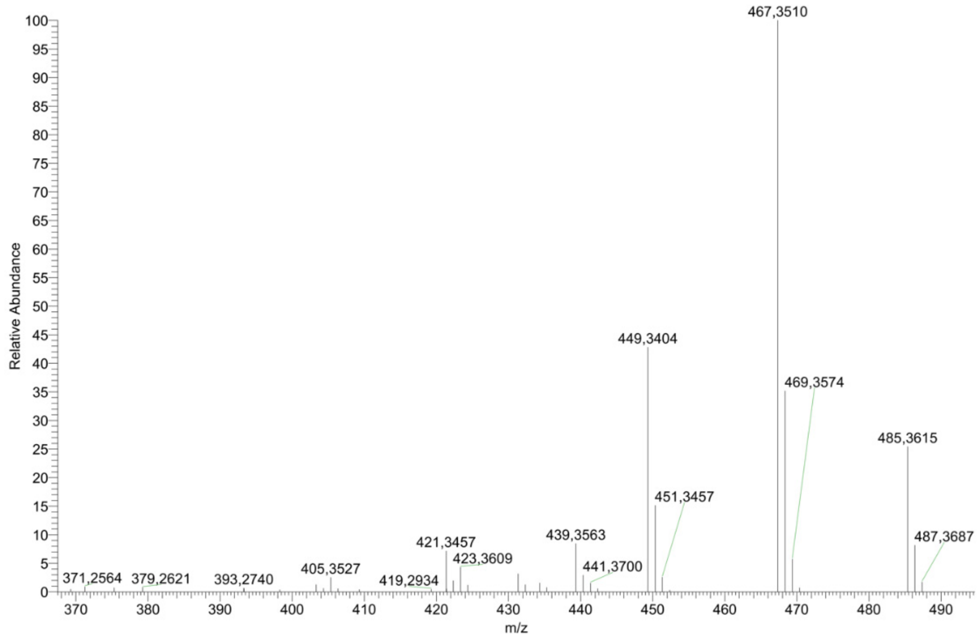

Figure S73. MS-MS spectrum of compound **9**

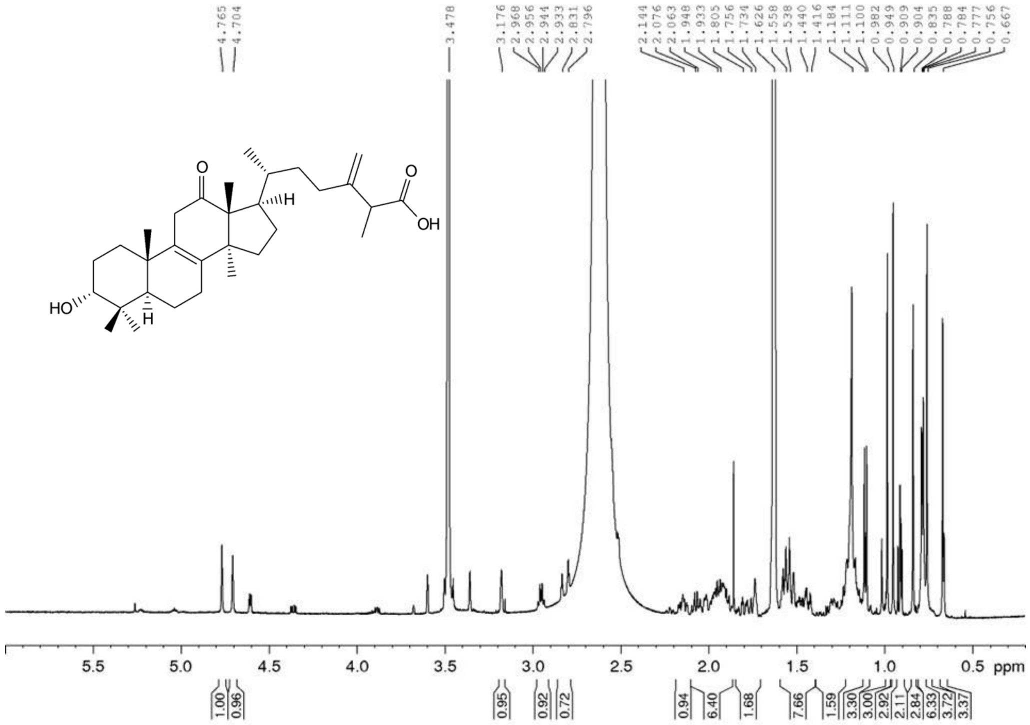

Figure S74. ^{1}H spectrum of compound **9** (600 MHz, $CDCl_3$, 295 K)

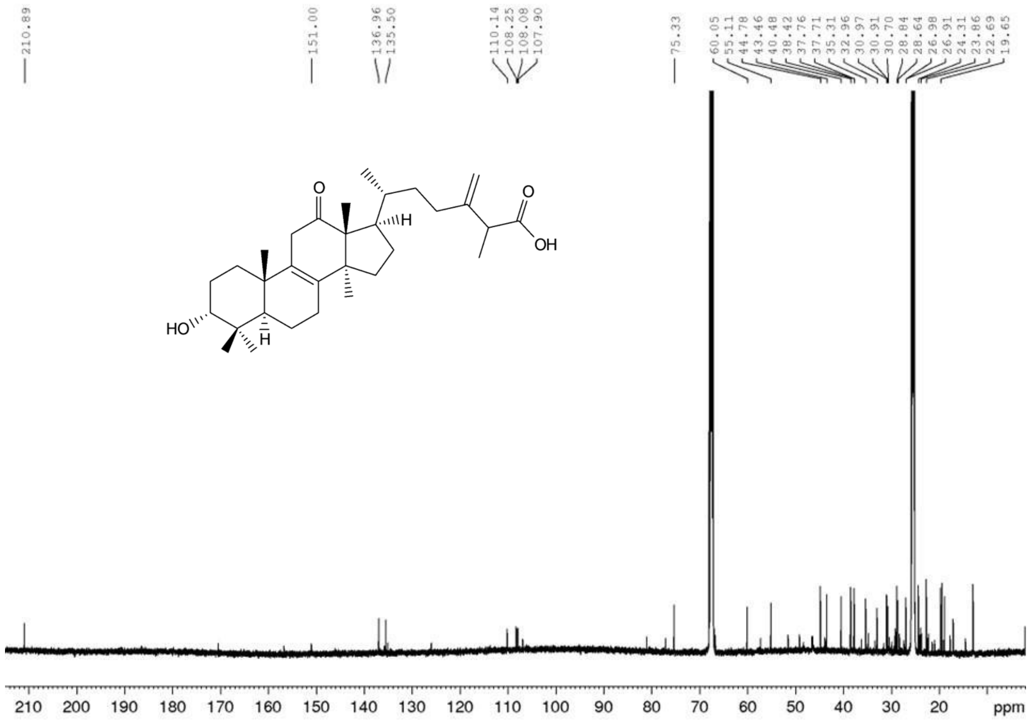

Figure S75. ^{13}C spectrum of compound **9** (150 MHz, $CDCl_3$, 295 K)

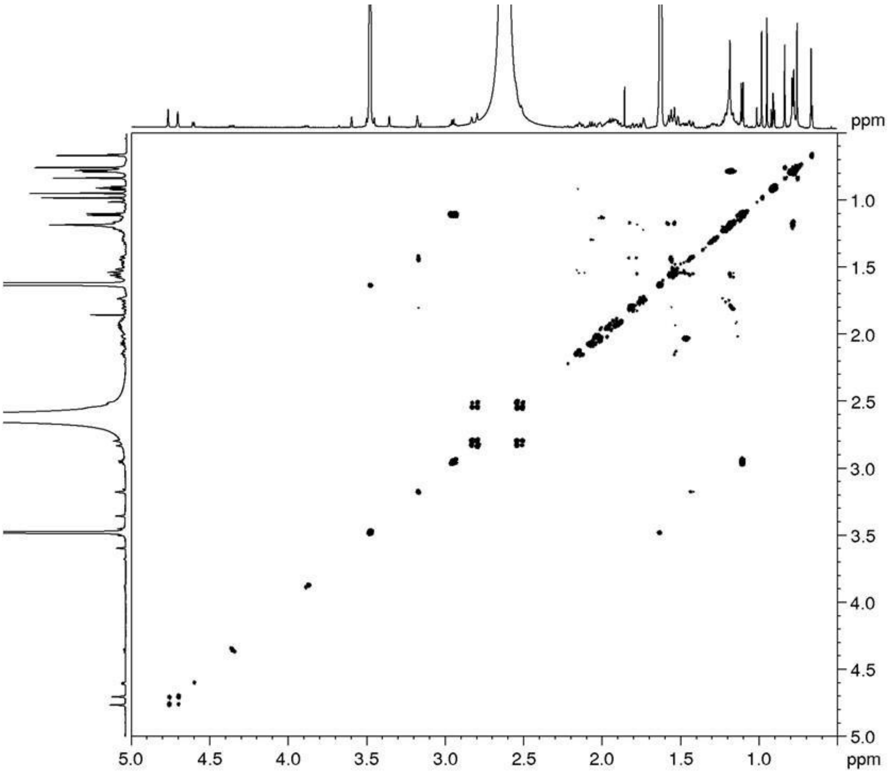

Figure S76. COSY spectrum of compound **9** (600 MHz, $CDCl_3$, 295 K)

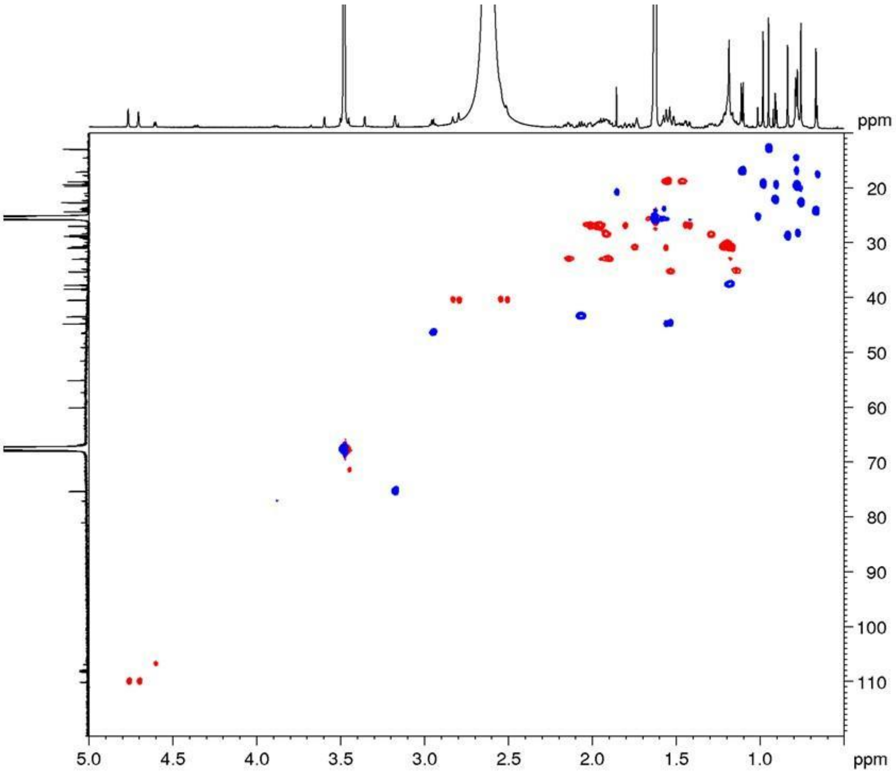

Figure S77. DEPT-edited HSQC spectrum of compound **9** (600 MHz, $CDCl_3$, 295 K)

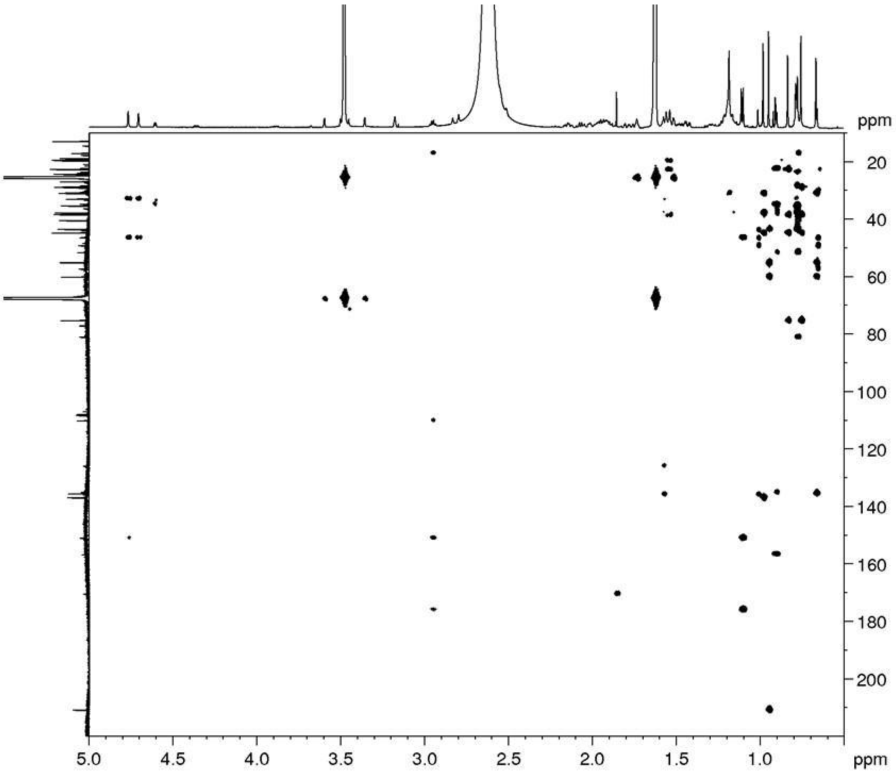

Figure S78. HMBC spectrum of compound **9** (600 MHz, $CDCl_3$, 295 K)

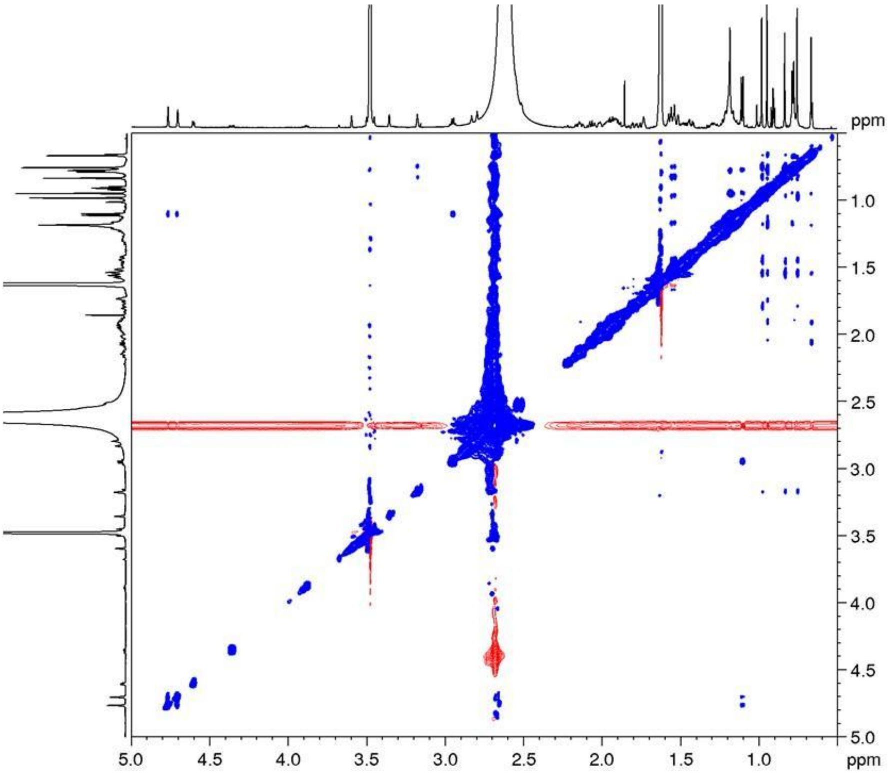

Figure S79. NOESY spectrum of compound **9** (600 MHz, $CDCl_3$, 295 K)

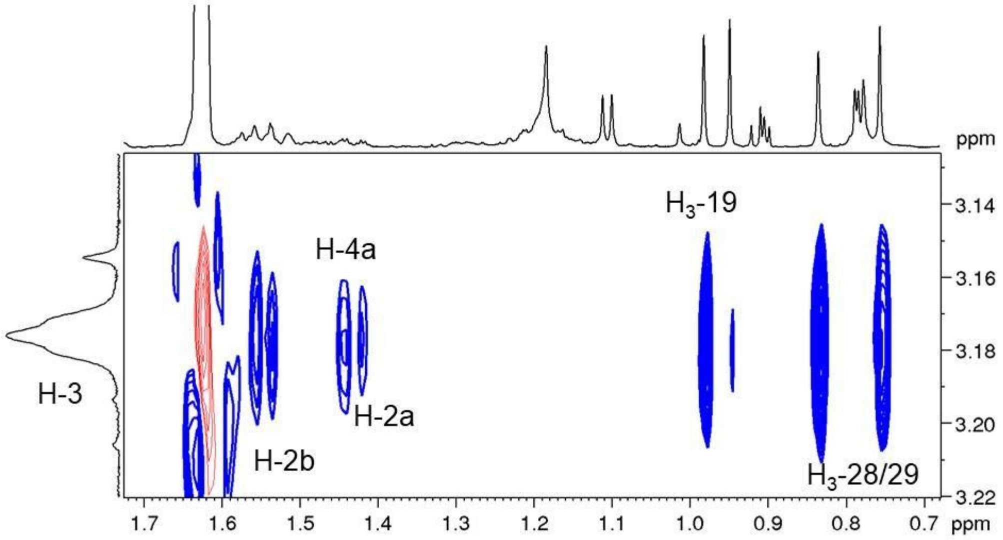

Figure S80. NOESY spectrum of compound **9** (600 MHz, $CDCl_3$, 295 K)

Spectra and spectral data on compound 10

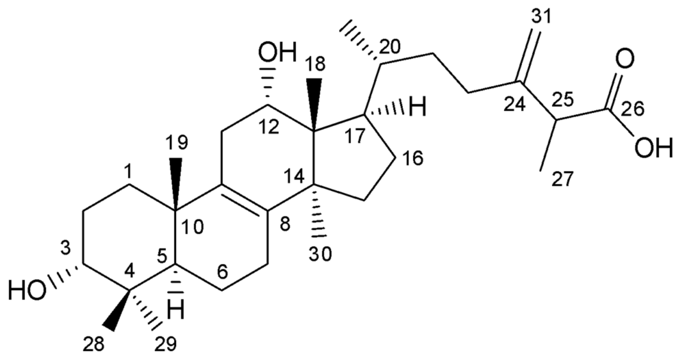

HR-ESI-MS (-) *m/z* 485.3638 $[M - H]^-$ (485.3625 calcd. for $C_{31}H_{49}O_4$; Δ 2.6 ppm); HR-ESI-MSMS (CID = 15%, 30%, 45%) 455.3529, 441.3736, 423.3636

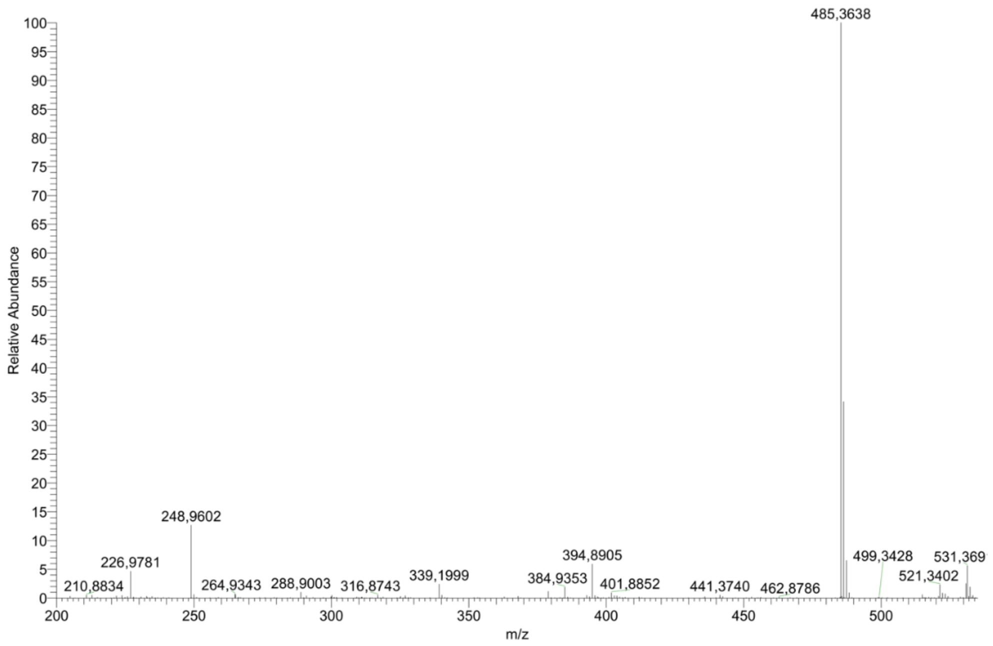

Figure S81. HR-ESI-MS spectrum of compound **10**

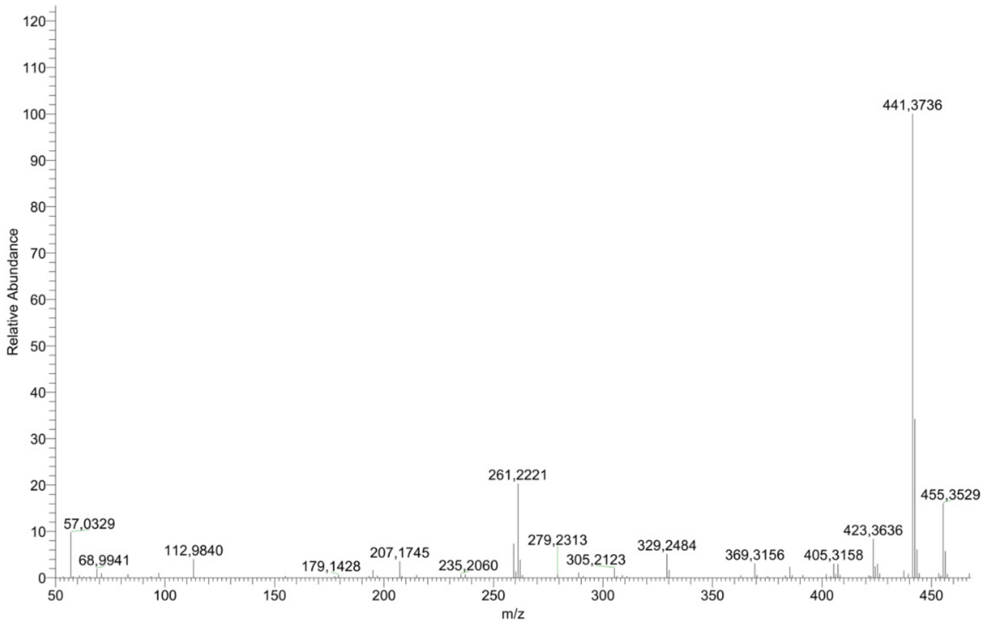

Figure S82. MS-MS spectrum of compound **10**

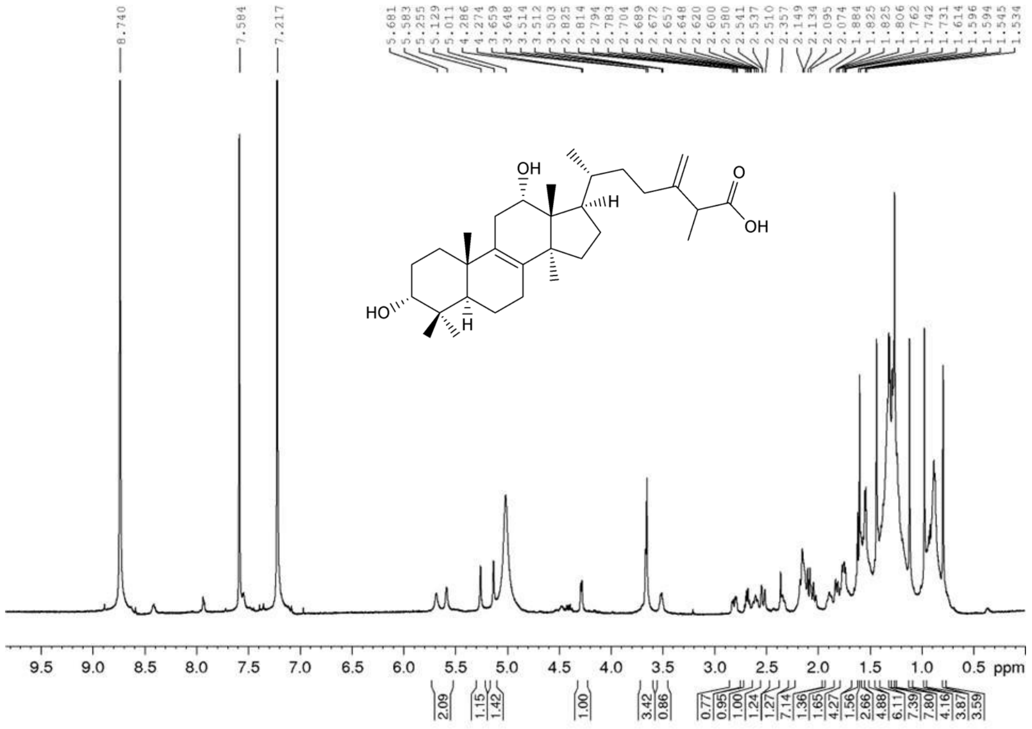

Figure S83. ^{1}H spectrum of compound **10** (600 MHz, pyridine-d_5, 295 K)

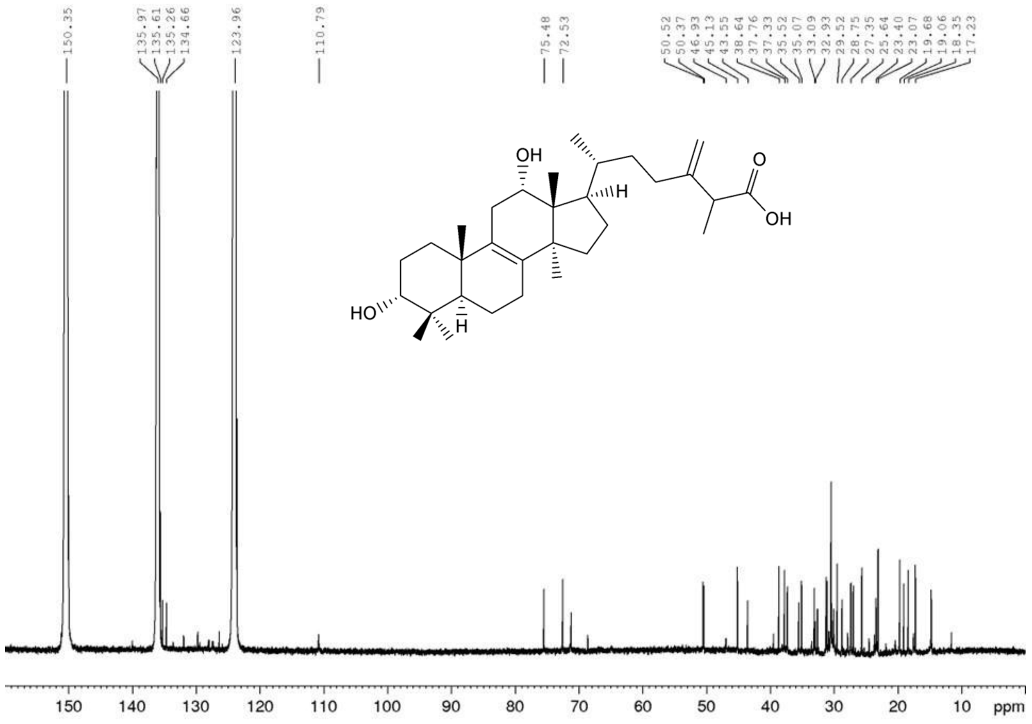

Figure S84. ^{13}C spectrum of compound **10** (150 MHz, pyridine-d_5, 295 K)

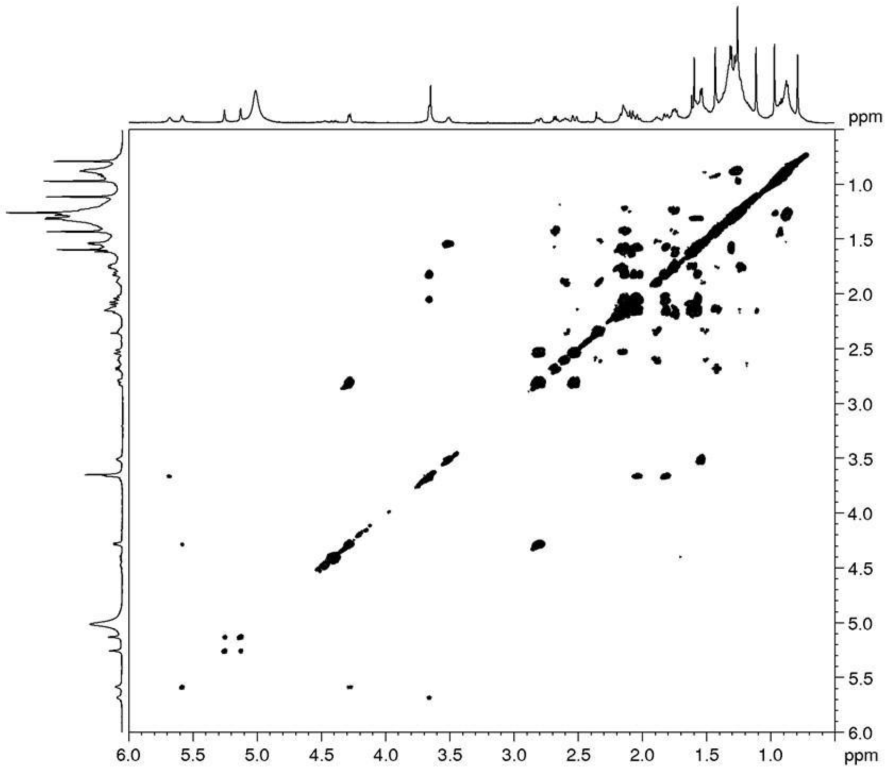

Figure S85. COSY spectrum of compound **10** (600 MHz, pyridine-d_5, 295 K)

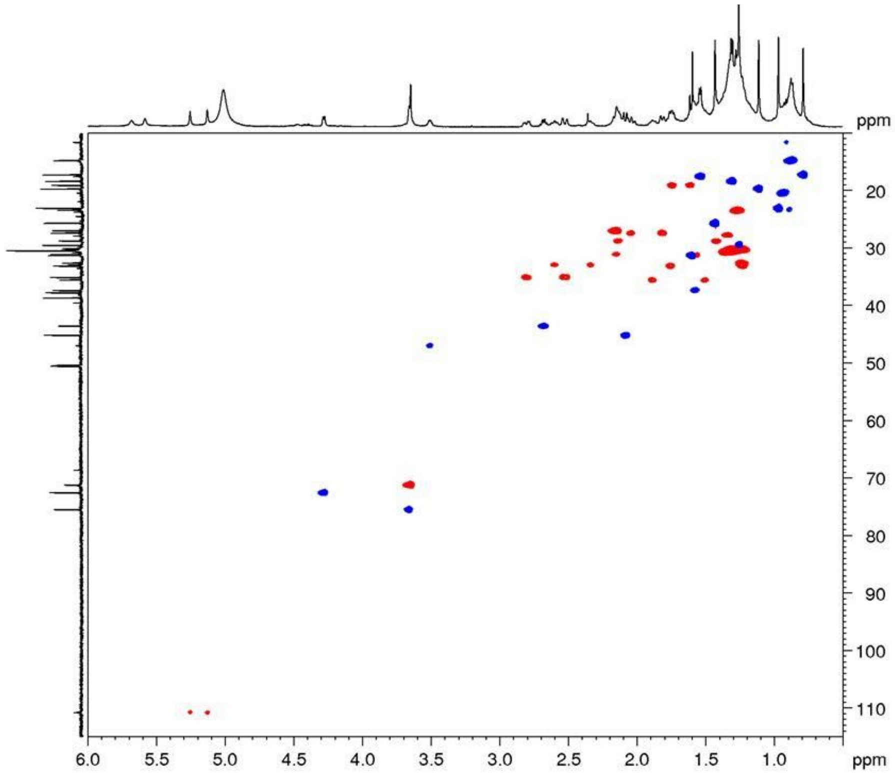

Figure S86. DEPT-edited HSQC spectrum of compound **10** (600 MHz, pyridine-d_5, 295 K)

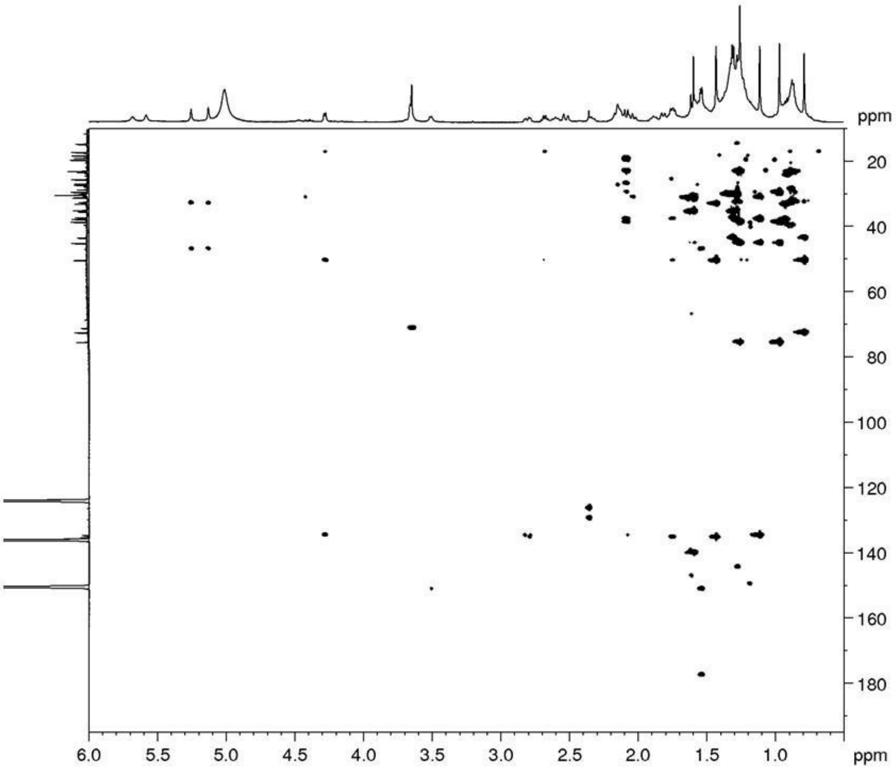

Figure S87. HMBC spectrum of compound **10** (600 MHz, pyridine-d_5, 295 K)

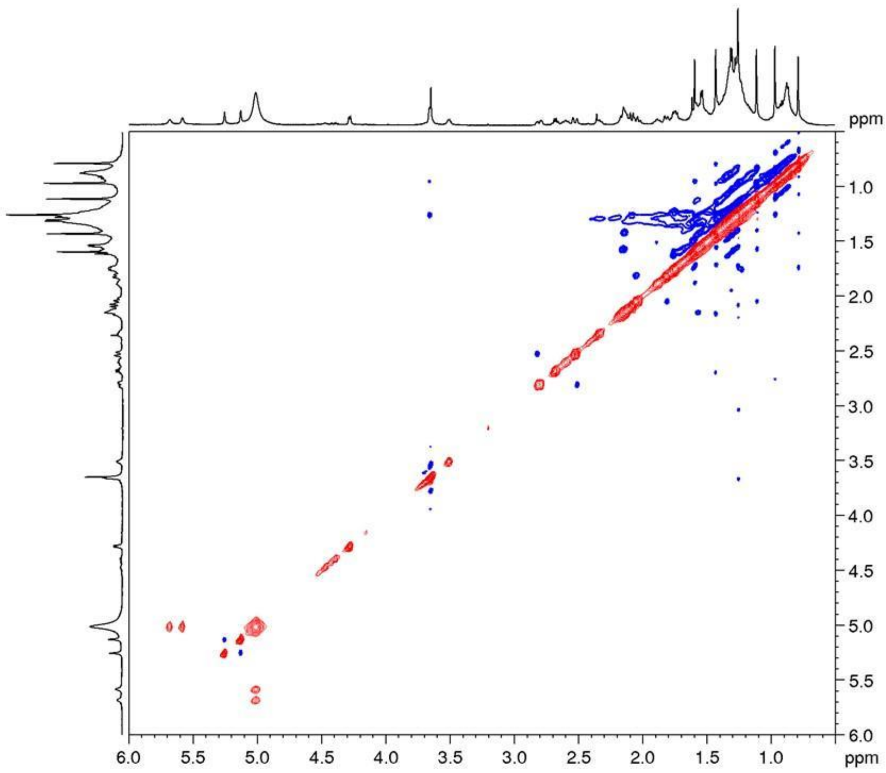

Figure S88. NOESY spectrum of compound **10** (600 MHz, pyridine-d_5, 295 K)

Spectra and spectral data on compound 11

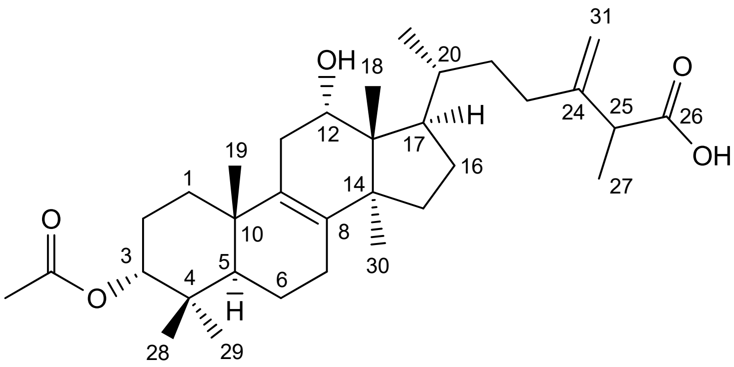

HR-ESI-MS (-) *m/z* 527.3743 [M - H]$^-$ (527.3731 calcd. for $C_{34}H_{51}O_5$ *Δ* 2.3 ppm); HR-ESI-MSMS (CID = 15%, 30%, 45%) 497.3636, 483.3841, 441.3736

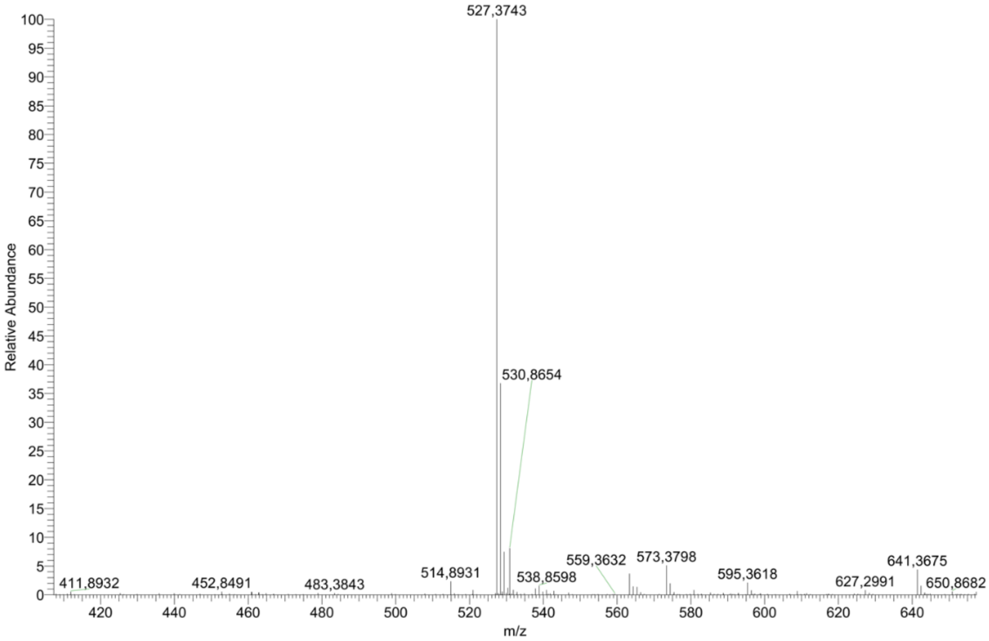

Figure S89. HR-ESI-MS spectrum of compound **11**

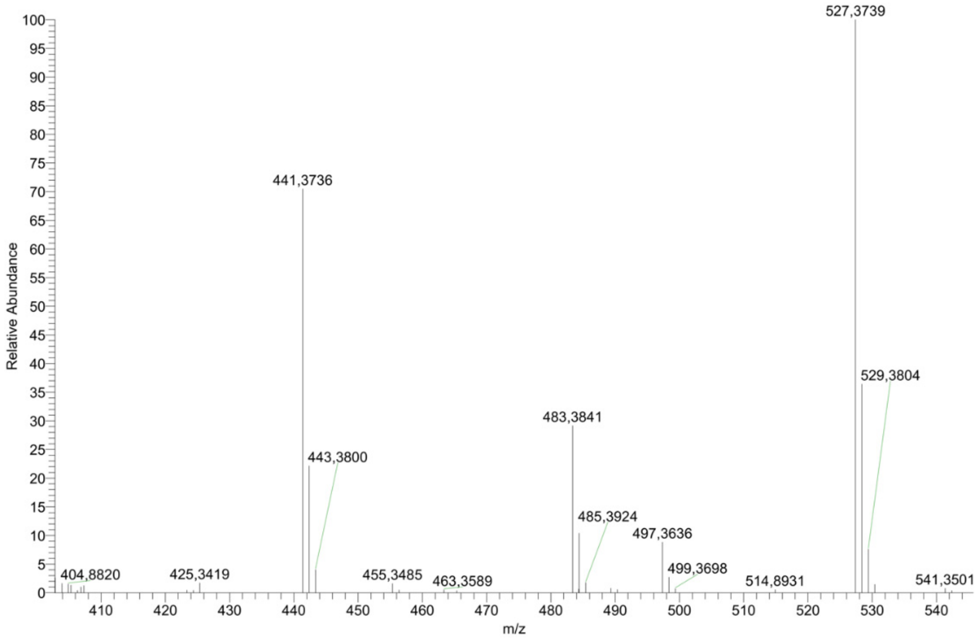

Figure S90. MS-MS spectrum of compound **11**

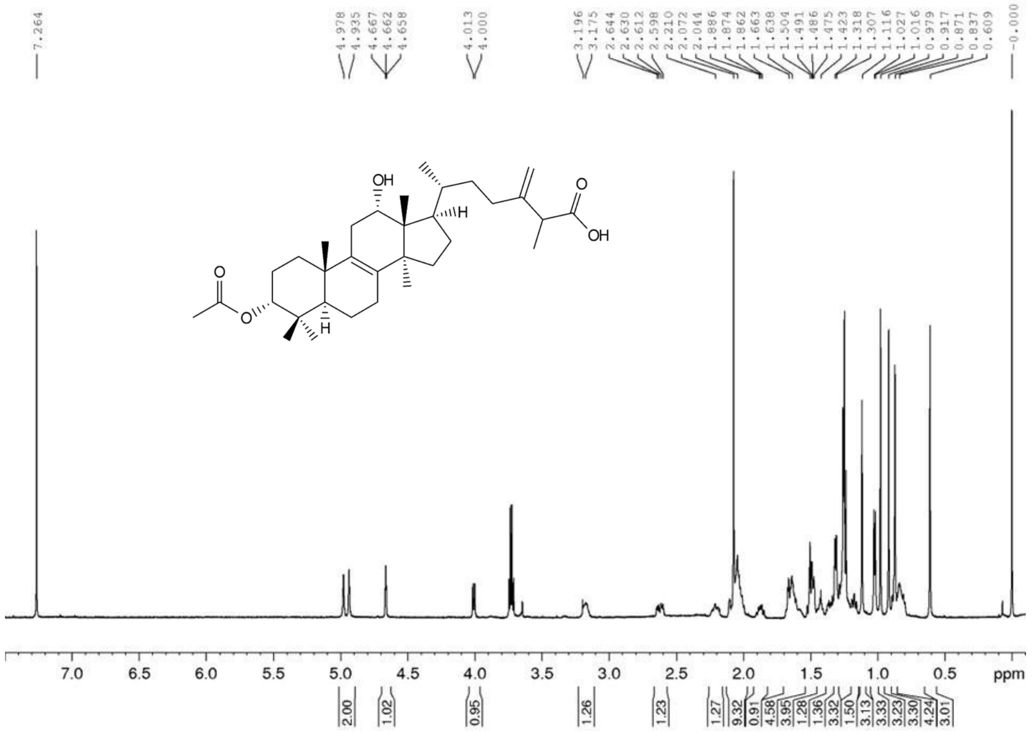

Figure S91. ^{1}H spectrum of compound **11** (600 MHz, $CDCl_3$, 295 K)

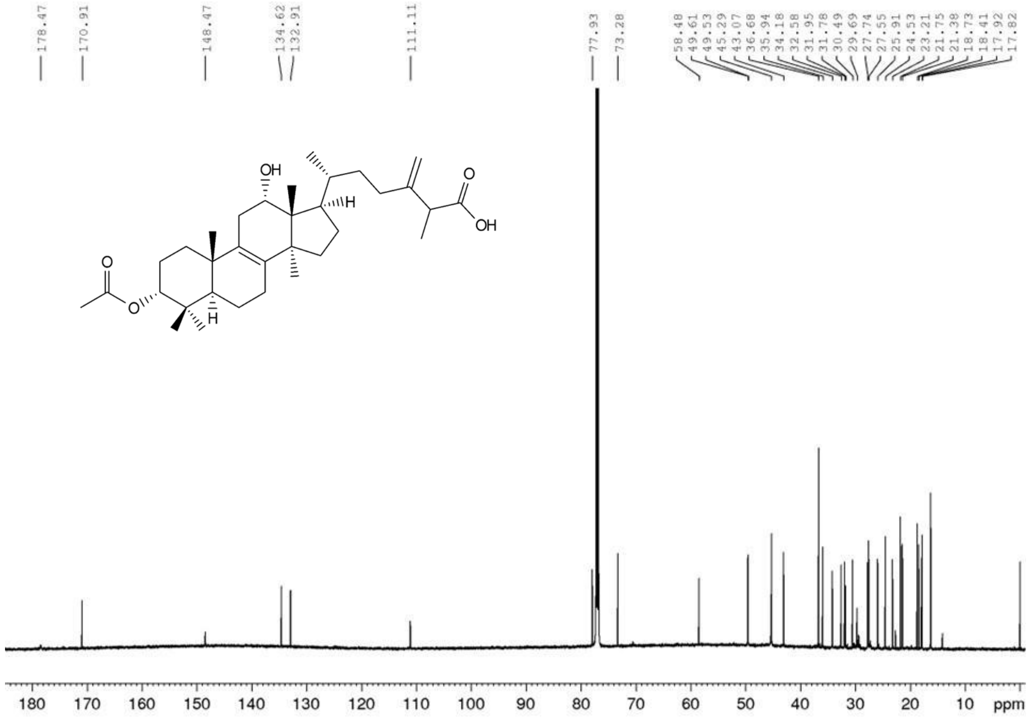

Figure S92. ^{13}C spectrum of compound **11** (150 MHz, $CDCl_3$, 295 K)

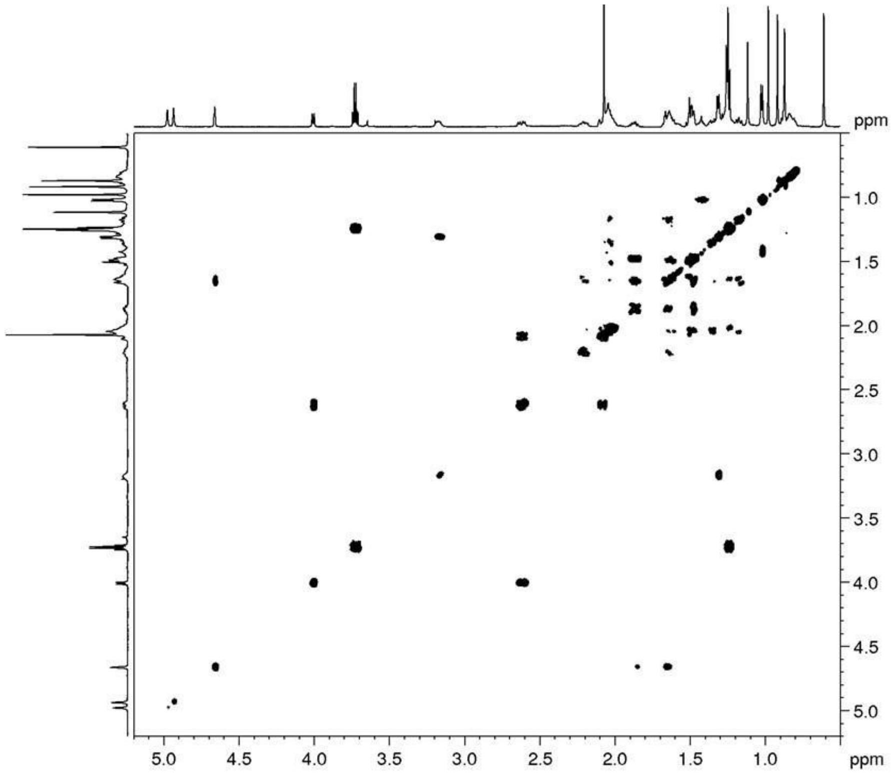

Figure S93. COSY spectrum of compound **11** (600 MHz, $CDCl_3$, 295 K)

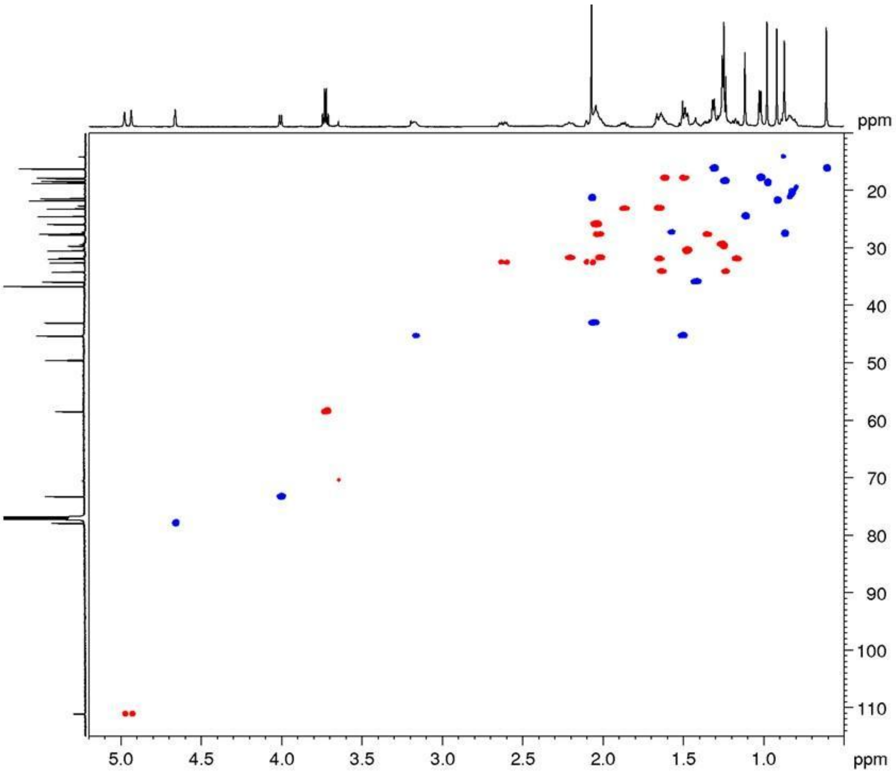

Figure S94. DEPT-edited HSQC spectrum of compound **11** (600 MHz, $CDCl_3$, 295 K)

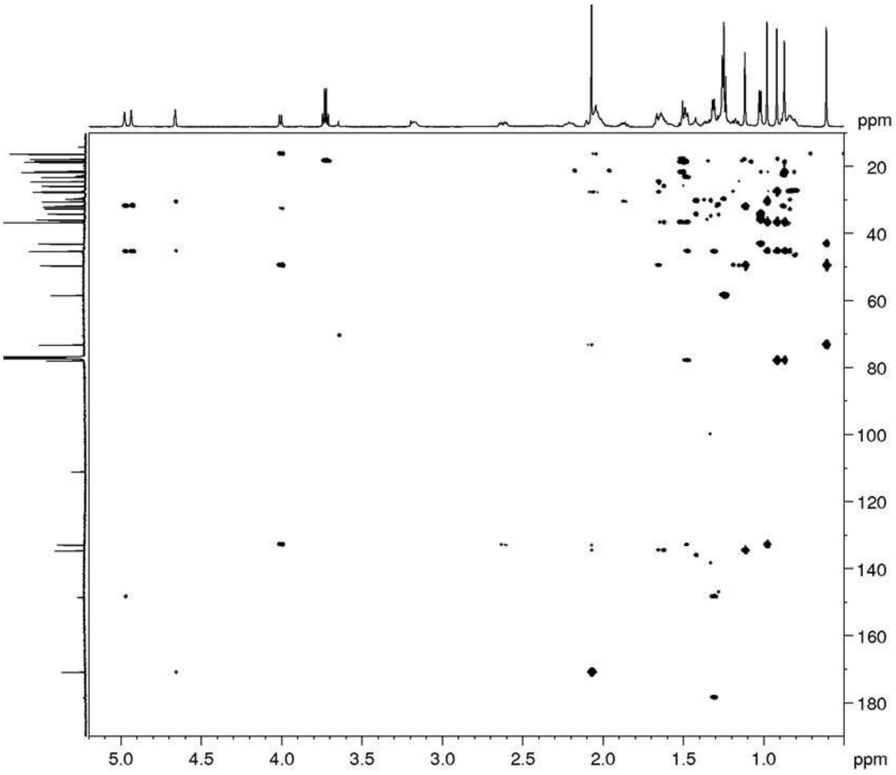

Figure S95. HMBC spectrum of compound **11** (600 MHz, $CDCl_3$, 295 K)

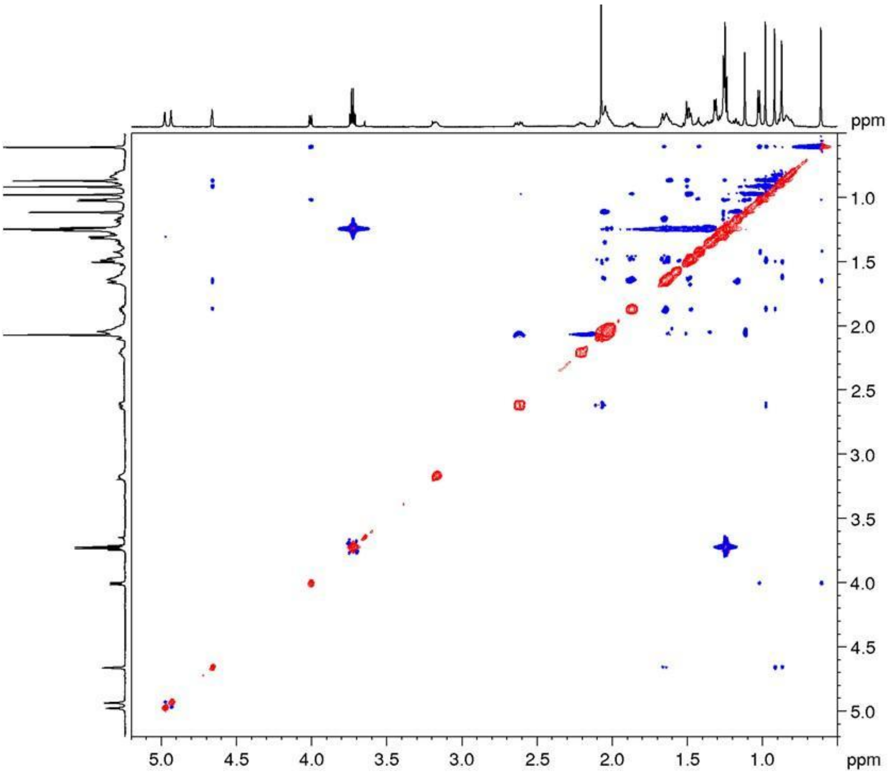

Figure S96. NOESY spectrum of compound **11** (600 MHz, $CDCl_3$, 295 K)

Spectra and spectral data on compound 12

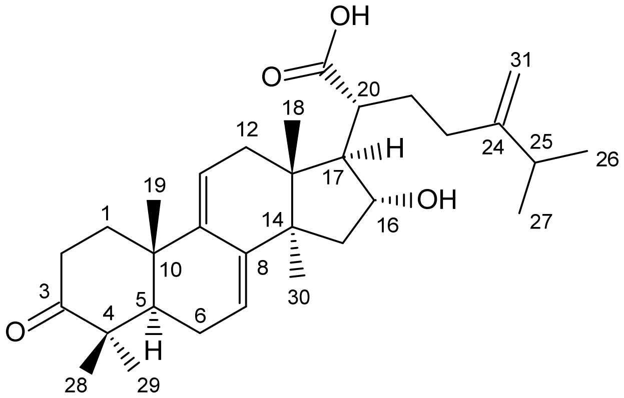

HR-ESI-MS (+) *m/z* 483.3461 [M + H]$^+$ (483.3469 calcd. for $C_{31}H_{47}O_4$; Δ -1.6 ppm); HR-ESI-MSMS (CID = 15%, 30%, 45%) 465.3354, 309.2205

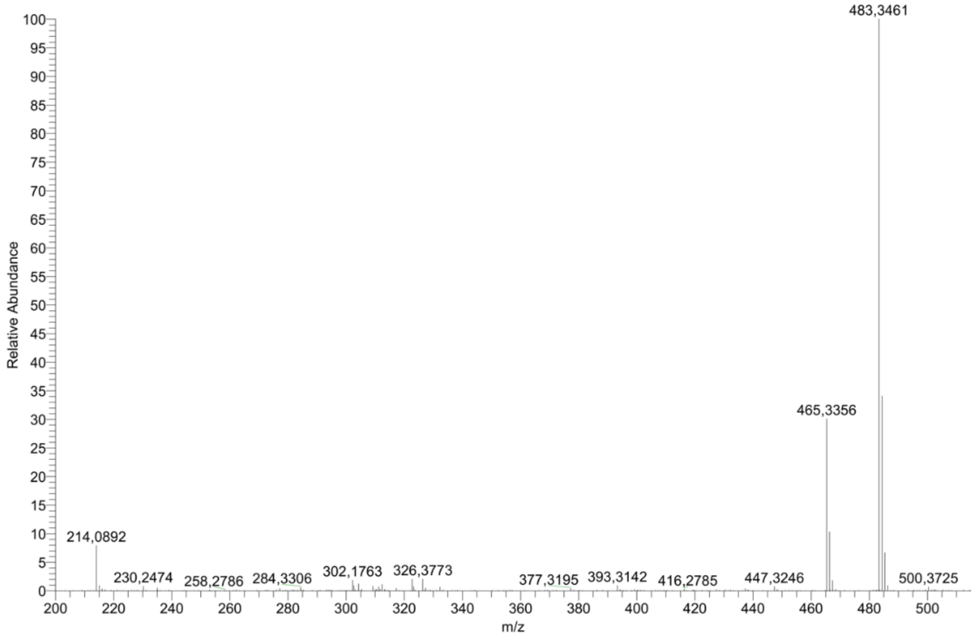

Figure S97. HR-ESI-MS spectrum of compound **12**

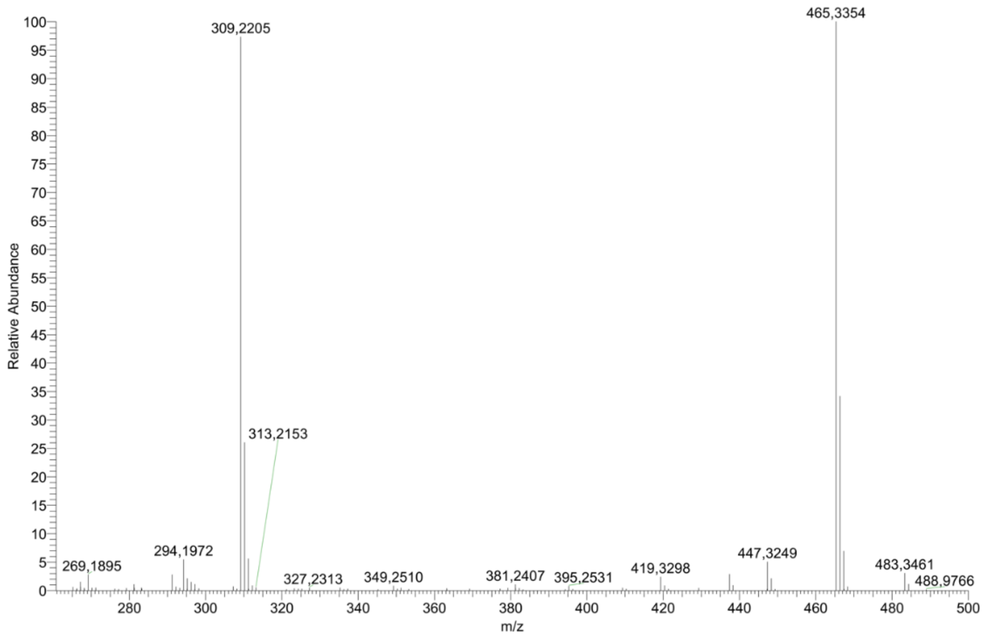

Figure S98. MS-MS spectrum of compound **12**

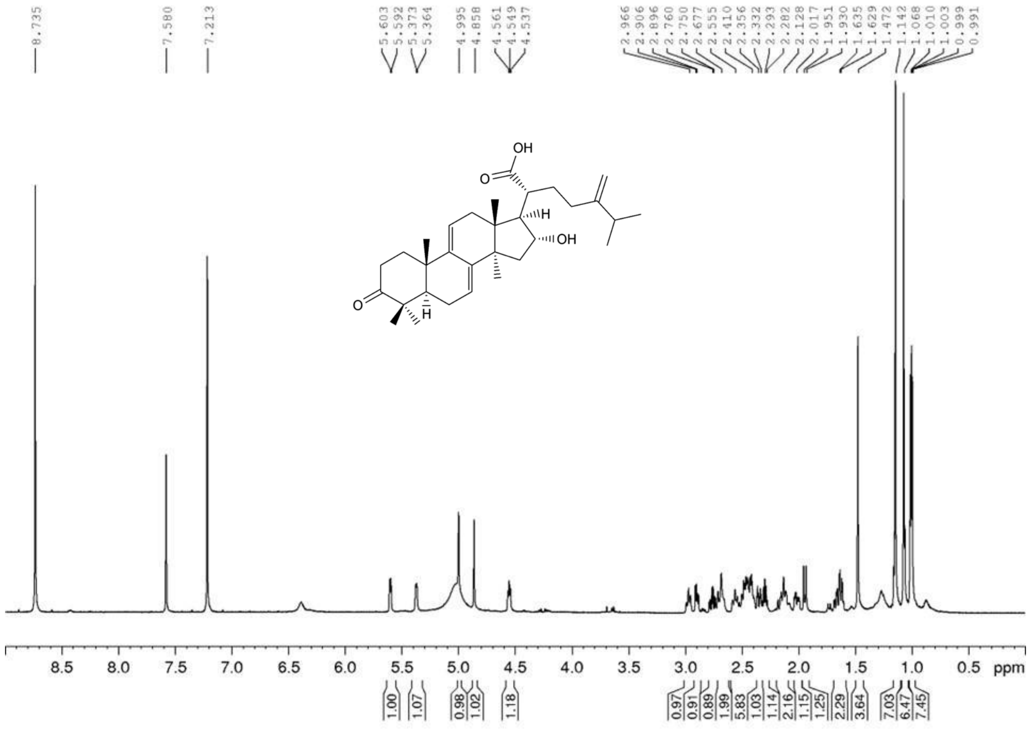

Figure S99. ^{1}H spectrum of compound **12** (600 MHz, pyridine-d_5, 295 K)

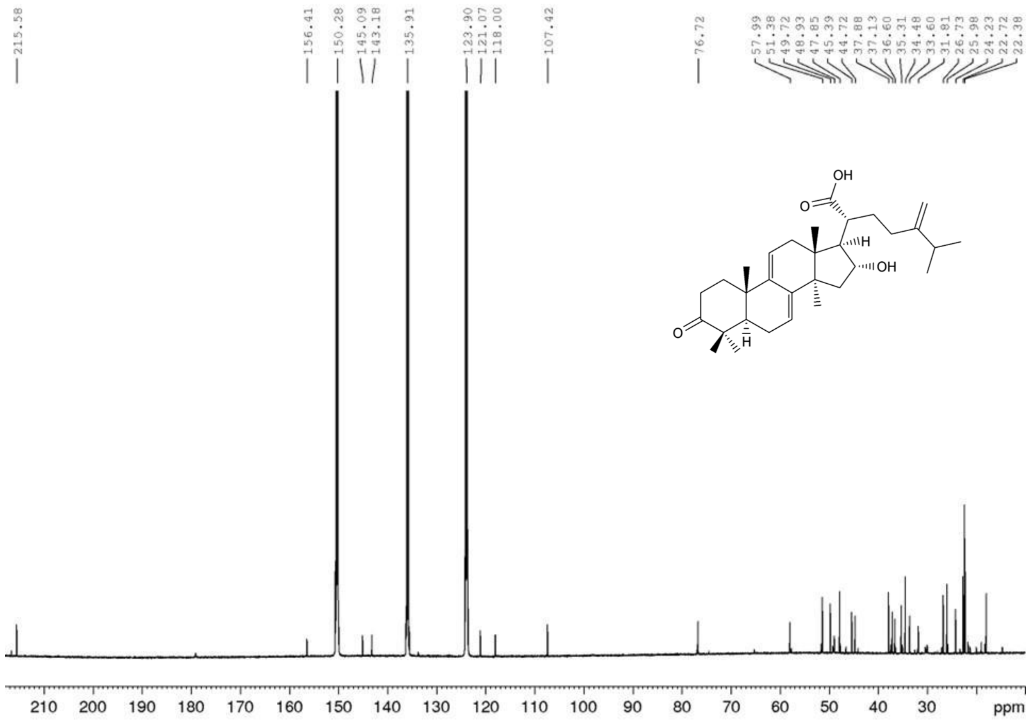

Figure S100. ^{13}C spectrum of compound **12** (150 MHz, pyridine-d_5, 295 K)

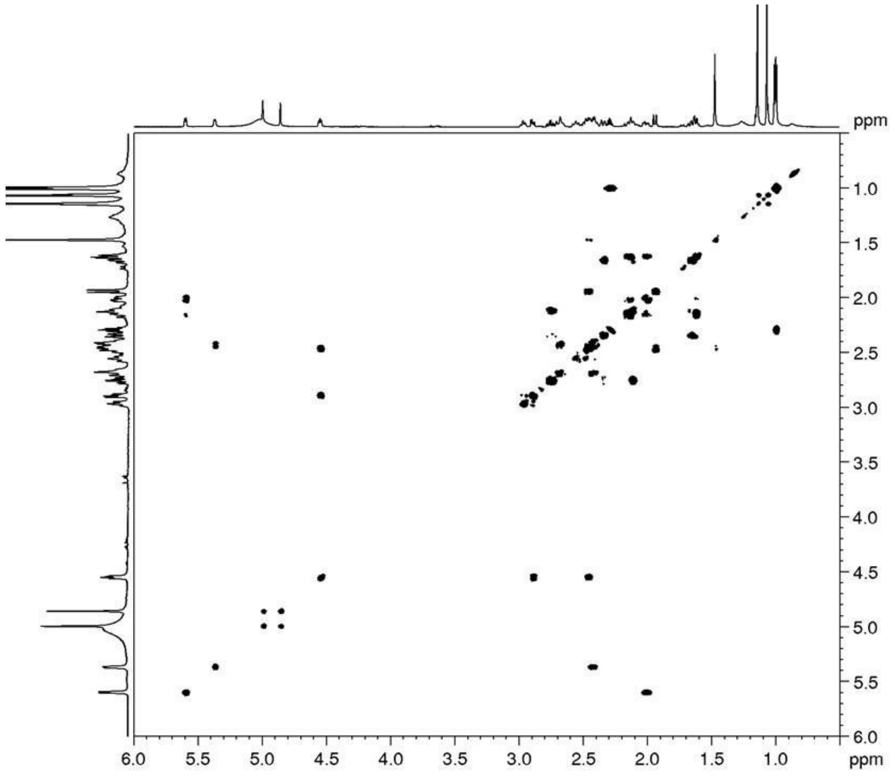

Figure S101. COSY spectrum of compound **12** (600 MHz, pyridine-d_5, 295 K)

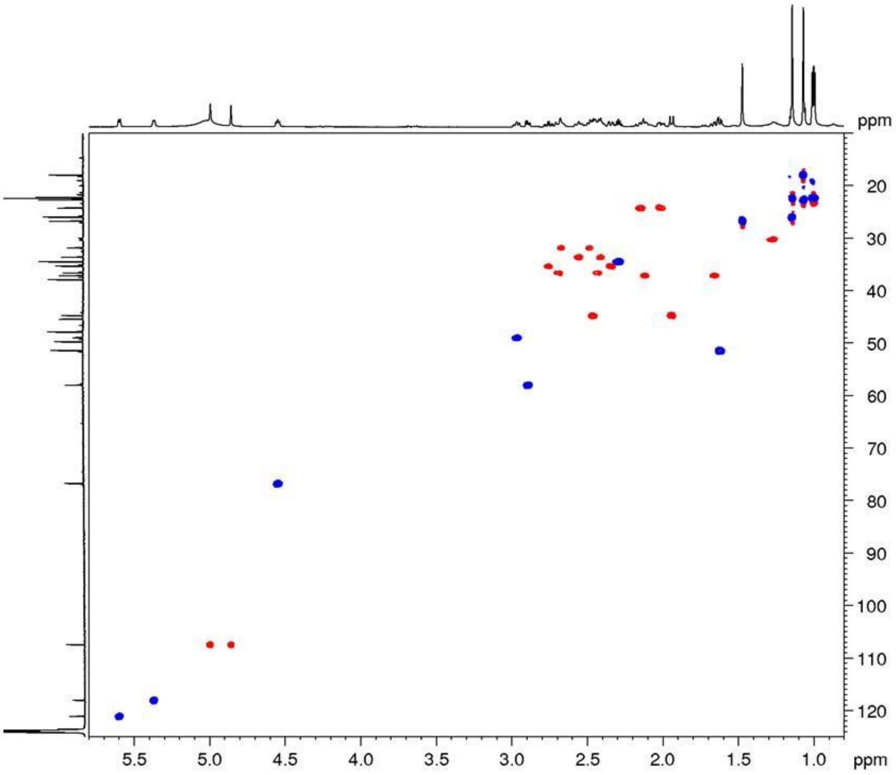

Figure S102. DEPT-edited HSQC spectrum of compound **12** (600 MHz, pyridine-d_5, 295 K)

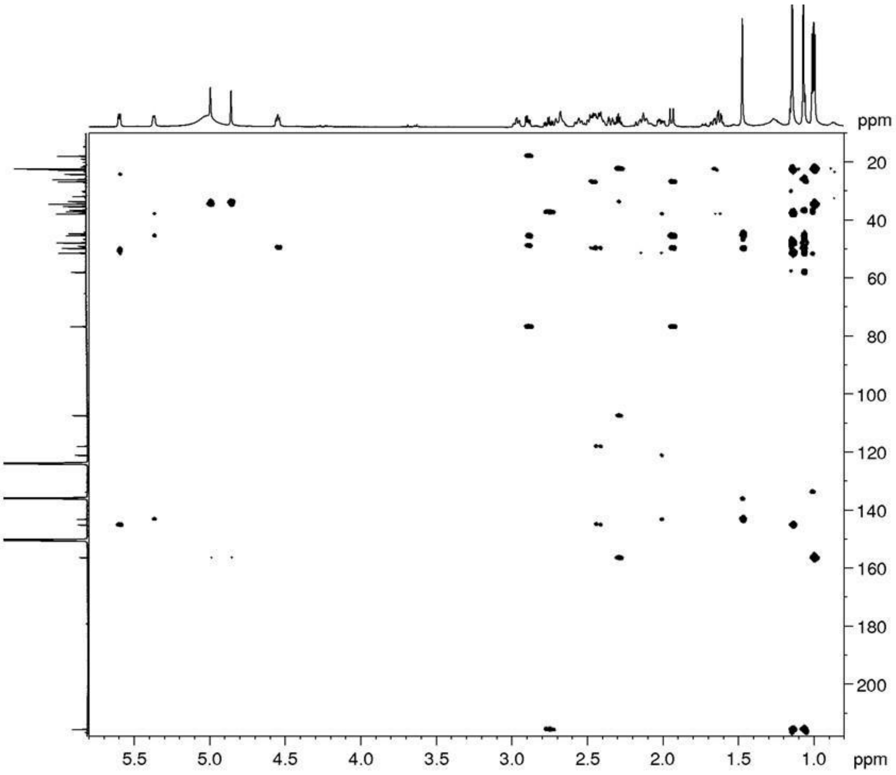

Figure S103. HMBC spectrum of compound **12** (600 MHz, pyridine-d_5, 295 K)

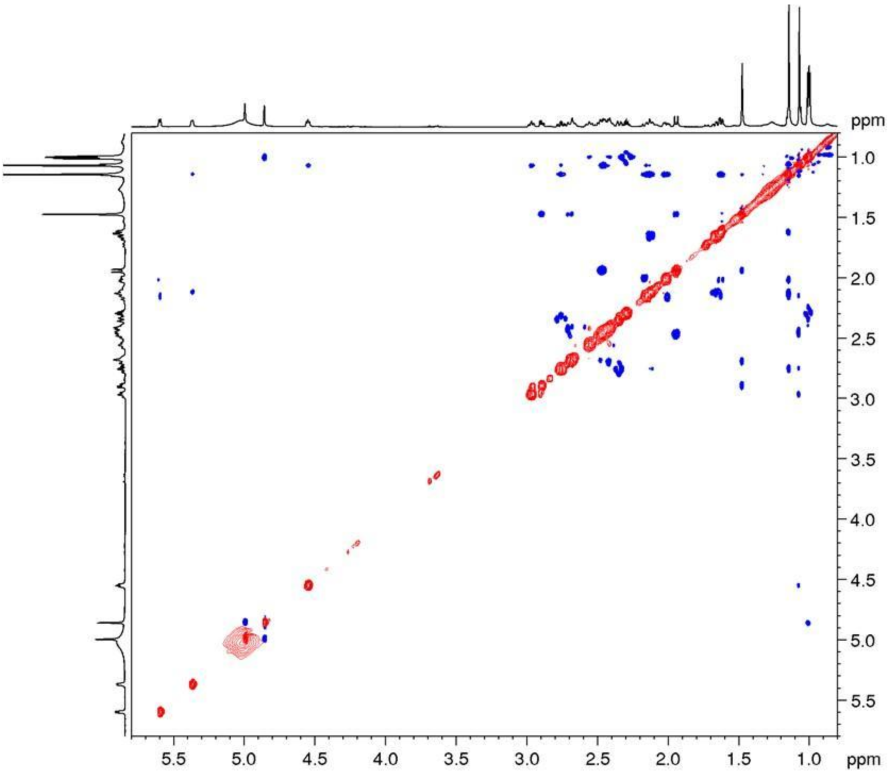

Figure S104. NOESY spectrum of compound **12** (600 MHz, pyridine-d_5, 295 K)

Table S1. NMR Spectroscopic Data (600 MHz, $CDCl_3$[a], pyridine-d_5[b], tetrahydrofuran-d_8[c], or methanol-*d*:pyridine-d_5 19:1[d], methanol-*d*[e]) for compounds **6-9**

	1[c]			2[c]			3[a]			4[a]			5[b]		
position	δ_C type		δ_H (*J* in Hz)	δ_C type		δ_H (*J* in Hz)	δ_C type		δ_H (*J* in Hz)	δ_C type		δ_H (*J* in Hz)	δ_C type		δ_H (*J* in Hz)
1	35.7	CH_2	1.82, m<br>1.54, m	36.4	CH_2	1.51, m<br>1.09, m	30.7	CH_2	1.43, m<br>1.37, m	30.6	CH_2	1.49, m<br>1.41, m	31.6	CH_2	1.75, m<br>1.55, m
2	36.8	CH_2	2.31, dd, (10.0, 8.0)<br>2.28, dd, (8.0, 3.4)	28.8	CH_2	1.48, m	23.1	CH_2	1.87, m<br>1.67, m	23.2	CH_2	1.90, m<br>1.72, m	24.1	CH_2	1.90, m<br>1.78, m
3	216.0	C		78.3	CH	2.97, m	77.6	CH	4.68, br s	80.6	CH	4.76, br s	78.4	CH	4.97, br s
4	52.8	C		39.7	C		36.7	C		36.8	C		37.4	C	
5	44.3	CH	2.17, m	51.5	CH	0.96, m	45.2	CH	1.50, m	45.3	CH	1.47, m	46.4	CH	1.83, m
6	20.2	CH_2	1.49, m<br>1.44, m	19.1	CH_2	1.65, m<br>1.49, m	17.8	CH_2	1.67, m<br>1.56, m	18.0	CH_2	1.59, m<br>1.49, m	18.8	CH_2	1.62, m<br>1.52, m
7	27.2	CH_2	2.03, m<br>1.93, m	27.2	CH_2	2.06, m<br>1.95, m	25.8	CH_2	2.12, m<br>2.07, m	25.9	CH_2	2.04, m	26.8	CH_2	2.17, m<br>2.12, m
8	136.6	C		135.8	C		135.0	C		134.2	C		135.5	C	
9	134.0	C		136.6	C		135.1	C		134.3	C		134.2	C	
10	37.6	C		37.8	C		36.7	C		36.8	C		37.5	C	
11	21.4	CH_2	1.94, m	40.5	CH_2	2.82, m<br>2.49, m	39.9	CH_2	2.90, m<br>2.71, m	20.9	CH_2	2.01, m	34.9	CH_2	2.76, m<br>2.50, m
12	30.0	CH_2	1.71, m<br>1.39, m	210.5	C		212.7	C		30.9	CH_2	1.74, m<br>1.69, m	72.5	CH	4.27, d (7.6)
13	46.5	C		55.0	C		54.5	C		44.5	C		50.5	C	
14	49.4	C		60.0	C		59.4	C		49.9	C		50.4	C	
15	44.0	CH_2	2.03, m<br>1.15, m	30.9	CH_2	1.75, m<br>1.20, m	30.1	CH_2	1.82, m<br>1.35, m	30.8	CH_2	1.60, m<br>1.18, m	33.1	CH_2	1.76, m<br>1.25, m
16	77.1	CH	3.89, m	28.6	CH_2	1.91, m<br>1.29, m	27.8	CH_2	2.01, m<br>1.37, m	28.2	CH_2	1.94, m<br>1.31, m	28.7	CH_2	2.11, m<br>1.40, m
17	57.3	CH	1.96, m	43.5	CH	2.06, m	42.4	CH	2.19, m	50.3	CH	1.50, m	43.5	CH	2.65, m
18	17.8	CH_3	0.69, s	12.9	CH_3	0.94, s	12.6	CH_3	1.05, s	15.7	CH_3	0.69, s	17.2	CH_3	0.77, s
19	19.2	CH_3	0.95, s	19.5	CH_3	0.98, s	19.0	CH_3	1.08, s	18.9	CH_3	1.00, s	19.4	CH_3	1.03, s
20	48.3	CH	2.24, m	37.7	CH	1.18, m	36.6	CH	1.27, m	36.3	CH	1.41, m	37.3	CH	1.53, m
21	177.4	C		19.6	CH_3	0.78, d (6.4)	19.0	CH_3	0.89, d (5.8)	18.6	CH_3	0.93, m	18.3	CH_3	1.28, m
22	31.6	CH_2	1.92, m<br>1.64, m	35.3	CH_2	1.53, m<br>1.14, m	34.0	CH_2	1.62, m<br>1.27, m	34.2	CH_2	1.58, m<br>1.19, m	35.4	CH_2	1.77, m<br>1.38, m
23	33.4	CH_2	1.96, m<br>1.87, m	32.9	CH_2	2.14, m<br>1.09, m	32.1	CH_2	2.21<br>2.02	31.7	CH_2	2.19, m<br>2.00, m	32.7	CH_2	2.37, m<br>2.17, m
24	156.7	C		150.9	C		148.5	C		148.3	C		150.2	C	
25	34.8	CH	2.14, m	46.2	CH	2.96, m	45.1	CH	3.17, m	45.0	CH	3.18, q (7.0)	46.2	CH	3.32, q (7.1)
26	22.4	CH_3	0.91, d (6.9)	175.6	C		177.7	C		179.6	C		175.3	C	
27	22.2	CH_3	0.92, d (6.9)	17.0	CH_3	1.10, d (7.4)	16.3	CH_3	1.31, d (6.7)	16.1	CH_3	1.31, d (7.0)	17.1	CH_3	1.38, d (7.1)
28	68.3	CH_2	3.39, dd (10.2, 2.4)	28.5	CH_3	0.88, s	27.5	CH_3	0.88, s	27.6	CH_3	0.88, s	28.5	CH_3	1.04, s

			3.16, dd (10.2, 2.4)												
29	17.5	CH_3	0.78, s	16.1	CH_3	0.69, s	21.7	CH_3	0.94, s	21.7	CH_3	0.93, m	22.4	CH_3	0.91, s
30	25.3	CH_3	1.05, s	24.3	CH_3	0.65, s	24.1	CH_3	0.82, s	24.2	CH_3	0.91, s	25.7	CH_3	1.43, s
31	106.9	CH_2	4.61, br s	110.3	CH_2	4.77, br s<br>4.71, br s	111.1	CH_2	4.97, br s<br>4.94, br s	111.4	CH_2	4.97, br s<br>4.94, br s	111.3	CH_2	5.10, br s<br>5.06, br s
1’							170.8	C		167.2	C		172.4[d]	C	
2’										40.2	CH_2	3.64, m	47.0[d]	CH_2	2.64[d], m
3’										170.6	C		71.0[d]	C	
4’													47.0[d]	CH_2	2.64[d], m
5’													172.4[d]	C	
1’-CH_3							21.3	CH_3	2.07, s						
3’-CH_3													27.9[d]	CH_3	1.37[d], m
26-CH_3													52.3[d]	CH_3	3.67[d], m

Table S2. NMR Spectroscopic Data (600 MHz, $CDCl_3$[a], pyridine-d_5[b], tetrahydrofuran-d_8[c], or methanol-*d*:pyridine-d_5 19:1[d], methanol-*d*[e]) for compounds **10-12**

	10[b]			11[a]			12[b]		
position	δ_C	type	δ_H (*J* in Hz)	δ_C	type	δ_H (*J* in Hz)	δ_C	type	δ_H (*J* in Hz)
1	31.1	CH_2	2.14, m	30.5	CH_2	1.48, m	37.1	CH_2	2.12, m<br>1.66, m
2	27.4	CH_2	2.05, m<br>1.82, m	23.2	CH_2	1.87, m<br>1.65, m	35.3	CH_2	2.75, m<br>2.34, m
3	75.5	CH	3.66, br s	77.9	CH	4.66, br s	215.6	C	
4	38.6	C		36.7	C		47.9	C	
5	45.1	CH	2.08, m	45.3	CH	1.50, m	51.4	CH	1.62, m
6	19.1	CH_2	1.74, m<br>1.61, m	17.9	CH_2	1.62, m<br>1.50, m	24.2	CH_2	2.15, m<br>2.01, m
7	27.0	CH_2	2.15, m	25.9	CH_2	2.04, m	121.1	CH	5.60, d (6.4)
8	135.3	C		134.6	C		143.2	C	
9	134.7	C		132.9	C		145.1	C	
10	37.8	C		36.7	C		37.9	C	
11	35.1	CH_2	2.81, m<br>2.52, m	32.6	CH_2	2.62, m<br>2.09, m	118.0	CH	5.37, d (6.0)
12	72.5	CH	4.28, d (7.5)	73.2	CH	4.00, d (8.0)	36.6	C	2.69, m<br>2.43, m
13	50.5	C		49.5	C		45.4	C	
14	50.4	C		49.6	C		49.7	C	
15	33.1	CH_2	1.76, m	32.0	CH_2	1.65, m<br>1.17, m	44.7	CH_2	2.46, m<br>1.94, m
16	28.8	CH_2	2.14, m<br>1.43, m	27.7	CH_2	2.02, m<br>1.35, m	76.7	CH	4.55, m
17	43.6	CH	2.68, m	43.1	CH	2.06, m	58.0	CH	2.89, m
18	17.2	CH_3	0.79, s	16.2	CH_3	0.61, s	18.0	CH_3	1.07, s
19	19.7	CH_3	1.11, s	18.7	CH_3	0.98, s	22.4	CH_3	1.14, s
20	37.3	CH	1.58, m	35.9	CH	1.42, m	48.9	CH	2.97, m
21	18.4	CH	1.31, d (6.1)	17.8	CH_3	1.02, d (6.4)	179.1	C	
22	35.5	CH_2	1.89, m<br>1.51, m	34.2	CH_2	1.63, m<br>1.24, m	31.8	CH_2	2.67, m<br>2.49, m
23	32.9	CH_2	2.60, m<br>2.34, m	31.8	CH_2	2.21, m<br>2.02, m	33.6	CH_2	2.56, m<br>2.41, m
24	151.1	C		148.5	C		156.4	C	
25	46.9	CH	3.51, m	45.3	CH	3.18, m	34.5	CH	2.29, m
26	177.4	C		178.5	C		22.2	CH_3	1.00, d (6.8)
27	17.5	CH_3	1.54, d (6.7)	16.2	CH_3	1.31, d (6.9)	22.2	CH_3	1.00, d (6.8)
28	29.5	CH_3	1.26, s	27.6	CH_3	0.87, s	26.0	CH_3	1.14, s
29	23.1	CH_3	0.97, s	21.8	CH_3	0.92, s	22.7	CH_3	1.07, s
30	25.6	CH_3	1.43, s	24.5	CH_3	1.12, s	26.7	CH_3	1.47, s
31	110.8	CH_2	5.25, br s<br>5.13, br s	111.1	CH_2	4.98, br s<br>4.94, br s	107.4	CH_2	5.00, br s<br>4.86, br s
1′				170.9	C				
1′-CH_3				21.4	CH_3	2.07, m			

Table S3. Chemosensitizing Activity of Compounds **3-4**, and **7-12** on Colo 320 Adenocarcinoma Cells

Compound	c(IC_{50}*4) [uM]	Drug:Doxo ratio	CI	SD	type
**3**	119.13	13.82:1	0.907	0.12	Nearly additive
		27.64:1	0.601	0.0495	Synergism
		55.28:1	0.972	0.081	Nearly additive
		110.56:1	1.027	0.1133	Nearly additive
		221.12:1	0.944	0.1212	Nearly additive
		442.24:1	1.471	0.3143	Strong antagonism
**4**	276.66	32.08:1	0.816	0.2102	Moderate synergism
		64.16:1	0.691	0.0705	Synergism
		128.32:1	0.734	0.0845	Moderate synergism
		256.64:1	0.739	0.092	Moderate synergism
		513.28:1	0.904	0.1805	Nearly additive
		1026.56:1	1.421	0.2691	Antagonism
**7**	246.84	28.63:1	1.002	0.0372	Nearly additive
		57.26:1	0.972	0.1029	Nearly additive
		114.52:1	0.419	0.0697	Synergism
		229.04:1	1.371	0.1618	Antagonism
		458.08:1	1.347	0.2608	Antagonism
		916.16:1	1.295	0.1921	Antagonism
**8**	144.74	16.8:1	0.905	0.0877	Nearly additive
		33.6:1	0.889	0.0332	Slight synergism
		67.2:1	0.574	0.0698	Synergism
		134.4:1	0.713	0.0972	Moderate synergism
		268.8:1	0.64	0.0956	Synergism
		537.6:1	1.042	0.0934	Nearly additive
**9**	157.84	18.23:1	1.316	0.21	Antagonism
		36.64:1	0.656	0.0528	Synergism
		73.28:1	0.653	0.0839	Synergism

		146.56:1	0.447	0.0534	Synergism
		293.12:1	0.559	0.0681	Synergism
		586.24:1	0.276	0.096	Strong synergism
**10**	195.9	22.72:1	1.258	0.3766	Antagonism
		45.44:1	1.09	0.1509	Nearly additive
		90.88:1	0.779	0.0819	Moderate synergism
		181.76:1	0.798	0.1561	Moderate synergism
		363.52:1	0.988	0.2678	Nearly additive
		727.04:1	1.438	0.3819	Antagonism
**11**	118.96	13.8:1	1.456	0.2324	Strong antagonism
		27.6:1	0.841	0.14	Moderate synergism
		55.2:1	0.911	0.2554	Nearly additive
		110.4:1	1.491	0.2423	Strong antagonism
		220.8:1	1.89	0.3337	Strong antagonism
		441.6:1	1.938	0.3863	Strong antagonism
**12**	82.85	9.62:1	1.992	0.3279	Strong antagonism
		19.24:1	1.42	0.2308	Antagonism
		38.48:1	0.608	0.0324	Synergism
		76.96:1	0.895	0.0678	Slight synergism
		153.92:1	0.736	0.0458	Moderate synergism
		307.84:1	0.824	0.2692	Moderate synergism

Range	Description
<0.1	Very strong synergism
0.1-0.3	Strong synergism
0.3-0.7	Synergism
0.7-0.85	Moderate Synergism

0.85-0.9	Slight synergism
0.9-1.1	Nearly additive
1.1-1.2	Moderate antagonism
1.2-1.45	Antagonism
1.45-3.3	Strong antagonism
3.3-10	Very strong antagonism

Doxo: doxorubicin, CI: combination index, SD: standard deviation

Table S4. P-gp Efflux Pump Inhibitory Activity of Compounds 1–12 on MDR Colo 320 Colon Adenocarcinoma Cell Line

Samples	conc. µM	FSC	SSC	FL-1	FAR
Tariquidar	0.2	1557	1038	45.800	15.091
Compound **1**	20	1696	898	2.350	0.774
Compound **2**	20	1603	947	3.190	1.051
Compound **3**	20	1239	889	13.500	**4.448**
Compound **4**	20	1349	930	4.060	1.338
Compound **5**	20	1332	1012	23.100	**7.611**
Compound **6**	20	1681	845	1.170	0.386
Compound **7**	20	1593	865	1.800	0.593
Compound **8**	20	1356	916	6.310	**2.079**
Compound **9**	20	1427	928	2.860	0.942
Compound **10**	20	1383	902	11.600	**3.822**
Compound **11**	20	1463	940	7.560	**2.491**
Compound **12**	20	1420	1007	6.880	**2.267**
DMSO	2%	1654	675	1.220	0.402

FSC: forward scatter count; SSC: side scatter count; FL-1: mean fluorescence; FAR: fluorescence activity ratios
